# COVID-19 and pro-sociality: How do donors respond to local pandemic severity, increased salience, and media coverage?

**DOI:** 10.1007/s10683-022-09753-y

**Published:** 2022-04-22

**Authors:** Maja Adena, Julian Harke

**Affiliations:** Wissenschaftszentrum Berlin für Sozialforschung, WZB Berlin, Reichpietschufer 50, 10785 Berlin, Germany

**Keywords:** COVID-19, Charitable giving, Online experiments, Natural experiments, C93, D64, D12

## Abstract

Has the COVID-19 pandemic affected pro-sociality among individuals? After the onset of the pandemic, many charitable appeals were updated to include a reference to COVID-19. Did donors increase their giving in response to such changes? In order to answer these questions, we conducted a real-donation online experiment with more than 4200 participants from 149 local areas in England and over 21 weeks. First, we varied the fundraising appeal to either include or exclude a reference to COVID-19. We found that including the reference to COVID-19 in the appeal increased donations. Second, in a natural experiment-like approach, we studied how the relative local severity of the pandemic and media coverage about local COVID-19 severity affected giving in our experiment. We found that both higher local severity and more related articles increased giving of participants in the respective areas. This holds for different specifications, including specifications with location fixed effects, time fixed effects, a broad set of individual characteristics to account for a potentially changing composition of the sample over time and to account for health- and work-related experiences with and expectations regarding the pandemic. While negative experiences with COVID-19 correlate negatively with giving, both approaches led us to conclude that the pure effect of increased salience of the pandemic on pro-sociality is positive. Despite the shift in public attention toward the domestic fight against the pandemic and away from developing countries’ challenges, we found that preferences did not shift toward giving more to a national project and less to developing countries.

**Supplementary Information:**

The online version contains supplementary material available at 10.1007/s10683-022-09753-y.

## Introduction

Locally occurring natural catastrophes seem to increase international solidarity (Scharf et al., 2022). However, the global spread of COVID-19 has been unprecedented, meaning that it is not clear what types of behavioral responses it will generate. Anecdotal evidence tells of helpful neighbors who go shopping for the vulnerable, donate food, or sew homemade face coverings for nursing homes.^1^ Other individuals have been less benevolent: Some have even gone as far as engaging in racist attacks on members of ethnic groups who have been blamed for spreading the disease (Devakumar et al., 2020; Lu and Sheng, 2021). Moreover, since the beginning of the COVID-19 pandemic, attention has shifted away from once-prominent concerns, including the refugee situation and famine in developing countries. To regain attention, many charities started using references to COVID-19 in their solicitations, even when asking for donations for projects that are not directly related to the pandemic.

In this project, we set out to understand how pro-sociality has changed during the COVID-19 pandemic. In order to answer this question, we adopted a twofold approach. First, in an online experiment, participants saw a donation ask for Save the Children. For the treatment group, we added a reference to COVID-19 to make the pandemic more salient. The additional paragraph pointed to the negative consequences of the COVID-19 pandemic faced by children but did not refer to any pandemic-specific relief actions by the charity. This approach was meant to draw the attention of readers to the pandemic while minimizing any other differences between the two appeals.^2^ The participants subsequently divided an additional bonus between their own account and a donation. This approach allowed us to provide clean causal estimates of the COVID-19 reference in the appeal. Second, in a natural experiment-like approach, we explored differences across local areas and time in the relative local severity of the pandemic and the extent of media coverage about local COVID-19 severity. We exploited the variation of COVID-19 severity and media coverage in each of the 149 English Upper Tier Local Authorities (UTLA) over 21 weeks. We analyzed whether those differences could explain the variation in donations collected in the online experiment. Importantly, in the most conservative specification, we controlled for time fixed effects, location fixed effects, and accounted for a potentially changing composition of the sample by controlling for an extensive set of individual characteristics of participants. This means that our findings cannot be explained by time effects such as countrywide economic trends or changes in media attention (e.g. due to the occurrence of other natural disasters). Our findings likewise cannot be explained by a correlation between the share of COVID-19 cases and location-specific characteristics. They are also independent of socioeconomic factors, changes in individual work-related or financial situations, and health conditions due to the COVID-19 pandemic, changes in expectations for the development of these factors in the future. Consequently, we interpret our results as the effect of a relative increase in the pandemic’s severity and pandemic awareness.

Our results show that appeals with a COVID-19 reference increased charitable giving. This confirms that the strategy used by charitable organizations to include references to the pandemic likely paid off and that intuition of fundraisers was correct.^3^ We also found that higher relative local severity of the pandemic as reflected by a greater share of individuals testing positive for COVID-19 resulted in more giving in the experiment by participants from respective areas. Similarly, more media coverage about local COVID-19 severity increased giving as well. This shows that, overall, increased salience of the COVID-19 pandemic has made people more willing to help less fortunate individuals. This holds despite the fact that those facing more negative health and economic consequences donated less on average. The findings in the experimental part and the results related to media coverage suggest that the attention shift toward COVID-19 is one of the important channels by which the pandemic affected pro-sociality.

In the experiment, after the donation decision, participants were asked to divide their donation between Save the Children’s UK and global programs. Although we conjectured that the attention shift toward the pandemic, due to both the COVID-19 reference and the higher incidence, would shift donations toward the national project, we failed to reject the null hypothesis of no effect. This suggests that the pandemic had not made people in the UK more nationally oriented in this respect.

The number of economic studies on COVID-19 is increasing rapidly, though papers relating to pro-sociality and giving in this context remain rather scarce. This is surprising given the dramatic effects of the pandemic. The health and economic situations of millions of people in both poor and rich countries have been negatively affected: People have lost or are at risk of losing their work and income, are at risk of falling into extreme poverty, and face hunger. This extreme situation requires global solidarity in order to lessen the health, social, and economic consequences of the pandemic.

Related studies in the field of COVID economics include Brañas-Garza et al. (2022). In an online survey conducted during a six-day window, the authors asked participants from southern Spain to divide a €100 prize between themselves and a donation. They found that participants aged 30 years and older had decreased their giving significantly between the first and last three days of the survey,^4^ which they relate to the increase in the number of COVID-19 cases over time. It is, however, not clear whether this change in average donations was related to different reactions to the increasing number of cases, to the more pronounced economic consequences, or to other differences over time. Abel and Brown (2020) experimentally studied how COVID-19-related behaviors of crowds and public officials presented in the media, like mask wearing or distancing measures, affect charitable giving and volunteering. They found that watching a short clip that depicted positive behavior of crowds or negative behavior of public figures increased pro-sociality, while depictions of negative behavior of crowds and positive behavior of public figures decreased pro-sociality. Abel et al. (2021) found that debiasing people’s own risk perceptions did not affected donations to a COVID-19 emergency fund but did decrease the amount of time invested in learning how to protect older people. However, providing information on the risks faced by older people helped to counteract these negative effects. A study by Campos-Mercade et al. (2021) shows that more pro-social individuals are more likely to follow physical distancing guidelines, stay home when sick, and purchase face masks.^5^ In addition to our paper’s relevance to the field of COVID economics, we contribute more broadly to the literature on the impact of extreme circumstances on individual behavior. While there are a number of experiments that study behavior under laboratory-induced stress,^6^ real-world (causal) studies are especially scarce (Kowalski-Trakofler et al. 2003).^7^ Charitable giving and humanitarian aid in the aftermath of natural disasters has been studied by Strömberg (2007); Eisensee and Strömberg (2007); Jayaraman et al. (2021); Scharf et al. (2022). Understanding the effects of extreme circumstances like natural catastrophes and conflict situations on pro-sociality and generosity is crucial, as not all governments are able or willing to support people in need and international relief may be limited in such contexts.

Natural experiment-like approaches have been used to study the effect of natural disasters on charitable giving by, among others, Deryugina and Marx (2021). Online experiments to study pro-sociality have been applied by, among others, Chen et al. (2010), Exley and Petrie (2018), Goette and Tripodi (2018), Diederich et al. (2021).

We proceed as follows. In Sect. 2, we present the design of the experiment and our hypotheses. In Sect. 3, we describe the analysis and main results. Sect. 4 concludes.

## Design of the experiment and hypotheses

It is challenging to arrive at clean causal estimates of the effects of the COVID-19 pandemic on pro-sociality. Comparing giving decisions over time (before, during, and after the pandemic) would not be reliable, since additional time factors other than the spread of COVID-19 could influence the behavior under study. When comparing more with less affected areas, various correlations might seriously bias the estimates: Areas with more highly skilled workers might be less economically affected because highly skilled workers are more likely to switch to remote work. In tourism-dependent and economically underdeveloped areas, workers might be more likely to lose work or to receive lower remuneration.

Even if a study could overcome the aforementioned challenges of empirical identification, it would face additional problems because the pandemic as such likely affects pro-sociality through different (potentially competing) channels. These channels might include, a growing awareness of the pandemic; a deterioration in respondents’ own economic situations, health, or the health of close family members; or respondents’ fears about the future. While we expected the first factor—awareness of the pandemic—to increase solidarity, the remaining factors (especially the economic ones) could reduce the willingness to give. The exact timing of decision making or regional specifics may determine which of those factors prevails. While at the beginning of the pandemic, people may not have experienced negative effects on their individual economic situations, this might be the case in later periods. Likewise, the opposite could be the case: The feared negative consequences might not have come to pass in the expected way.

Regarding the economic and health channel as well as the role of fears, we can only get as close as to correlational evidence, since we cannot exogenously vary these factors. Therefore, in this paper, we set out to causally identify one of the channels: awareness of the pandemic. For this reason, we used two complementary approaches: an online experiment and a natural experiment.

Our first approach made use of an online experiment in order to test the role of experimentally induced attention shift. In the donation appeal, we adopted a strategy similar to those recently used by charities and provided additional information to direct participants’ attention toward the COVID-19 pandemic, while supplying the control group with an appeal with no COVID-19 references whatsoever. We made sure that the donation ask was a general one in both conditions, since participants might have been more (or less) likely to donate to a more specific project (Kessler et al., 2019).

Because our study employs randomization, the approach we chose ensures that factors pertaining to individuals’ economic and health-related situations should be equal between the control and treatment groups (we test this in Table A1 in the online appendix). The only remaining factor is the exogenous attention shift, which we expected to increase solidarity. This led us to formulate the following hypothesis:

**H1**: The COVID-19 reference increases donations.

In order to better understand the mechanism behind the potential treatment differences, we employed an additional survey experiment with new participants from the same subject pool who answered a number of unincentivized questions regarding their perceptions of the appeal after reading either the control or treatment version. In the online appendix, Section F, we describe the design of this additional survey experiment in more detail.

Our second approach made use of a natural experiment: The severity of the pandemic developed differently in local areas over time. In the post-experimental survey, we asked participants for their (self-reported) area of residence, which we matched to the Upper Tier Local Authority (UTLA) for which COVID-19 cases were available in England.^8^ While each individual participated only once in the experiment, over 21 weeks, all 149 local areas were represented in the experiment. We studied England because it was one of the most affected countries in Europe at the time, with over 1.2 million cases and 50,000 deaths related to COVID-19 by the end of November 2020, and because it offered good local data availability. Figure A3 in the online appendix shows the number of cases per day and the dates of the experimental sessions. We collected the numbers of lab-confirmed cases of COVID-19 for each local area over time.^9^ Given different testing strategies and thus a different meaning of the absolute number of cases at the beginning of the pandemic versus later on, we used a relative measure: the share of cases in a specific UTLA up to date, relative to all cases. However, we tested the robustness of using different absolute measures as well. On top of this, we controlled for an extensive set of individual characteristics that accounts for the potentially changing composition of the sample over time. We also tested the sensitivity of the estimate to those characteristics, which also helped us to assess the potential effect of unobservables. Controlling for time fixed effects, location fixed effects, health and work-related experiences, financial characteristics, and changing expectations allowed us to distill the effects of local severity that do not work through economic channels or expectations, such as a deterioration in respondents’ own economic situations, health, or the health of close family members; or respondents’ fears about the future.

Given that we expected the pandemic to affect pro-sociality via a variety of competing factors, we had no prior expectations regarding the direction of the overall effect and formulated an open hypothesis:

**H2A**: Individuals in relatively more affected areas give more than individuals in relatively less affected areas.
**H2B**: Individuals in relatively more affected areas give less than individuals in relatively less affected areas.

In a similar vein, we studied the effect of media coverage about local COVID-19 severity. We searched through articles published within the seven days prior to each experimental session in the online editions of 13 daily newspapers, plus the corresponding Sunday edition if available (The Times, The Sunday Times, The Independent, The Telegraph, The Guardian, Observer Magazine, i, Daily Express, Sunday Express Mag, Daily Mail, Daily Mirror, The Sunday Mirror, The Sunday People, Daily Record, Sunday Mail, Daily Star, Daily Star Sunday, The Sun, London Evening Standard, Metro), as well as on BBC Online. The search query was (“covid” OR “corona” in article title) AND ((“cluster” OR “hotspot” OR “hot spot”) AND (“infection” OR “case” OR “spread”) AND (location name) within a three-sentence range). This search resulted in more than 5,800 articles (see Figure A4 showing the distribution of articles over time and local area).

Most studies on local versus global preferences in charitable giving suggest that donors prefer local goals, but some show the opposite,^10^ and there are many well-supported charities pursuing global projects. In this study, we did not so much seek to answer the question of local versus global preferences in charitable giving. We rather hypothesized that the global pandemic and related media coverage shift individuals’ attention from distant problems toward more local goals. Consequently, we expected donations to shift from global to local causes. For the above described treatment condition, which shifts individuals’ attention toward COVID-19, we formulated the following hypothesis:

**H3**: The national project benefits more from the COVID-19 reference than the global project.

In a similar vein, we expected that:

**H4**: Individuals in more affected areas shift their giving more to local causes than those in less affected areas.

We implemented a donation experiment on Prolific with 4,211 participants whose area of residence was indicated to be in England.^11^ We did not apply any other pre-screening criteria but excluded individuals with missing values for the following baseline variables as provided by Prolific: age, gender, socioeconomic status, household size, household income, and country of birth. (For more details, see Section D of the online appendix.)

The subjects received a fixed participation fee of $\pounds$1.70^12^ and an additional budget of $\pounds$1 to be divided between their own account and a donation.^13^ We ran the experiment for 21 weeks, from June until August and from October until November 2020.^14^ On each occasion, there was one session per week, on Monday evenings.^15^

In the control group, participants received a donation ask for Save the Children. In the treatment group, participants received the same donation ask with an additional paragraph about COVID-19. The additional paragraph read: “The coronavirus is already having devastating consequences for children and their rights. Health systems, both in poor countries and the NHS, are being overwhelmed. Children have had their education disrupted by school closures. Many face the prospect of poverty. With the pandemic now spreading into some of the world’s poorest countries and in the UK, there is a real danger that we will see a reversal of the gains made over the last 20 years. There is an alternative.”^16^ In the first step, participants were asked to divide the additional budget of $\pounds$1 between their own account and a donation to Save the Children by using a slider^17^ (see Fig. A1 and A2 in the online appendix for the exact implementation). In the second step, we asked participants to divide their chosen donation amount between a project aiming to help children in the UK versus one aiming to help children in developing countries. Again, participants indicated their decision by using a slider. All donation decisions were implemented ex post. For non-donors, we modified the division question. These non-donors were informed that the researchers would donate an additional $\pounds$100 to Save the Children UK after the end of the study and were asked to indicate how they wished to divide this donation between Save the Children’s UK program and the global program. The donation division of the additional $\pounds$100 was implemented according to the average decision made by all non-donors.

After making the two donation decisions, participants were asked to fill in a short survey. In the subsequent analysis, we excluded participants with three or more inconsistent or illogical responses following a preregistered protocol (see Section C of the online appendix). This resulted in the exclusion of around 16% of the initial sample in the following analysis. For the exact formulation of the experimental protocol and the questions, see online appendix, Section G. The hypotheses and analysis were preregistered at OSF (https://osf.io/h5syz/) before data collection began.^18^

## Analysis and results

### Descriptive statistics

**Table 1:** Descriptive statistics for the outcome variables

	(1) Overall			(2) By treatment					
				(a) Control			(b) COVID-19 reference		
	Mean	Std. error	N	Mean	Std. error	N	Mean	Std. error	N
*Donation choice*									
Overall amount (i)	.5951607	.0068394	3548	.5711618	.0096508	1799	.6198456	.0096605	1749
Share of donors	.7669109	.0070991	3548	.7487493	.0102289	1799	.7855918	.0098163	1749
Positive amount	.7760492	.0052849	2721	.7628211	.0075857	1,347	.7890175	.0073502	1374
*Donation share to UK project*									
Overall	.5516911	.0054728	3548	.5533463	.0078258	1799	.5499886	.0076482	1749
If donation positive (ii)	.5408232	.0061844	2721	.5504009	.0089523	1347	.5314338	.0085378	1374
If donation equal to zero	.5874486	.0116364	827	.5621239	.0160857	452	.6179733	.0166989	375

(i) and (ii) mark the outcomes used in the (preregistered) hypotheses tests

Table 1 shows summary statistics for the two decisions that participants made in the experiment. The first decision is shown in the upper panel and the second decision in the lower panel. The overall averages are shown in part (1) of the table. Out of the additional budget of $\pounds$1, participants donated, on average, 60 pence. Although the donation could be any amount between $\pounds$0 and $\pounds$1, many participants exhibited preferences for more focal numbers, especially 0, 1, and 0.5 but also 0.1, 0.2, and so on. The share of participants who donated positive amounts is 77%. They directed 55% of their donation to the UK project. Part (2) of Table 1 presents the averages by treatment condition. The average donation amount, the share of donors, and the average positive amount are higher in the treatment group. In the treatment condition, the share of donations directed to the UK project is lower for donors and higher for non-donors.

Figure A5 in the online appendix provides an example of how our measure of the relative local severity of the pandemic—the share of COVID-19 cases in a UTLA to date, relative to all cases in England—and donation amounts vary over time for the four local areas with the greatest number of individual observations in our data (Kent, Birmingham, Hertfordshire, and Lancashire). Note that the graphs are based on small sample sizes (between 92 and 125 observations), so we do not draw any direct conclusions from them. They are meant to give an idea of the data at hand. We see that while in the early weeks, Kent, Hertfordshire, and Essex were relatively more affected by the pandemic, in the later weeks, they had lower case shares than before and compared to other local areas. The opposite held for Birmingham. For Kent and Essex, the average donation seemed to follow the pattern of the pandemic’s severity, while for the remaining local areas, patterns were more diffuse. In the following subsections, we proceed with the tests of our hypotheses.

### Treatment effect on donation levels

For H1 regarding the effect of the treatment condition on donation choices, we ran a regression of the following form:

1 $$\begin{aligned} {d}_{i,t}=\alpha + {\beta }_{1}{T_{i}} + {\beta }_{2}{X_{i}} + {\theta _t}+ {\epsilon }_{i} \end{aligned}$$

where *d* denotes a donation amount, the subscript *i* denotes an individual, *T* denotes the treatment condition, *X* denotes a vector of control variables, ${\theta _t}$ denotes time dummies, and ${\epsilon }_{i}$ is the error term. Table 2 shows the results. In all columns, we include baseline controls and time dummies. Baseline controls consist of the initial position of the slider,^19^ age, female dummy, socioeconomic status, household size, and dummy for being born in the UK, as provided by Prolific.

The second set of controls in Column (2) and (3) includes participants’ financial situations and their expectations for the future. For household income, we created a continuous variable based on the mid-values of income categories provided by Prolific and, wherever the participant chose “prefer not to say,” imputed mid-values of the income category gathered through our survey.^20^ Further financial variables from our survey included dummies for making ends meet before and since the onset of the COVID-19 pandemic, dummies for how income has been affected by the COVID-19 pandemic, and dummies for how participants expected their income to be affected in the future.

The third set of controls in Column (3) relates to participants’ health and includes answers to the questions regarding whether their health has been affected by the COVID-19 pandemic, whether they expected their health to be affected in the future, and whether they were a member of a risk group.

**Table 2:** H1: The COVID-19 reference increases donations. Outcome variable: donation amount

	(1)	(2)	(3)
COVID-19 reference	0.052*** (0.013)	0.053*** (0.013)	0.050*** (0.013)
Baseline controls	Yes	Yes	Yes
Financial controls	No	Yes	Yes
Health controls	No	No	Yes
Time fixed effects	Yes	Yes	Yes
Observations	3541	3541	3541
$R^{2}$	0.050	0.059	0.067

Robust errors. Baseline controls are slider initial position, age, dummy born in the UK, female dummy, socioeconomic status, and number of household members. Financial controls include monthly household income, making ends meet dummies (before the pandemic and since the pandemic), and income change dummies (since the pandemic and expected in the future). Health controls include health negatively affected by COVID-19 dummies, expected negative impact on health dummies, and vulnerability to COVID-19: high risk or moderate risk dummies

*p<0.10, **p<0.05, ***p<0.01

The results confirm hypothesis H1 in that the reference to COVID-19 in the charity appeal increased donations. The increase is around 5 pence, from an average of 57 pence in the control group; this represents an increase of around 8%. Since the donations are bound between zero and one, we additionally present the results from a two-limit Tobit specification in the online appendix, Table A2. The analysis suggests an average marginal effect of 15 pence, which corresponds to an increase in giving of as much as 20%.

In order to put these effects into perspective, we looked at the differences in giving by gender and age, and by the variables that reflect experiences with the pandemic as well as expectations for the future. In Table A3 in the online appendix, we present the average donations by those different variables. We observed that females give 12 pence more on average and that giving increases with age, with those over 65 giving 16 pence more than those aged 18–24. We also found a clear pattern in making ends meet before and since the COVID-19 pandemic, with those who report less difficulty in making ends meet giving more. For income changes, health changes, and expected changes in the future, however, we instead see an inverted, U-shaped pattern. Those whose health had been somewhat affected or who expected their health to be somewhat affected in the future and those whose income had stayed the same or who expected it to stay the same in the future gave the largest amounts compared with those who had had or expected positive or negative income changes or whose health had been or was expected to be strongly affected as well as those who had experienced or expected no effects on health. For example, the magnitudes of the differences in our treatment are similar to the difference between average giving of those who had experienced some difficulties in making ends meet versus those who had been able to make ends meet fairly easily before the COVID-19 pandemic.

In an additional survey experiment, we found no significant differences in how the charitable project was perceived after receiving different donation appeals.^21^ In the treatment condition, participants did not expect the money collected for the project to be spent sooner, and they did not consider the project to be more urgent, effective, or important. We found that the donation appeal with the reference to COVID-19 did not evoke more negative emotions in the participants but that it did evoke less positive emotions, though this difference is only significant at p<0.1 and does not survive corrections for multiple hypothesis testing. In line with the priming nature of the COVID-19 treatment, some participants in the treatment condition mentioned COVID-19 relief as one of the goals of the project, while none did so in the control group. Participants did not report significantly higher pressure to donate to an appeal with a COVID-19 reference, and this was similar for a real-life situation and for the ask when participating in a study on Prolific. Therefore, we conclude that the effect of the treatment condition is due neither to perceived differences in the project nor due to an experimenter demand effect (Zizzo, 2010). The effect is rather due to the increased salience of the COVID-19 pandemic.

### The effect of local pandemic severity on donation levels

Before we proceed with the actual analysis, we show that our measure of relative local severity is strongly correlated with subjective perceptions. In the survey, we asked, “In your opinion, is the COVID-19 pandemic more or less severe in [participant’s local area] than in other areas in England?” In response, participants could choose between “more severe,” “equally severe,” or “less severe.” In Table A4 in the online appendix, we regress those subjective perceptions on our measure of relative local severity. All columns include time fixed effects and location fixed effects. The results show that higher local severity makes people more likely to select “more severe” as an answer to the subjective question and less likely to select “less severe.” This confirms that the chosen variable measures what it is intended to measure while clearly remaining objective at the same time.

For H2 regarding the effect of local pandemic severity on donation levels, we ran a regression of the following form:

2 $$\begin{aligned} {d}_{i,j,t}=\alpha + {\beta }_{1} {P_{j,t}} + {\beta }_{2} {X_{i,(j)}} + ({\delta _j})+{\theta _t}+ {\epsilon }_{i} \end{aligned}$$

where *j* denotes the area in which the individual lives, *P* denotes relative local pandemic severity, and ${\delta _j}$ are location fixed effects. While for H1 the controls serve to increase precision, here the choice of controls might be crucial for the size and sign of the ${\beta }_{1}$ coefficient due to correlations between those variables with both pandemic severity and donation values. In Table 3 across all columns, we include controls for the baseline individual characteristics and time fixed effects as specified in the previous subsection. In Column (2), we add financial and health controls, again as previously specified. In Column (3), we account for the economic aspects of the area (wages, working hours, job density, share of employees in different sectors of the economy), and aspects of the area that might influence COVID-19 health risks (number of hospitals, age structure, population density, average health status indicators).^22^ Column (4) exchanges area controls for location fixed effects.

**Table 3:** H2: Individuals in more affected places will give more (or less) than individuals in less affected places. Outcome variable: donation amount

	(1)	(2)	(3)	(4)
Relative local severity of the pandemic	$$0.023^{**}$$ (0.010)	$$0.021^{**}$$ (0.010)	$$0.054^{**}$$ (0.022)	$$0.110^{***}$$ (0.037)
COVID-19 reference	$$0.053^{***}$$ (0.013)	$$0.051^{***}$$ (0.013)	$$0.050^{***}$$ (0.014)	$$0.043^{***}$$ (0.014)
Baseline controls	Yes	Yes	Yes	Yes
Financial controls	No	Yes	Yes	Yes
Health controls	No	Yes	Yes	Yes
Area controls	No	No	Yes	No
Location fixed effect	No	No	No	Yes
Time fixed effects	Yes	Yes	Yes	Yes
Observations	3525	3525	3423	3525
$R^{2}$	0.052	0.069	0.080	0.118

Robust errors. All columns include the following baseline controls: slider initial position, age, dummy born in the UK, female dummy, socioeconomic status, number of household members, and session dummies (time fixed effects). Financial controls include monthly household income, making ends meet dummies (before the pandemic and since the pandemic), income change dummies (since the pandemic and expected in the future). Health controls include health negatively affected by COVID-19 dummies, expected negative impact on health dummies, and vulnerability to COVID-19: high risk or moderate risk dummies. Area controls include dummies for shares of different age groups; population density; dummies for shares of people with good, fair, and bad health; job density; mean annual pay for full-time workers; mean hourly pay for full-time workers; mean work hours for full-time workers; number of National Health Service hospitals per 100 inhabitants; and shares of employees in different sectors of the economy

*p<0.10, **p<0.05, ***p<0.01

The coefficient on relative local severity of the pandemic suggests that an additional 1% of cases results in an increase in donations by 2 pence (in the specifications with individual characteristics), 5 pence (with location characteristics), or 11 pence (with location fixed effects). Those stark differences in the estimated coefficients suggest that local characteristics are correlated with both local severity and donations. This is only partly corrected when accounting for a large number of observable location characteristics but is taken care of in Column (4) in which we included location fixed effects.

In the online appendix, we provide a number of robustness checks. First, we show that it is unlikely that we have missed any other important explanatory variable which could have biased our results. In Table A10, we show an exercise in which we gradually included different sets of control variables. In Columns (2)-(6), we control for location fixed effects, time fixed effects, and baseline characteristics of the individuals to account for compositional changes of the sample over time. The gradual inclusion of additional individual characteristics as well as a large set of variables reflecting experiences with COVID-19 does not lead to any meaningful change in the coefficient of interest. In the spirit of Oster (2019) and Altonji et al. (2005), under the assumption that unobservables are correlated with observables, we conclude that the unobservables are unlikely to have biased our estimates. In Table A6, we additionally control for an interaction between time dummies and nine region dummies to account for potential region-specific trends that could have affected both local severity and individual economic situation or other aspects potentially correlated with giving. The coefficient on local severity is similar to that in our preferred specification in Table 3, Column (4).

Second, in Table A5, we show the results after applying a two-limit Tobit. The estimated average marginal effects are now in the range of 6–33 pence, depending on the specification.

Third, in Table A7, we include the interaction effect between relative local severity and the treatment. The interaction effect is not significant, meaning that the effect of relative local severity is not amplified (or diminished) by the additional attention shift created by our treatment.

Fourth, we also test the robustness of our local severity measure. In Table A8, we replace the relative measure with the absolute number of COVID-19 cases in the seven days prior to each session of the experiment (scaled by 1,000). In Table A9, we replace it with the same number but measured per 100 inhabitants. The results are in line with those presented in Table 3: Local pandemic severity increases giving in the experiment.

What is the channel from increased local severity to higher giving in our experiment? Table A11 shows the correlations between subjectively reported experiences with COVID-19 and expectations and giving in our experiment. Negative experiences and expectations correlate negatively with giving. However, in Column (4) of Table 3, we control for individual health and financial situation as well as for the characteristics of the local area. Therefore, we interpret our results as the pure effect of increased awareness about COVID-19, similar to what we found when including the COVID-19 reference in the treatment condition. In order to strengthen this interpretation, we collected additional data. We expected that the media should play an important role in influencing the awareness of the pandemic. Therefore, we searched through articles in national newspapers and on BBC Online for reports about local severity. We counted the number of articles per local area in the week prior to the experiment. We ran the same regressions as in Table 3 using the number of articles (scaled by 10) as an explanatory variable in place of the variable capturing local severity. We present the results in Table 4. Confirming our above conjecture, we found the effect of media coverage about the local COVID-19 severity on donations in the experiment to be positive and significant.

**Table 4:** Number of articles about outbreaks for a specific location and donations in the experiment. Outcome variable: donation amount

	(1)	(2)	(3)	(4)
Articles	$$0.036^{**}$$ (0.014)	$$0.037^{**}$$ (0.014)	$$0.041^{***}$$ (0.016)	$$0.045^{***}$$ (0.017)
COVID-19 reference	$$0.053^{***}$$ (0.013)	$$0.051^{***}$$ (0.013)	$$0.049^{***}$$ (0.014)	$$0.044^{***}$$ (0.014)
Baseline controls	Yes	Yes	Yes	Yes
Financial controls	No	Yes	Yes	Yes
Health controls	No	Yes	Yes	Yes
Area controls	No	No	Yes	No
Location fixed effect	No	No	No	Yes
Time fixed effects	Yes	Yes	Yes	Yes
Observations	3525	3525	3423	3525
$R^{2}$	0.052	0.069	0.080	0.117

See notes to Table 3. The variable Articles is scaled by 10 to ease the readability of the coefficient

*p<0.10, **p<0.05, ***p<0.01*

### Treatment effect on donation destination

We tested H3 regarding the split of donations between the UK and the global program in the following regression:

3 $$\begin{aligned} {ds}_{ UK ,i,t}= \alpha + {\beta }_{1}{T_{i}} + {\beta }_{2}{X_{i}} + {\theta _t} + {\epsilon }_{i} \end{aligned}$$

where ${ds}_{ UK ,i}$ is the donation share devoted to Save the Children’s UK program conditional on the donation being positive. Table 5 shows the results. The control variables in Columns (1)–(3) include those specified for H1 with the difference regarding the initial position of the slider: Here, this applies to the second decision. In Table 5, we restrict the sample to participants who donated positive amounts. In Table A12 in the online appendix, we combine donor and non-donor division decisions.^23^ Although we hypothesized that the treatment effect would be positive on the share of donations devoted to the UK program, we failed to reject the null hypothesis of no effect. There simply seems to be no effect of the COVID-19 reference on the preference for the national program.

In Column (4), we additionally include variables that are likely to be correlated with individual decisions regarding the preferred destination for donations. In the post-experiment survey, we asked participants to estimate gross domestic product (GDP) growth and growth of the poverty rate in 2020 for the UK and for developing countries. The results suggest that those who thought that the UK was better off in 2020 relative to developing countries in terms of GDP growth donated less to the UK program. Those who thought that the poverty rate in the UK was higher than that in developing countries donated more to the UK program. Finally, participants donated more to the UK program if they thought that the UK was being more affected or equally affected by the pandemic than developing countries.

**Table 5:** H3: The national project will benefit more from the COVID-19 frame than the global project. Outcome variable: donation share to the UK program

	(1)	(2)	(3)	(4)
COVID-19 reference	− 0.017 (0.012)	− 0.017 (0.012)	− 0.015 (0.012)	− 0.013 (0.012)
GDP in the UK vs. in developing countries				− 0.003** (0.001)
Poverty in the UK vs. in developing countries				0.004*** (0.001)
UK more affected dummy				0.057*** (0.016)
UK equally affected dummy				0.059*** (0.016)
Baseline controls	Yes	Yes	Yes	Yes
Financial controls	No	Yes	No	Yes
Health controls	No	No	Yes	Yes
Time fixed effects	Yes	Yes	Yes	Yes
Observations	2715	2715	2715	2715
$R^{2}$	0.096	0.104	0.104	0.121

Robust errors. All columns include the following baseline controls: slider initial position, age, dummy born in the UK, female dummy, socioeconomic status, number of household members, and session dummies (time fixed effects). Financial controls include monthly household income, making ends meet dummies (before the pandemic and since the pandemic), and income change dummies (since the pandemic and expected in the future). Health controls include health negatively affected by COVID-19 dummies, expected negative impact on health dummies, and vulnerability to COVID-19: high risk or moderate risk dummies

*p<0.10, **p<0.05, ***p<0.01

### The effect of local pandemic severity on donation destination

We estimate the effect of the severity of the pandemic on the donation share to the UK program by running the following regression:

4 $$\begin{aligned} {ds}_{ UK ,i,j,t}=\alpha + {\beta }_{1}{P_{j}} + {\beta }_{2} {X_{j/i}} + {\delta _j}+{\theta _t}+ {\epsilon }_{i} \end{aligned}$$

Table 6 shows the results structured as in Table 3 with the following exceptions: (i) The outcome variable is the share of donations to the UK program. (ii) The control variables include the initial position of the slider at the second decision. (iii) The sample is restricted to donors only. In Table A13 in the online appendix, we show the results when combining donor and non-donor division decisions. Although we hypothesized that the effect of relative local severity of the pandemic would be positive on the share of donations devoted to the UK program, we failed to reject the null hypothesis of no effect.

**Table 6:** H4: Individuals in more affected places will shift their giving to local causes more than those in less affected places. Outcome variable: donation share to the UK program

	(1)	(2)	(3)	(4)
Relative local severity of the pandemic	0.002 (0.009)	0.002 (0.009)	0.008 (0.019)	− 0.024 (0.030)
COVID-19 reference	− 0.017 (0.012)	− 0.016 (0.012)	− 0.014 (0.012)	− 0.009 (0.012)
Baseline controls	Yes	Yes	Yes	Yes
Financial controls	No	Yes	Yes	Yes
Health controls	No	Yes	Yes	Yes
Area controls	No	No	Yes	No
Location fixed effect	No	No	No	Yes
Time fixed effects	Yes	Yes	Yes	Yes
Observations	2702	2702	2613	2702
$R^{2}$	0.095	0.110	0.136	0.174

See note to Table 3

*p<0.10, **p<0.05, ***p<0.01

## Conclusion

The pandemic has clearly affected many spheres of life. As documented in this paper, it has also affected pro-sociality. We found that participants in the experiment donated more money to a charity after receiving additional information on COVID-19, but we also documented similar positive effects of local pandemic severity and related media coverage in England. While we saw strong correlations between giving in our experiment and experiences with COVID-19, we were able to distill a pure effect of increased awareness and attention shift toward the pandemic on giving money to a charity in our experiment. Regarding experiences with COVID-19, we observed that individuals who indicated that their financial or economic situation had been negatively affected gave less. We also saw a drop in giving among those whose health had been negatively affected or who feared for their health in the future.

As we write this article, the pandemic is ongoing. Should negative economic and health consequences or fears become more pronounced or other factors change, they might outweigh the positive effect of pandemic awareness on pro-sociality as established in this paper. In the end, the results from any natural experiment have to be regarded as one snapshot in time: They are valid for the period and region under study. Nonetheless, we believe that the attention shift results are more likely to hold generally.

Further limitations of our study include the following: (i) It is difficult to assess general equilibrium effects in our experiment. We cannot say much about giving to other charities and other goals. However, based on our additional survey experiment, we found that participants in the treatment condition did not report higher urgency, effectiveness, or importance of giving to Save the Children than those in the control condition. (ii) Similar to other studies of this type, its external validity is limited. It is possible that participants increased their giving in the experiment when the costs of doing so were low but might not have changed their behavior in another context.

One of the potential directions that future research could take would be to investigate the role played by fears related to the pandemic for pro-sociality. Our data suggest substantially lower giving by those who fear negative health consequences or negative financial effects. Yet we cannot draw any causal conclusions in this respect. It seems especially challenging to come up with a potential study design that would provide causal evidence regarding pandemic-related fears, especially if researchers wish to maintain experimental standards in the field of economics.

Despite these open questions, we believe that our study makes a valuable contribution both to the field of COVID economics and to studies regarding behavior in extreme circumstances.

## Supplementary Information

Below is the link to the electronic supplementary material.

Supplementary file1 (PDF 5720 kb)

## Footnotes

1 See www.cnbc.com/2020/09/15/how-individuals-in-the-us-helped-their-neighbors-through-covid-19.html or www.abcnews.go.com/US/wireStory/volunteers-sew-masks-health-workers-facing-shortages-69764445, viewed on November 23, 2020.

2 In an additional survey experiment, we confirmed that participants did not perceive significant differences between the appeals on other dimensions.

3 A number of recent studies on charitable giving have shown that fundraisers sometimes have an inaccurate understanding of what is effective in increasing charitable giving. See for example Samek and Longfield (2019).

4 Younger participants did not change their giving significantly.

5 Further related papers include Li et al. (2020), Lotti (2020), Sheth and Wright (2020), Allen et al. (2021), Andersson et al. (2021), Arroyos-Calvera et al. (2021), Grimalda et al. (2021), Jeworrek and Waibel (2021), Kiss and Keller (2021), Mahmoud and Meyer (2021), Müller and Rau (2021), Shachat et al. (2021), Wong et al. (2021), Yue et al. (2021).

6 See Grossman and Van Der Weele (2017) for charitable giving and Delaney et al. (2014) for financial decisions under cognitive load.

7 Examples of the latter include changes in behavior due to disasters (Frey et al., 2011; Hanaoka et al., 2018; Filipski et al., 2019) or due to crises and wars (Voors et al., 2012); Adena et al., 2021.

8 There are 151 UTLAs, but COVID-19 cases were reported for two UTLAs jointly in two instances, resulting in 149 units used for the analysis.

9 These data are accessible at www.coronavirus.data.gov.uk. Alternative measures like hospitalized cases and deaths are only available at a higher level of aggregation.

10 This literature includes studies by Meer (2014), Brown et al. (2017), Whillans et al. (2017), Adena et al. (2019), Gallier et al. (2019), Alger et al. (2020), Grimson et al. (2020), Genç et al. (2021).

11 We concentrated on participants living in England and excluded participants living in Scotland, Wales and Northern Ireland because COVID-19 cases were only reported for larger geographical units in those latter territories.

12 We calibrated the fixed payment according to the time needed for the experiment and survey such that, on average, participants received at least the minimum wage. In fact, the average payment amounted to a rate of $\pounds$10.27 per hour (not accounting for what they chose to keep from the additional budget of $\pounds$1).

13 In a pilot, we tested the need for matching incentives for internal validity. We opted against using those for the following reasons: (i) We did not find significant differences in out-of-pocket giving between matching and no matching; (ii) Individuals gave at high levels without matching; (iii) Transaction costs of keeping $\pounds$1 and giving after the experiment are larger than in the experiment; (iv) Both conditions do not receive matching; (v) The majority of recent experimental papers on charitable giving in Experimental Economics did not use matching incentives.

14 The numbers of COVID-19 cases were low in the summer; we therefore paused data collection until numbers started rising again, in line with the preregistered protocol.

15 The exception here is the first session, which was conducted on both Monday and Tuesday evenings.

16 The text of the appeal and the extra paragraph were based on the information provided on the website of Save the Children UK, with some adaptations necessary for the purpose of the experiment.

17 Participants could donate any portion of the endowment. The amounts were rounded to a penny ($\pounds$0.01).

18 We deviated from the protocol in the following ways: (i) We used the share of cases up to date instead of the absolute number up to date for the reasons explained in this section, but we tested robustness to other (absolute) measures, including a new analysis based on the article count. (ii) We added the specification with local fixed effects to replace local characteristics for H2 and H4. (iii) We added a number of further robustness checks, shown in the online appendix.

19 As the slider had no natural position, the computer assigned the position for each participant at random. This might, of course, have affected the final donation decision (see Adena et al., 2017). We therefore included the initial position as a control variable.

20 The reason for adopting this approach is that our survey questions about income asked about fewer categories. Any inconsistencies between the two measures were entered into the exclusion count.

21 For detailed results, see Section F of the online appendix.

22 The source of these data is official labor market statistics (www.nomisweb.co.uk).

23 However, only the donors decided on the allocation of their own money. The non-donors decided jointly on the allocation of an additional $\pounds$100 donation.
