# Supplementary material for: COVID-19 and pro-sociality: How do donors respond to local pandemic severity, increased salience, and media coverage?
Source: Exp Econ. 2022 Apr 22;25(3):824–44. doi: 10.1007/s10683-022-09753-y (PMC9026041; doi:10.1007/s10683-022-09753-y)
Supplement: Supplementary file 1 — Supplementary file1 (PDF 5720 kb) [file 10683_2022_9753_MOESM1_ESM.pdf]

Appendix

COVID-19 and pro-sociality: How do donors respond to local pandemic severity, increased salience, and media coverage?

Maja Adena (WZB) and Julian Harke (WZB)

Contents

A Additional tables and figures **2**

B Timeline of the main experiment **14**

C Exclusion criteria **14**

D Prolific pre-screening criteria **15**

E Additional analysis **16**

F Additional survey experiment **17**
F.1 Design 17
F.2 Results 17

G Screenshots from the main experiment **20**

H Screenshots from the additional survey experiment **38**

Appendix

A Additional tables and figures

Figure A1: Control condition without COVID-19 reference

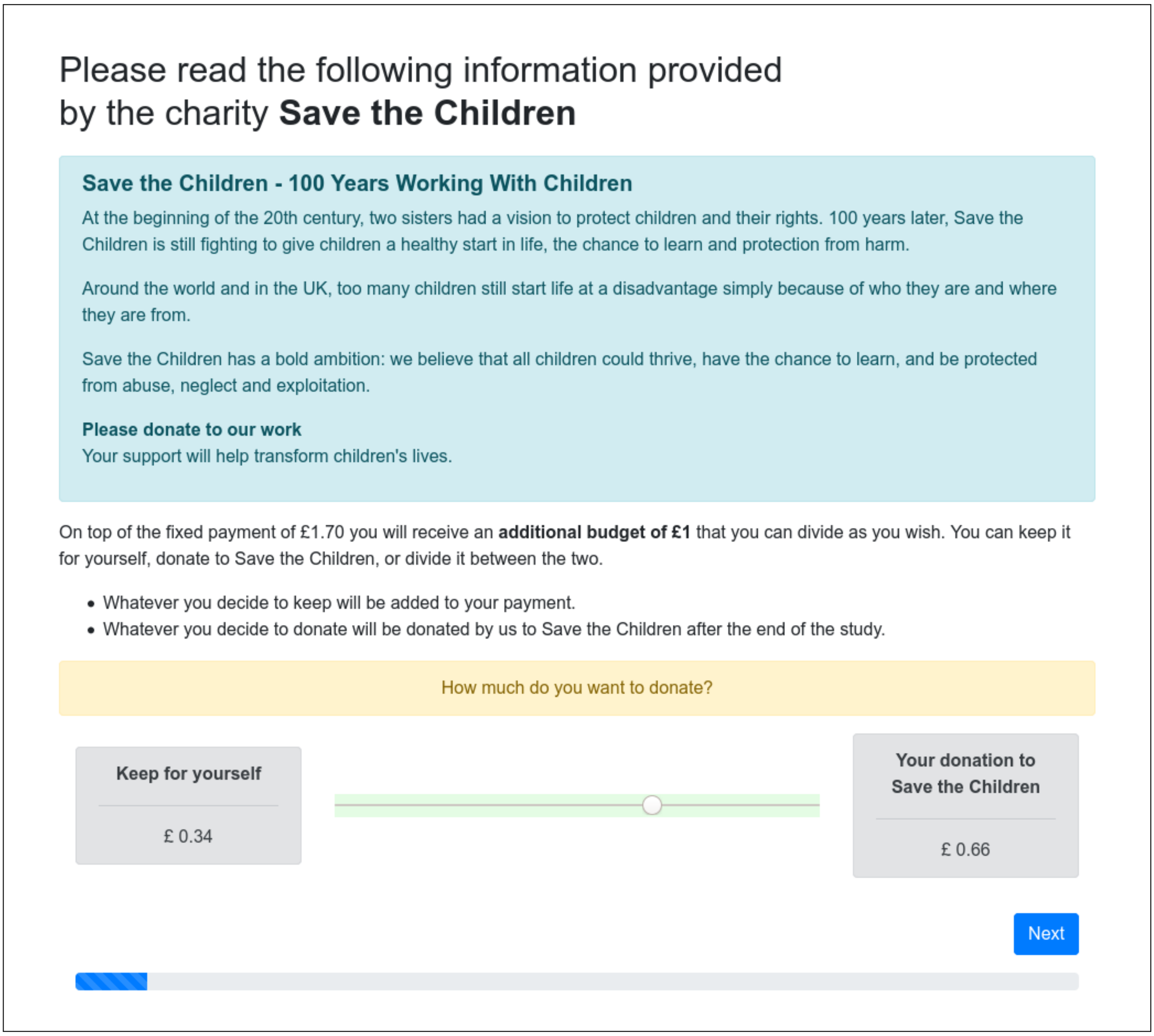

Figure A2: Treatment condition with COVID-19 reference

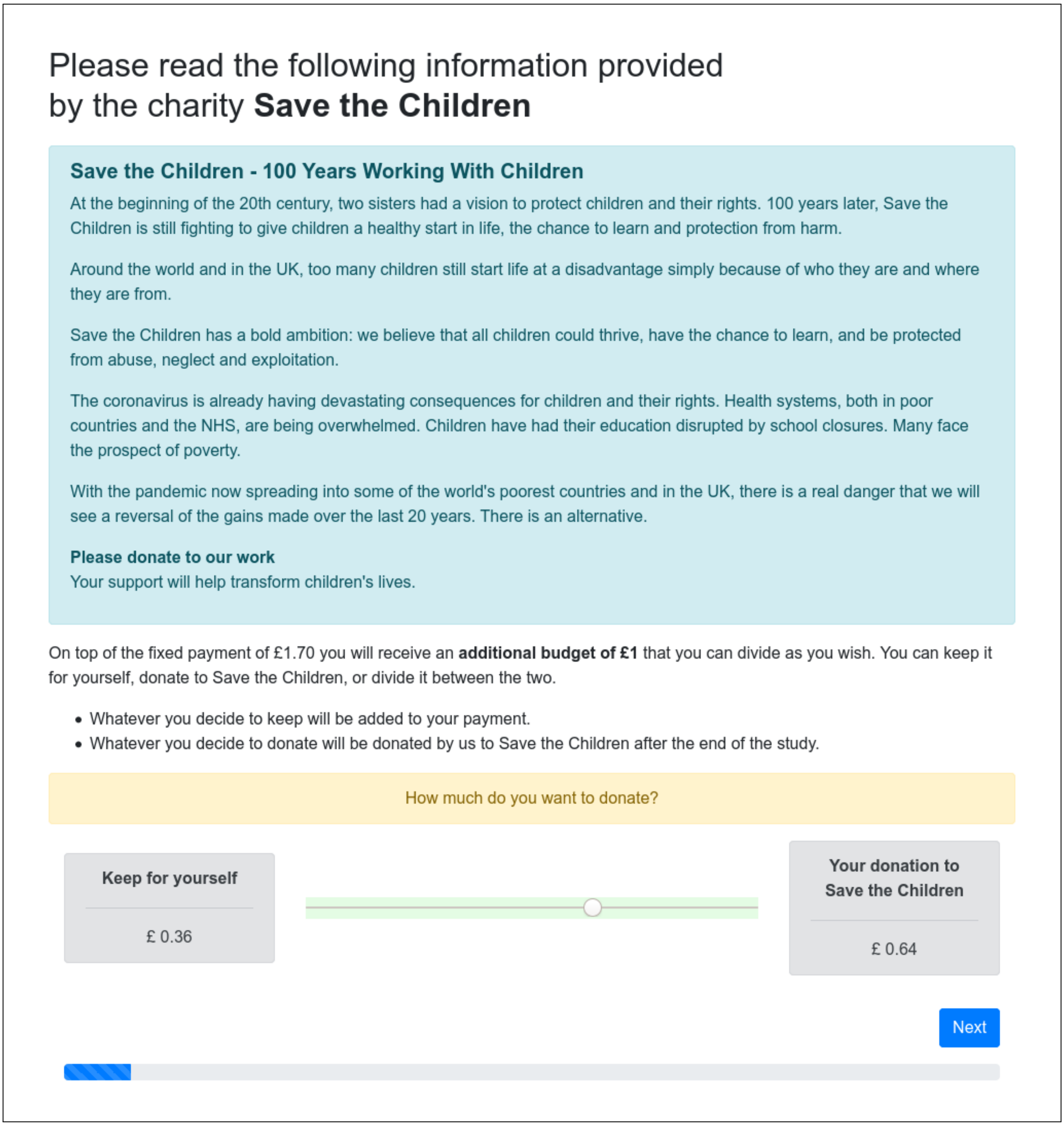
Please read the following information provided by the charity **Save the Children**

Save the Children - 100 Years Working With Children

At the beginning of the 20th century, two sisters had a vision to protect children and their rights. 100 years later, Save the Children is still fighting to give children a healthy start in life, the chance to learn and protection from harm.

Around the world and in the UK, too many children still start life at a disadvantage simply because of who they are and where they are from.

Save the Children has a bold ambition: we believe that all children could thrive, have the chance to learn, and be protected from abuse, neglect and exploitation.

The coronavirus is already having devastating consequences for children and their rights. Health systems, both in poor countries and the NHS, are being overwhelmed. Children have had their education disrupted by school closures. Many face the prospect of poverty.

With the pandemic now spreading into some of the world's poorest countries and in the UK, there is a real danger that we will see a reversal of the gains made over the last 20 years. There is an alternative.

Please donate to our work
Your support will help transform children's lives.

On top of the fixed payment of £1.70 you will receive an **additional budget of £1** that you can divide as you wish. You can keep it for yourself, donate to Save the Children, or divide it between the two.

- Whatever you decide to keep will be added to your payment.
- Whatever you decide to donate will be donated by us to Save the Children after the end of the study.

How much do you want to donate?

Keep for yourself
£ 0.36

Your donation to Save the Children
£ 0.64

Next

Figure A3: Daily COVID-19 cases in England and dates of experimental sessions

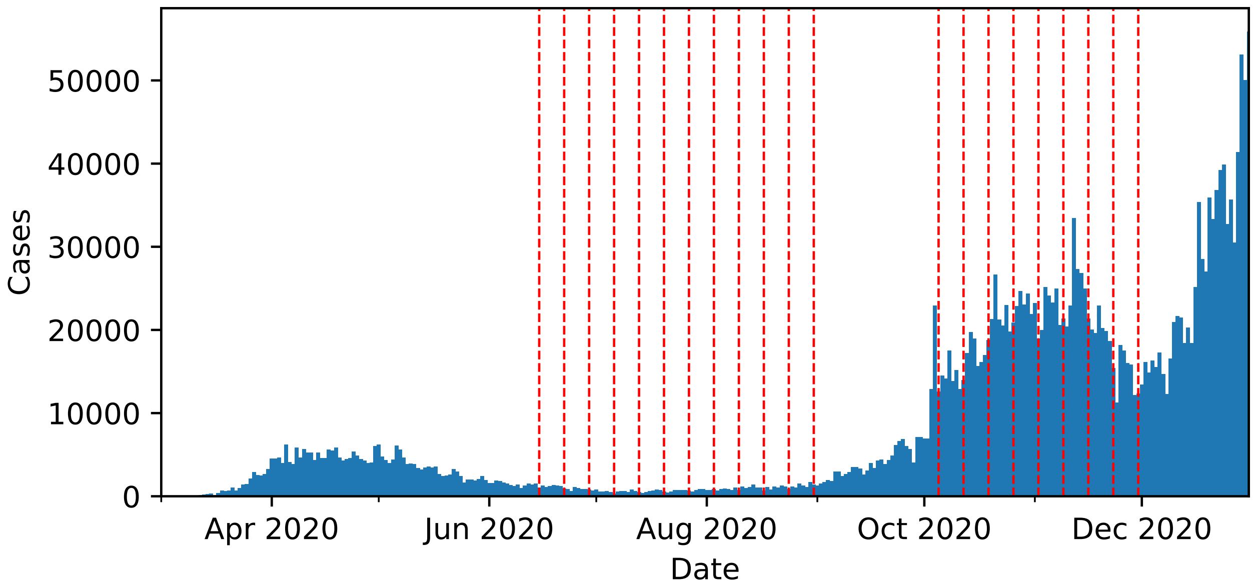

Notes: Data source coronavirus.data.gov.uk

Figure A4: Frequency of articles about the local areas related to COVID-19 in national newspapers and on BBC Online in the week preceding the experimental session

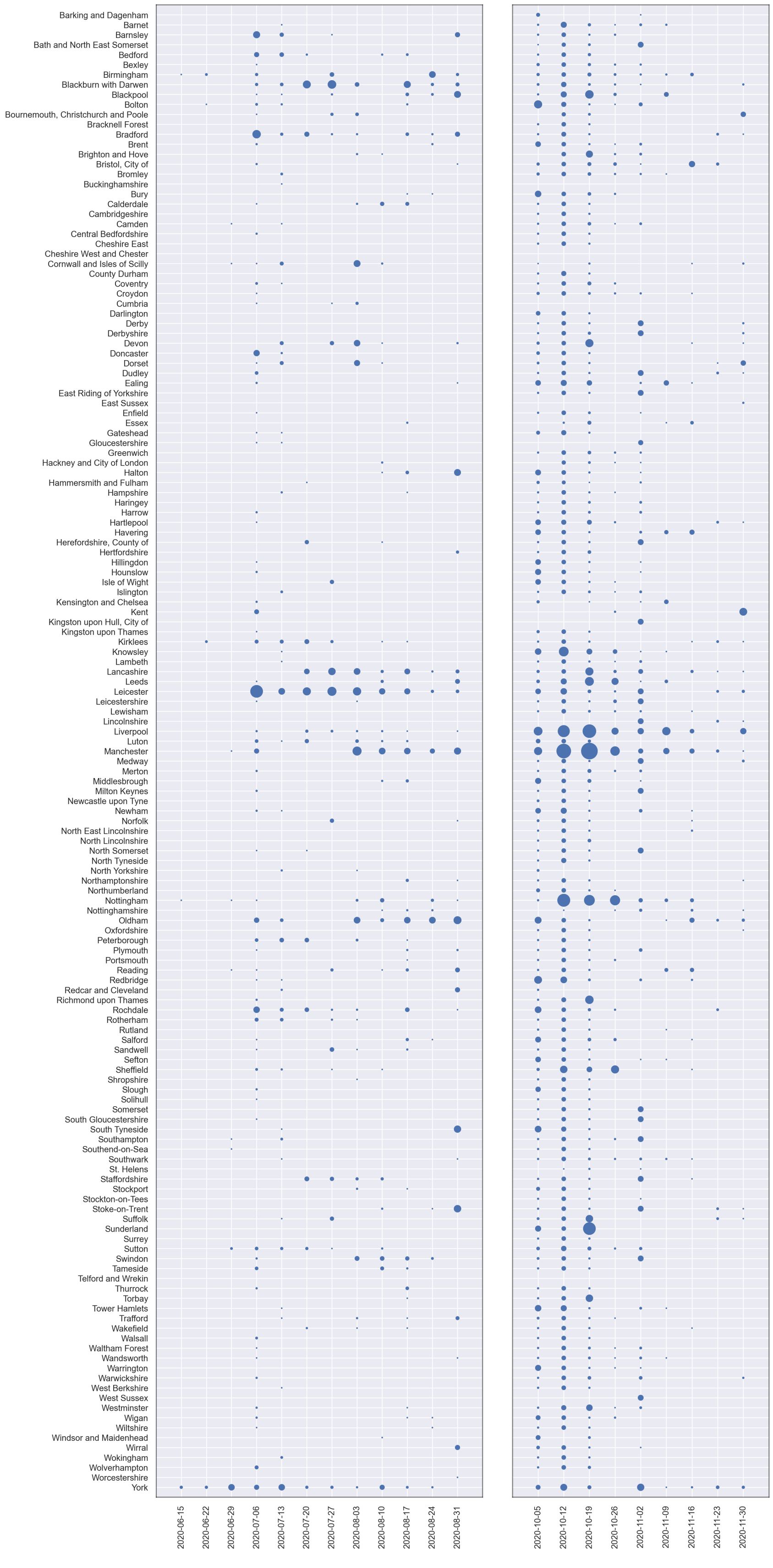

Figure A5: Examples for relative local severity and donation amounts

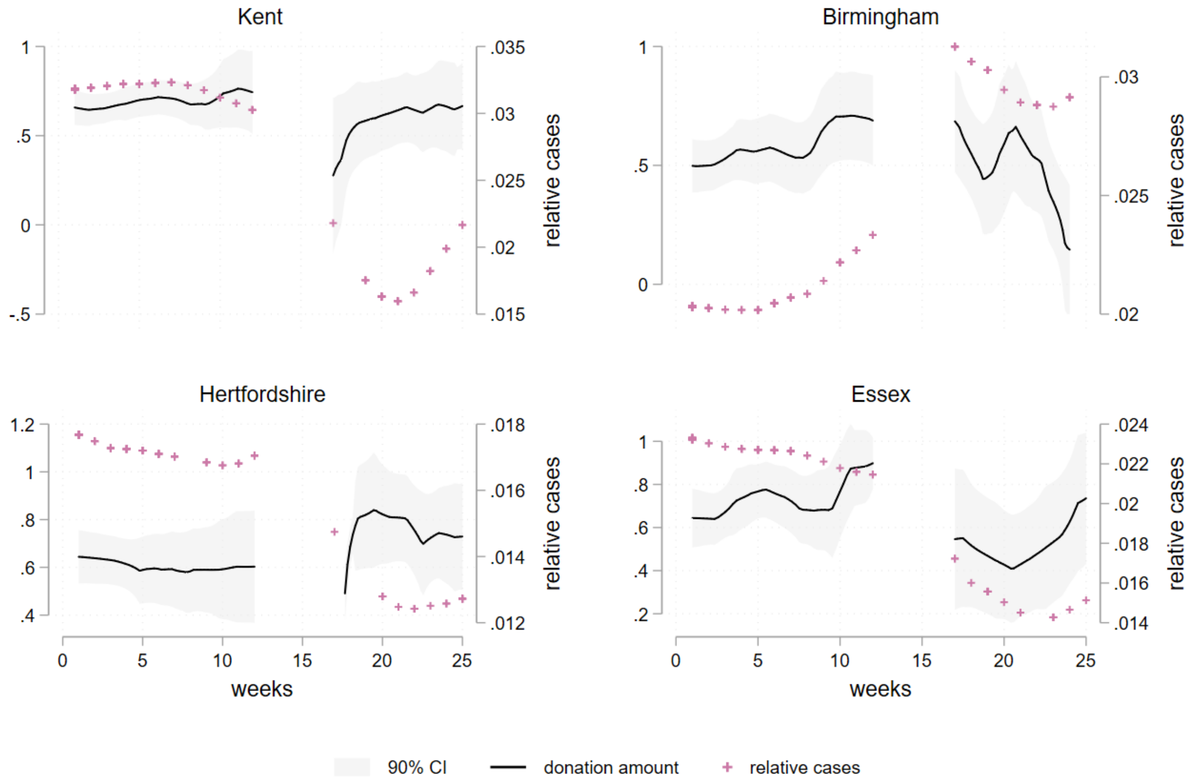

Notes: Local polynomial plot for donation amounts and 90% confidence intervals

Table A1: Descriptive statistics and balancing table

	(1) Overall			(2) By treatment						
				(a) Control			(b) COVID-19 reference			
	Mean	Std. error	N	Mean	Std. error	N	Mean	Std. error	N	T-test p-value
Relative local severity	0.008	0.000	3532	0.008	0.000	1790	0.008	0.000	1742	0.745
Initial slider: donation	0.503	0.005	3548	0.503	0.007	1799	0.503	0.007	1749	0.950
Initial slider: share UK	0.506	0.005	3548	0.510	0.007	1799	0.501	0.007	1749	0.355
Age	33.7	0.211	3547	34.0	0.296	1799	33.4	0.300	1748	0.127
Born in UK dummy	0.828	0.006	3548	0.825	0.009	1799	0.831	0.009	1749	0.643
Female dummy	0.654	0.008	3548	0.657	0.011	1799	0.652	0.011	1749	0.743
Socioeconomic status	5.325	0.025	3542	5.352	0.036	1797	5.297	0.036	1745	0.279
Household members	3.062	0.023	3548	3.084	0.032	1799	3.038	0.032	1749	0.310
Household income	3576.2	47.0	3548	3586.4	65.7	1799	3565.8	67.2	1749	0.826

Table A2: H1: The COVID-19 reference increases donations. Marginal effects after a two-limit Tobit. Outcome variable: donation amount

	(1)	(2)	(3)
COVID-19 reference	0.152*** (0.038)	0.153*** (0.038)	0.148*** (0.038)
Baseline controls	Yes	Yes	Yes
Financial controls	No	Yes	Yes
Health controls	No	No	Yes
Time fixed effects	Yes	Yes	Yes
Observations	3541	3541	3541
Pseudo R^2	0.024	0.028	0.028

Note: See note to Table 2. * $p < 0.10$, ** $p < 0.05$, *** $p < 0.01$.

Table A3: Mean donations by category

	Mean	Std. error	N
Gender:			
– female	0.640	0.3927	2322
– male	0.511	0.4213	1226
Age:			
– 18-24	0.519	0.4007	988
– 25-34	0.595	0.4079	1175
– 35-49	0.644	0.4059	901
– 50-64	0.658	0.3993	412
– 65+	0.684	0.3952	71
Making ends meet before COVID-19:			
– great difficulty	0.547	0.4140	115
– some difficulty	0.548	0.4116	847
– fairly easily	0.601	0.4047	1596
– easily	0.631	0.4037	990
Making ends meet since COVID-19:			
– great difficulty	0.523	0.4215	206
– some difficulty	0.563	0.4112	1048
– fairly easily	0.603	0.4041	1465
– easily	0.641	0.3996	829
Change in household income since COVID-19:			
– decreased a lot	0.548	0.4221	349
– decreased somewhat	0.579	0.4078	1120
– stayed the same	0.619	0.4013	1798
– increased somewhat	0.573	0.4166	248
– increased a lot	0.498	0.4284	33
Expected change in household income:			
– decrease a lot	0.540	0.4185	206
– decrease somewhat	0.599	0.4048	854
– stay the same	0.612	0.4041	1861
– increase somewhat	0.554	0.4124	576
– increase a lot	0.606	0.4323	51
Personal health or health of family negatively affected by COVID-19:			
– a lot	0.541	0.4308	146
– somewhat	0.601	0.3991	1074
– not at all	0.596	0.4096	2328
Expected negative impact on health:			
– a lot	0.484	0.4234	140
– somewhat	0.620	0.3987	2140
– not at all	0.565	0.4162	1268
Vulnerability to COVID-19:			
– high risk	0.489	0.4227	101
– moderate risk	0.612	0.4001	687
– low risk	0.595	0.4082	2760

Table A4: The effect of the relative local severity of the pandemic on subjective perceptions of local severity. Outcome variable: subjective perceptions of local severity

	(1) More severe	(2) Equally severe	(3) Less severe
Relative local severity	0.292*** (0.039)	-0.002 (0.047)	-0.290*** (0.036)
Location fixed effect	Yes	Yes	Yes
Time fixed effects	Yes	Yes	Yes
Observations	3532	3532	3532
R^2	0.218	0.108	0.286

Note: Robust errors. All columns include time fixed effects and location fixed effects. * $p < 0.10$, ** $p < 0.05$, *** $p < 0.01$.

Table A5: H2: Individuals in more affected places will give more (or less) than individuals in less affected places. Marginal effects after a two-limit Tobit.
Outcome variable: donation amount

	(1)	(2)	(3)	(4)
Relative local severity	0.063** (0.030)	0.056* (0.029)	0.157** (0.065)	0.334*** (0.103)
COVID-19 reference	0.152*** (0.038)	0.149*** (0.038)	0.147*** (0.039)	0.124*** (0.038)
Baseline controls	Yes	Yes	Yes	Yes
Financial controls	No	Yes	Yes	Yes
Health controls	No	Yes	Yes	Yes
Area controls	No	No	Yes	No
Location fixed effect	No	No	No	Yes
Time fixed effects	Yes	Yes	Yes	Yes
Observations	3525	3525	3423	3525
Pseudo R^2	0.025	0.033	0.038	0.058

Note: See note to Table 3. * $p < 0.10$, ** $p < 0.05$, *** $p < 0.01$.

Table A6: Regional trends.
Outcome variable: donation amount

	(1)
Relative local severity	0.107** (0.052)
COVID-19 reference	0.043*** (0.014)
Baseline controls	Yes
Financial controls	Yes
Health controls	Yes
Location fixed effect	Yes
Time fixed effects	Yes
Time fixed effects x Region fixed effects	Yes
Observations	3525
R^2	0.161

Note: See note to Table 3. * $p < 0.10$, ** $p < 0.05$, *** $p < 0.01$.

Table A7: Interaction effect.
Outcome variable: donation amount

	(1)	(2)	(3)	(4)
Relative local severity	0.022 (0.015)	0.018 (0.015)	0.050** (0.025)	0.104*** (0.039)
COVID-19 reference	0.051** (0.022)	0.045** (0.022)	0.043* (0.023)	0.033 (0.023)
COVID-19 reference * local severity	0.002 (0.020)	0.007 (0.020)	0.007 (0.021)	0.013 (0.021)
Baseline controls	Yes	Yes	Yes	Yes
Financial controls	No	Yes	Yes	Yes
Health controls	No	Yes	Yes	Yes
Area controls	No	No	Yes	No
Location fixed effect	No	No	No	Yes
Time fixed effects	Yes	Yes	Yes	Yes
Observations	3525	3525	3423	3525
R^2	0.052	0.069	0.080	0.118

Note: See Note to Table 3. * $p < 0.10$, ** $p < 0.05$, *** $p < 0.01$.

Table A8: H2: Individuals in more affected places will give more (or less) than individuals in less affected places. Alternative specification of local severity: absolute number of COVID-19 cases in the last 7 days (in tsd.).
Outcome variable: donation amount

	(1)	(2)	(3)	(4)
COVID-19 cases, last 7 days, in tsd.	0.028** (0.013)	0.028** (0.014)	0.031** (0.015)	0.035** (0.016)
COVID-19 reference	0.053*** (0.013)	0.051*** (0.013)	0.049*** (0.014)	0.044*** (0.014)
Baseline controls	Yes	Yes	Yes	Yes
Financial controls	No	Yes	Yes	Yes
Health controls	No	Yes	Yes	Yes
Area controls	No	No	Yes	No
Location fixed effect	No	No	No	Yes
Time fixed effects	Yes	Yes	Yes	Yes
Observations	3525	3525	3423	3525
R^2	0.052	0.068	0.079	0.116

Note: See note to Table 3. * $p < 0.10$, ** $p < 0.05$, *** $p < 0.01$.

Table A9: H2: Individuals in more affected places will give more (or less) than individuals in less affected places. Alternative specification of local severity: number of COVID-19 cases in the last 7 days per 100,000.
Outcome variable: donation amount

	(1)	(2)	(3)	(4)
COVID-19 cases,last 7 days, per 100,000	0.176 (0.112)	0.188* (0.113)	0.294** (0.125)	0.362*** (0.132)
COVID-19 reference	0.053*** (0.013)	0.051*** (0.013)	0.049*** (0.014)	0.044*** (0.014)
Baseline controls	Yes	Yes	Yes	Yes
Financial controls	No	Yes	Yes	Yes
Health controls	No	Yes	Yes	Yes
Area controls	No	No	Yes	No
Location fixed effect	No	No	No	Yes
Time fixed effects	Yes	Yes	Yes	Yes
Observations	3525	3525	3423	3525
R^2	0.051	0.069	0.080	0.117

Note: See note to Table 3. * $p < 0.10$, ** $p < 0.05$, *** $p < 0.01$.

Table A10: The effect of observables on the coefficient of interest.
Outcome variable: donation amount

	(1)	(2)	(3)	(4)	(5)	(6)
Relative local severity	0.088**	0.105***	0.108***	0.110***	0.109***	0.109***
	(0.037)	(0.037)	(0.037)	(0.037)	(0.037)	(0.037)
Baseline controls	No	Yes	Yes	Yes	Yes	Yes
Financial controls	No	No	Yes	Yes	Yes	Yes
Health controls	No	No	No	Yes	Yes	Yes
Other socioeconomic controls	No	No	No	No	Yes	Yes
Work change controls	No	No	No	No	No	Yes
Location fixed effects	Yes	Yes	Yes	Yes	Yes	Yes
Time fixed effects	Yes	Yes	Yes	Yes	Yes	Yes
Observations	3532	3525	3525	3525	3525	3525
R^2	0.062	0.101	0.109	0.118	0.123	0.124

Note: Robust errors. All columns include time fixed effects, and location fixed effects. For baseline, financial, and health controls see note to Table 2. Other socioeconomic controls include place of living dummy (big city, small city, suburbs), employement status dummy (employed, unemployed, student, apprentice, retired), number of children in the household, and primarily source of news dummy (high quality, medium quality). Work change controls include work change since COVID-19 dummies (lost permanently, lost temporarily without pay, lost temporarily with pay, hours reduced), number of days commuting before COVID-19 and since COVID-19, and remote work dummies (fully, partly). * $p < 0.10$, ** $p < 0.05$, *** $p < 0.01$.

Table A11: Correlation between economic and health variables and donation amount.
Outcome variable: donation amount

	(1)	(2)	(3)	(4)	(5)	(6)
Dummy income decreased since the outbreak of the pandemic	-0.030** (0.014)					
Dummy income expected to decrease in the next 12 months		-0.010 (0.015)				
Health negatively affected by COVID-19: a lot dummy			-0.046 (0.035)			
Expected negative impact on health: a lot dummy				-0.111*** (0.036)		
Vulnerability to COVID-19: high risk					-0.125*** (0.041)	
Vulnerability to COVID-19: moderate risk						-0.006 (0.017)
Baseline controls	Yes	Yes	Yes	Yes	Yes	Yes
Location fixed effects	Yes	Yes	Yes	Yes	Yes	Yes
Time fixed effects	Yes	Yes	Yes	Yes	Yes	Yes
Observations	3525	3525	3525	3525	3525	3525
R^2	0.100	0.099	0.100	0.102	0.102	0.099

Note: Robust errors. All columns include the following controls location fixed effects, slider initial position, age, dummy born in the UK, female dummy, socioeconomic status, number of household members, and session dummies (time fixed effects). * $p < 0.10$, ** $p < 0.05$, *** $p < 0.01$.

Table A12: H3: The national project will benefit more from the COVID-19 frame than the global project.
Outcome variable: donation share to the UK program

	(1)	(2)	(3)	(4)
COVID-19 reference	-0.004 (0.010)	-0.003 (0.010)	-0.003 (0.010)	-0.001 (0.010)
GDP in UK vs. in developing countries				-0.002 (0.001)
Poverty in UK vs. in developing countries				0.004*** (0.001)
UK more affected dummy				0.049*** (0.015)
UK equally affected dummy				0.063*** (0.015)
Baseline controls	Yes	Yes	Yes	Yes
Financial controls	No	Yes	No	Yes
Health controls	No	No	Yes	Yes
Time fixed effects	Yes	Yes	Yes	Yes
Observations	3541	3541	3541	3541
R^2	0.095	0.103	0.101	0.117

Note: See note to Table 5. The sample consists of first-stage donors and non-donors. * $p < 0.10$, ** $p < 0.05$, *** $p < 0.01$.

Table A13: H4: Individuals in more affected places will shift their giving to local causes more than those in less affected places.
Outcome variable: donation share to the UK program

	(1)	(2)	(3)	(4)
Relative local severity	0.007 (0.008)	0.007 (0.008)	-0.008 (0.017)	-0.041 (0.027)
COVID-19 reference	-0.004 (0.010)	-0.003 (0.011)	-0.003 (0.011)	-0.002 (0.011)
Baseline controls	Yes	Yes	Yes	Yes
Financial controls	No	Yes	Yes	Yes
Health controls	No	Yes	Yes	Yes
Area controls	No	No	Yes	No
Location fixed effect	No	No	No	Yes
Time fixed effects	Yes	Yes	Yes	Yes
Observations	3525	3525	3423	3525
R^2	0.094	0.108	0.132	0.164

Note: See note to Table 6. The sample consists of first-stage donors and non-donors. * $p < 0.10$, ** $p < 0.05$, *** $p < 0.01$.

B Timeline of the main experiment

After the development of the idea and programming of the experiment, we ran a technical pilot with six participants on June 5, 2020, followed by a first rough preregistration on June 8, 2020 (https://osf.io/23sc4/). This was followed by two pilots with 25 and 26 participants on the afternoons of June 8 and 10, 2020, to calibrate the payments. A final preregistration with a pre-analysis plan was completed on June 15, 2020 (https://osf.io/h5syz/). Following this, we ran several waves of the experiment on Monday evenings until the end of August 2020, starting with a larger initial sample and reducing the sample over time. Further sessions were run in October and November 2020 in order to capture the second wave of the pandemic.

C Exclusion criteria

We excluded participants who fulfilled three or more of the following criteria:

- Time taken for completing the experiment below 5 minutes or above 25 minutes,
- Estimated number of COVID-19 cases in UK below 30,000 or more than 30,000,000,
- Estimated number of cases in local area larger than the estimated number of cases in the UK/10.
- Number of household members (children plus adults) more than 8,
- Expectation that the poverty rate in the UK or in developing countries will decrease below 10%,
- Expectation that the GDP growth rate in the UK or in developing countries will increase above 10%,
- Inconsistencies between the answers reported to Prolific and answers in our survey:[1]
  - Area of residence,
  - Household income,
  - Number of household members,
  - Employment status.

[1] We did not treat those inconsistencies as direct exclusion criteria, because, for example, the inconsistencies between our survey and Prolific could have occurred due to differences in question formulation or changes to an individual's situation between Prolific's data collection and our survey.

D Prolific pre-screening criteria

Our only pre-screening criterion was the current area of residence, which needed to be in England. However, in order to secure baseline sociodemographic information, we required that the following variables have no missing values: gender, age, country of birth, household size, household income (including "prefer not to say" category for sample size reasons), and socioeconomic status, see Table D14. We chose those variables for their relevance, but excluded other variables that would result in a large reduction of the available Prolific subject pool.

Table D14: Pre-screening variables

Pre-screening	Exclusion criteria	Available subject pool size (10.06.2020)
Current UK area of residence	not England	34,650
Age	missing values	34,307
Gender	missing values and "prefer not to say"	33,921
Socioeconomic Status	missing values	32,568
Household Size	missing values	32,407
Household Income (GBP)	missing values	31,968
Country of Birth	missing values	31,902

E Additional analysis

In the preregistration, we specified a number of supporting hypotheses and tests on which we comment here. As pre-specified, we apply Bonferroni correction for multiple hypothesis testing, assuming 20 tests. In the following, barely any test is confirmed. Many of the tests concern, however, the outcome being the share of donations to the UK program and interactions with the treatment dummy for which the direct effect has been shown not to be significant in the main analysis. The score variables were created following a preregistered protocol.

SH0 Interaction effects of the two main explanatory variables: The coefficient on the interaction between treatment dummy and local severity is not significant (and very small) (see Table A7 in the main article).

SH1 COVID-19 skeptics will decrease giving in the treatment condition: The interaction term (as well as the direct coefficient on COVID-19 skeptics score) is not significant.

SH2 Those who follow rules and recommendations regarding COVID-19 will increase their giving in the treatment condition: The interaction term (as well as the direct coefficient on rule followers score) is not significant.

SH3a-c Regarding the impact of reporting in the media on giving to the local program versus the global program, there was not enough variation across sessions to test those hypotheses.

SH4a-c The relative amount of giving to the UK program versus the global program will reflect the perception of how negatively the UK will be impacted relative to developing countries. In a regression analysis, the following explanatory variables are looked at: GDP growth in the UK versus developing countries, poverty in the UK versus in developing countries, dummy UK more affected by COVID-19 (subjective statement), and the interactions with the treatment. For the direct effects, see Table 5 and description in the main text. Regarding the interaction effects, only the coefficient on the interaction between the dummy UK more affected by COVID-19 and the treatment is positive and significant.

SH5
- a Individuals whose economic situations have been negatively affected since the spread of COVID-19 and those fearing such negative consequences will donate less than others: We confirm this hypothesis.
- b Individuals whose health status has been negatively affected since the spread of COVID-19 and those fearing health deterioration will donate less than others. The coefficient on the health score is not significant (the reason is likely an inverted u-shaped pattern of giving in health, on which we comment in the main text and which seems to not be well reflected in the created health score variable).
- c Individuals with less distancing opportunities will donate less than others. The coefficient on the distancing score is not significant.

SH-Other Individuals might donate less in the treatment condition if they think that they have contributed sufficiently to prevention and mitigation of the consequences of COVID-19: Coefficient is not significant.

SH-Other COVID-19 individual contribution and level of empathy: We confirm a positive correlation between empathy and giving in the experiment.

F Additional survey experiment

F.1 Design

We designed an additional survey experiment to better understand the mechanism behind the results of our main experiment, where we found higher giving in the treatment group compared to the control group. In addition, the survey aimed at informing us about a potential experimenter demand effect arising in the main experiment. Following the design of the original experiment, we recruited 220 participants on Prolific who indicated their area of residence to be in England. We used the same pre-screening (see Section D) and exclusion (see Section C) criteria as for the main experiment. The latter resulted in the final sample of 172 participants used in the analysis. The survey was not incentivized, and the participants received a fixed amount of £2 after the completion of the survey. Similar to the main experiment, in the control group, the participants read a donation ask for Save the Children. In the treatment group, the participants read the same donation ask with the additional paragraph about COVID-19. Next, on each page, participants were asked to “think of an average Prolific participant from the UK who might receive this donation appeal” and answered a number of questions regarding how they think the donation appeal would affect such a person. The additional survey experiment was preregistered on OSF (https://osf.io/rw86z/) prior to the experimental sessions at the end of April, 2021. The preregistration contains further details of the survey experiment, the hypotheses, and screenshots of the experimental instructions.

F.2 Results

Next, we show the results of various tests of differences between treatments. First, we asked participants to answer how strongly they expected the appeal to evoke different emotions in the average Prolific participant. We asked the question separately for all 20 emotions that are part of the Geneva Emotional Wheel (GEW, see Scherer, 2005; Scherer et al., 2013). We took the average over the positive and over the negative emotions. Both variables range from 0 to 100. Table F1 shows results from OLS regressions. We see that the treatment evokes less positive emotions (marginally significant at $p<0.1$)[2] and more negative emotions (not significant).

Table F1: AddH1a/b: The COVID-19 reference evokes more positive/negative emotions in the reader.

Outcome variable	Positive emotions	Negative emotions
COVID-19 reference	-5.586* (2.917)	3.177 (2.487)
Baseline controls	Yes	Yes
Observations	172	172
R^2	0.053	0.029

Note: OLS regressions; robust errors. Baseline controls include age, UK birth dummy, female dummy, socioeconomic status dummies, and household size. * $p < 0.10$, ** $p < 0.05$, *** $p < 0.01$.

Next, we tested, whether participants expect the money to be spent sooner in the treatment condition. Table F2 presents the results which show no significant differences in the expected timing of relief in both treatments.

Table F2: AddH2: Participants expect their donation to be spent sooner in the treatment with COVID-19 reference.

Outcome variable	Time money spent
COVID-19 reference	-0.146 (0.111)
Baseline controls	Yes
Observations	172
R^2	0.055

Note: See note to Table F1. * $p < 0.10$, ** $p < 0.05$, *** $p < 0.01$.

[2] In the text and in the tables, we do not correct for multiple hypothesis testing. However, Bonferroni corrections are easily implemented if we multiply the p-value by the number of tests (9). The difference in positive emotions does not survive such comparisons, and the only test that survives is AddH3.

We asked participants to name the goals that they expected the donations collected in the appeal to be spent on. They entered text into an open text field. We opted against providing a multiple-choice list as this could have influenced their responses. We classified the words used in the responses into major categories including COVID-19 (participants having included words such as pandemic, corona, or coronavirus). While in the control treatment, no one mentioned COVID-19, 16% in the treatment condition did so, and the difference is statistically significant, as can be seen in Table F3. However, this compares to, altogether, 51% mentioning education, 38% protection, 29% health, 22% support, 16% poverty, and 13% hunger.[3] This means that COVID-19 relief was not perceived as the main goal of the project.

Table F3: AddH3: Participants expect the money to be (more often) spent on COVID-19 relief in the treatment with COVID-19 reference.

Outcome variable	COVID-19 relief
COVID-19 reference	0.158*** (0.038)
Baseline controls	Yes
Observations	172
R^2	0.110

Note: See note to Table F1. * $p < 0.10$, ** $p < 0.05$, *** $p < 0.01$.

Next, we asked participants to compare the perceived importance, effectiveness, and urgency of the donation to Save the Children's appeal with a donation to (i) Transparency International, (ii) the World Wildlife Fund, and (iii) the Alzheimer's Society. The participants answered by using a slider on a scale from less important/effective/urgent to more important/effective/urgent. For each participant, we computed an average over the three charities and used this score for the final comparisons. The score ranges from 0 to 100. The results are presented in Table F4. There are no significant differences in how important, effective, or urgent participants perceive giving to Save the Children in the treatment versus the control condition.

Table F4: AddH4–6: In the treatment with COVID-19 reference, giving to the appeal is perceived to be more effective/important/urgent.

Outcome variable	Relative effectiveness	Relative importancy	Relative urgency
COVID-19 reference	-0.032 (2.003)	0.699 (1.875)	1.671 (2.011)
Baseline controls	Yes	Yes	Yes
Observations	172	172	172
R^2	0.012	0.019	0.024

Note: See note to Table F1. * $p < 0.10$, ** $p < 0.05$, *** $p < 0.01$.

Next, we studied whether the treatment condition might exert on participants more pressure to give. In the literature, it has been long recognized that more (social) pressure results in higher giving (see, among others, Andreoni et al., 2017; DellaVigna et al., 2012). Moreover, anecdotal evidence suggests that fundraisers actively use such techniques. We asked a randomly chosen 50% of our sample (equally distributed among the treatments) to judge the following statement: "The person would feel pressure to donate when receiving such a donation request in a letter by the Royal Mail." Participants answered by using a slider on a scale from "not at all" to "a lot," coded 0–100. The results in Table F5 show that the difference is not significant.

Table F5: H7: The COVID-19 reference results in more pressure to donate.

Outcome variable	Pressure to donate
COVID-19 reference	1.349 (5.822)
Baseline controls	Yes
Observations	81
R^2	0.057

Note: See note to Table F1. * $p < 0.10$, ** $p < 0.05$, *** $p < 0.01$.

[3]This sums to more than 100% since participants were allowed to name multiple goals.

Next, we checked for a potential unintended experimenter demand effect in our main experiment. We asked the remaining 50% of the sample the following question: "The person would feel pressure to donate when receiving such a donation request in a study on Prolific." The participants answer using a slider on a scale from "not at all" to "a lot." Using a difference-in-difference approach, we study whether the additional pressure in the treatment condition in our experiment is different from that which the participants believe to experience in real life. Table F6 shows coefficients on treatment, dummy for the group that judged the pressure to give on Prolific (versus real life), and the interaction between the two. The coefficient on the interaction term is not significant, meaning that the pressure in the treatment condition is not different from that which would arise in real life (level coefficients are also not significant). In Figure F1, we present the averages in perceived pressure by treatments.

Table F6: H8: The participants in our initial experiment on Prolific feel higher pressure to donate in the treatment with COVID-19 reference that one would expect to arise in real life.

Outcome variable	Pressure to donate
COVID-19 reference x Prolific	-3.062 (7.727)
COVID-19 reference	5.129 (5.337)
Prolific	4.368 (5.244)
Baseline controls	Yes
Observations	172
R^2	0.037

Note: See note to Table F1. * $p < 0.10$, ** $p < 0.05$, *** $p < 0.01$.

Figure F1: Perceived pressure to donate in the experiment and in real life, by treatment condition

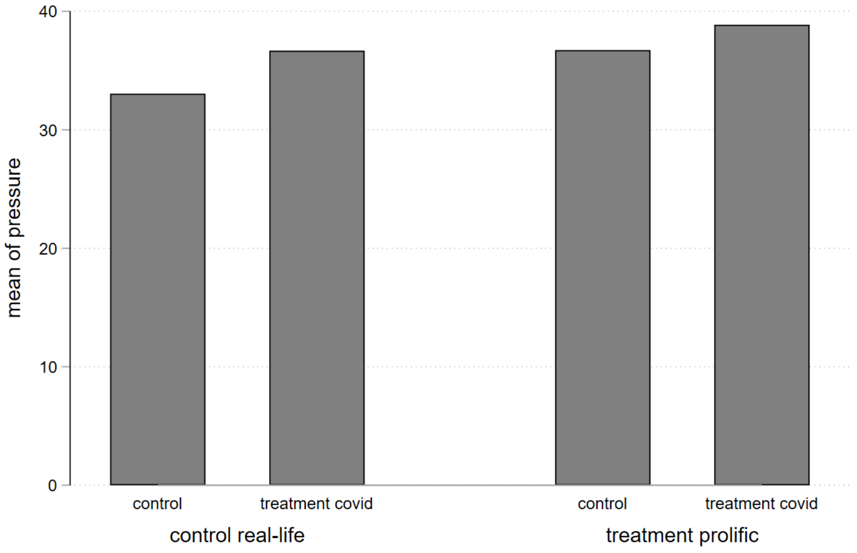

Note: Own data.

G Screenshots from the main experiment

Figure G2: Introduction

Introduction

Thank you for signing up. This study consists of two parts. In the first part, we ask you to make two decisions. In the second part, you are supposed to answer some survey questions.

This research is very important to us. We therefore ask you to pay attention and try to answer as precise as you can. Thank you!

Payment
If you complete both parts of the study, you will be paid at least £1.70.

The maximum time allowed to complete this study is 49 minutes. You will not be entitled to any payment if you exceed the maximum time allowed.

Data protection information

Hide

The Berlin Social Science Center (Wissenschaftszentrum Berlin für Sozialforschung, WZB), Reichpietschufer 50, 10785 Berlin, Germany is conducting a scientific study using Prolific today.

Your responses will be recorded on our server. The data generated in the study will be separated from the data in the Prolific system after the payment has been completed and will not allow any inference on the participation respectively the responses of individual persons. Correspondingly, the analysis and presentation of all results of this survey will be anonymized. The anonymous research data will be archived and will possibly be made available to other scientists for further use.

Participation in today's study is entirely voluntary. You have the possibility to exit the study at any time.

Next

Figure G3: Control condition without COVID-19 reference

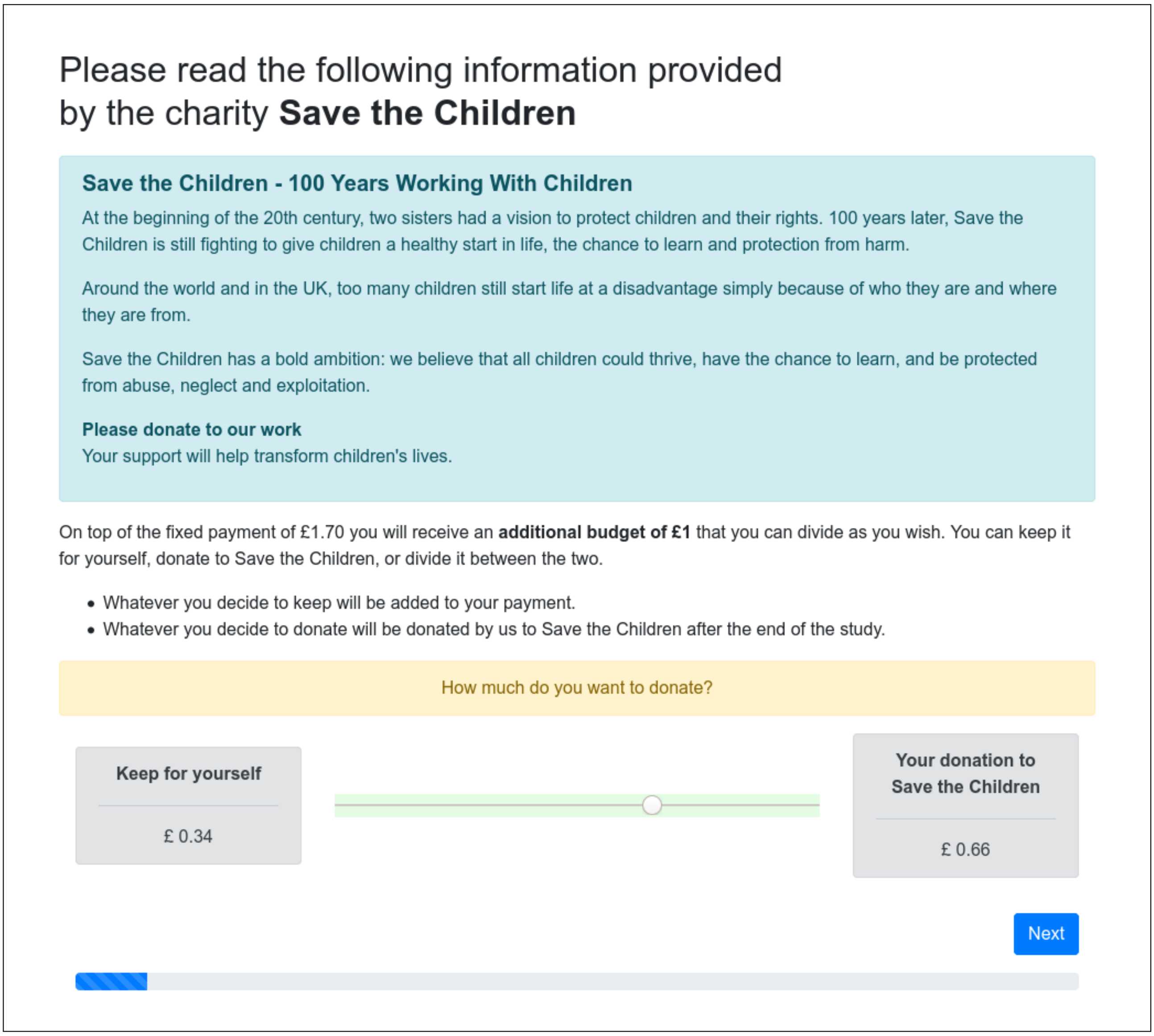

Figure G4: Treatment condition with COVID-19 reference

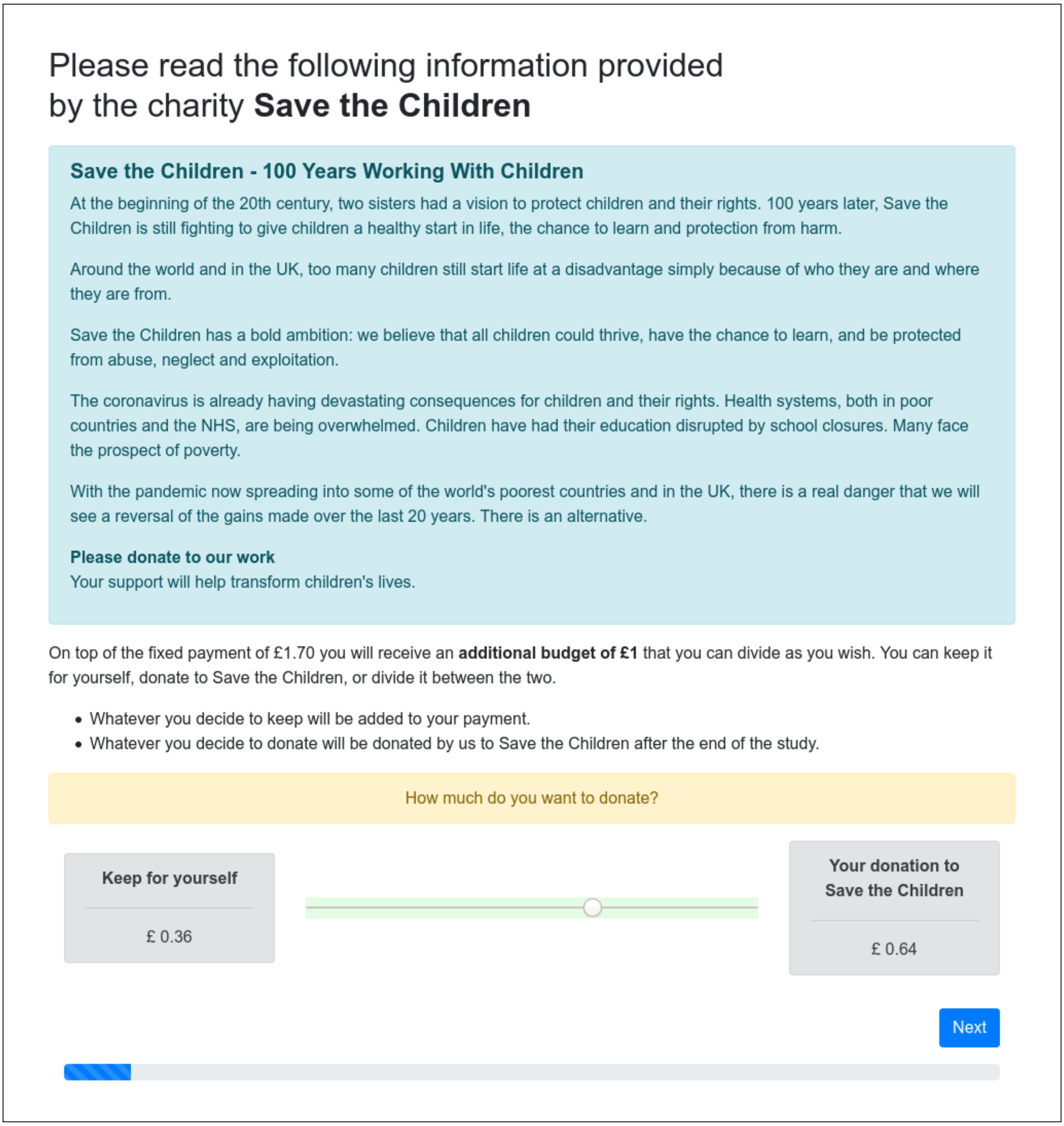

Please read the following information provided by the charity **Save the Children**

Save the Children - 100 Years Working With Children

At the beginning of the 20th century, two sisters had a vision to protect children and their rights. 100 years later, Save the Children is still fighting to give children a healthy start in life, the chance to learn and protection from harm.

Around the world and in the UK, too many children still start life at a disadvantage simply because of who they are and where they are from.

Save the Children has a bold ambition: we believe that all children could thrive, have the chance to learn, and be protected from abuse, neglect and exploitation.

The coronavirus is already having devastating consequences for children and their rights. Health systems, both in poor countries and the NHS, are being overwhelmed. Children have had their education disrupted by school closures. Many face the prospect of poverty.

With the pandemic now spreading into some of the world's poorest countries and in the UK, there is a real danger that we will see a reversal of the gains made over the last 20 years. There is an alternative.

Please donate to our work
Your support will help transform children's lives.

On top of the fixed payment of £1.70 you will receive an **additional budget of £1** that you can divide as you wish. You can keep it for yourself, donate to Save the Children, or divide it between the two.

- Whatever you decide to keep will be added to your payment.
- Whatever you decide to donate will be donated by us to Save the Children after the end of the study.

How much do you want to donate?

Keep for yourself

£ 0.36

Your donation to Save the Children

£ 0.64

Next

Figure G5: Second decision for donors

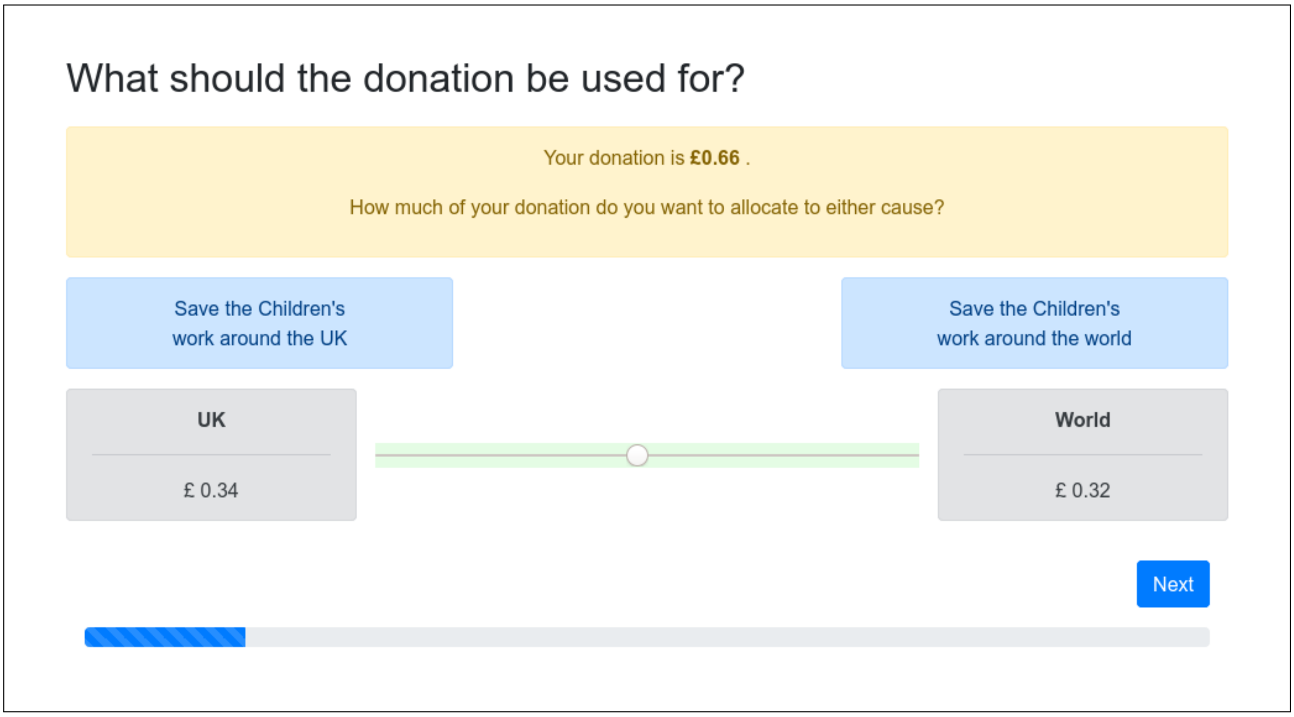

Figure G6: Second decision for non-donors

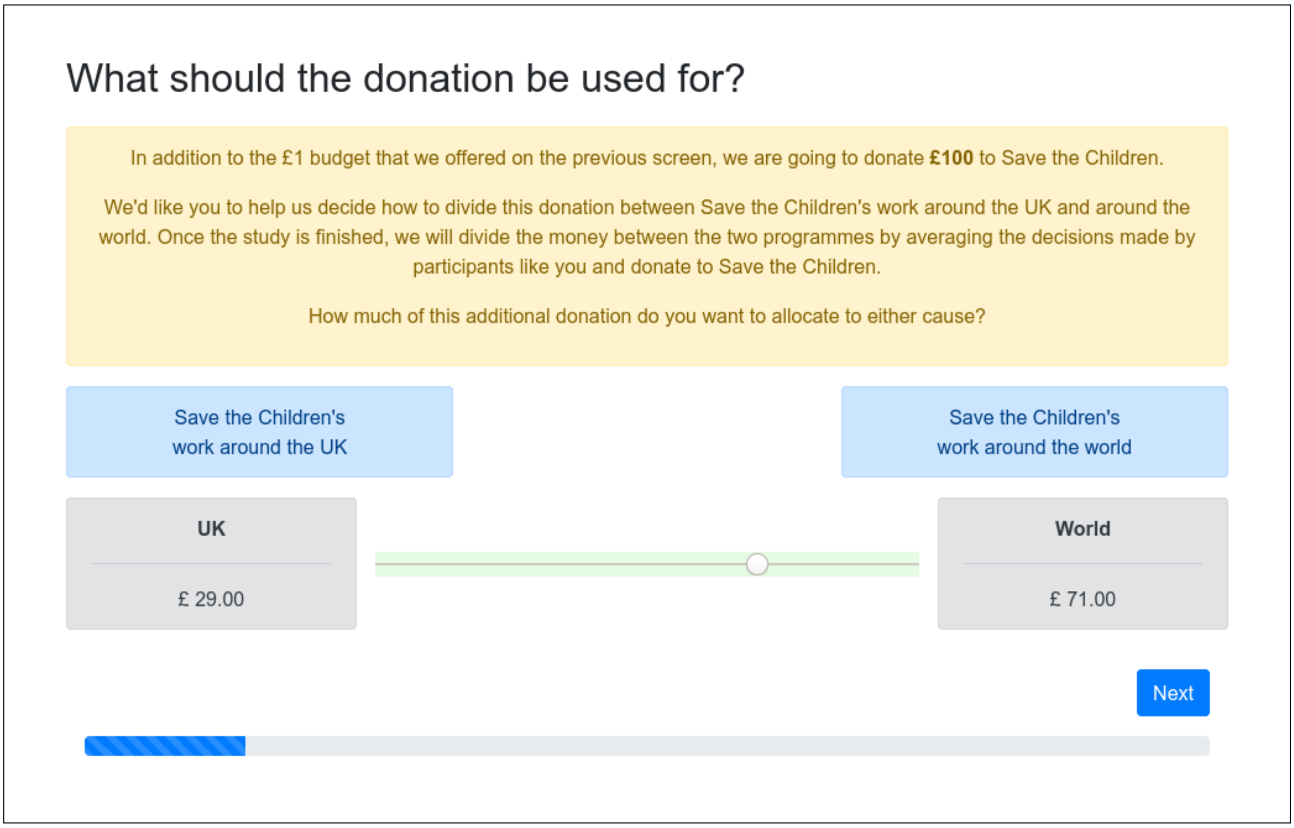

Figure G7: Introduction to survey

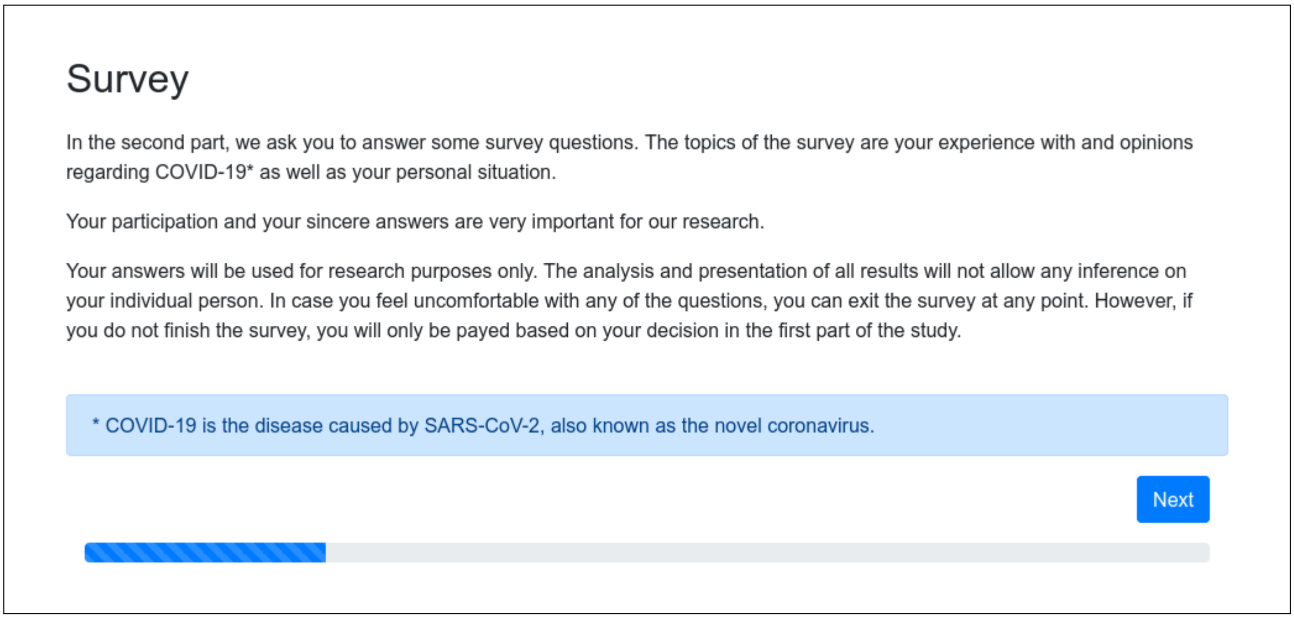

Survey

In the second part, we ask you to answer some survey questions. The topics of the survey are your experience with and opinions regarding COVID-19* as well as your personal situation.

Your participation and your sincere answers are very important for our research.

Your answers will be used for research purposes only. The analysis and presentation of all results will not allow any inference on your individual person. In case you feel uncomfortable with any of the questions, you can exit the survey at any point. However, if you do not finish the survey, you will only be payed based on your decision in the first part of the study.

* COVID-19 is the disease caused by SARS-CoV-2, also known as the novel coronavirus.

Next

Figure G8: Demographics

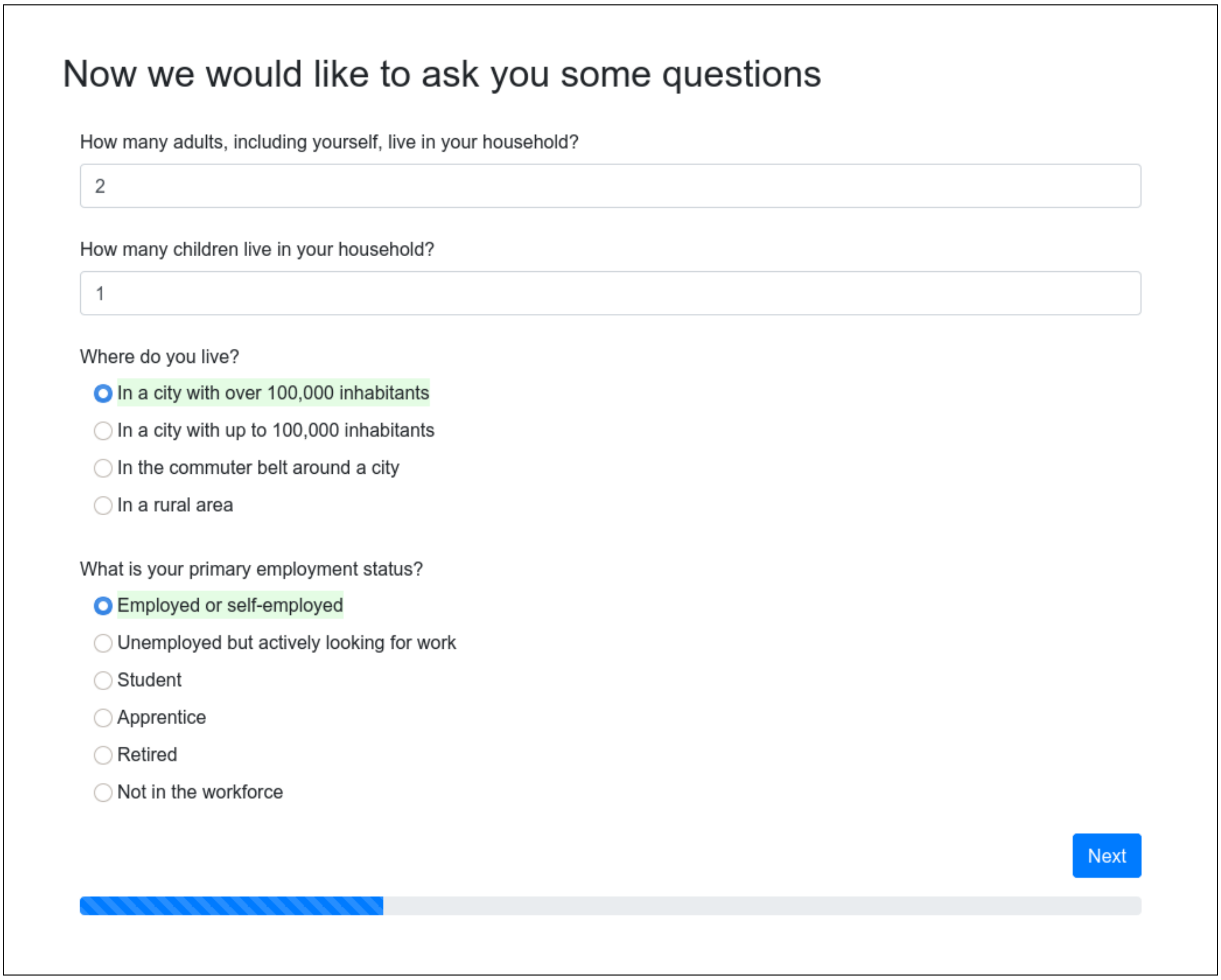

Now we would like to ask you some questions

How many adults, including yourself, live in your household?

2

How many children live in your household?

1

Where do you live?

- (•) In a city with over 100,000 inhabitants
- ( ) In a city with up to 100,000 inhabitants
- ( ) In the commuter belt around a city
- ( ) In a rural area

What is your primary employment status?

- (•) Employed or self-employed
- ( ) Unemployed but actively looking for work
- ( ) Student
- ( ) Apprentice
- ( ) Retired
- ( ) Not in the workforce

Next

Figure G9: Location selection

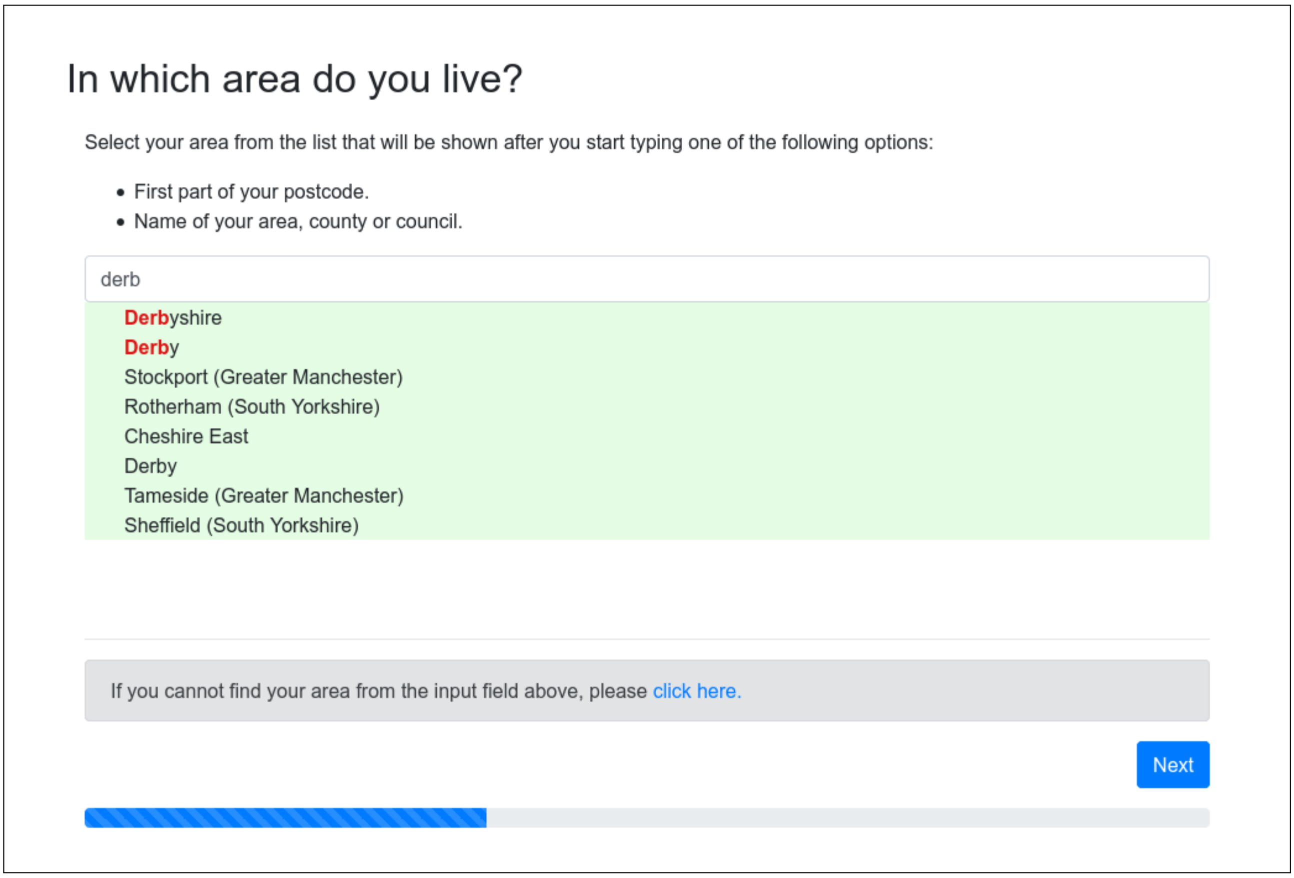

Figure G10: Location confirmation

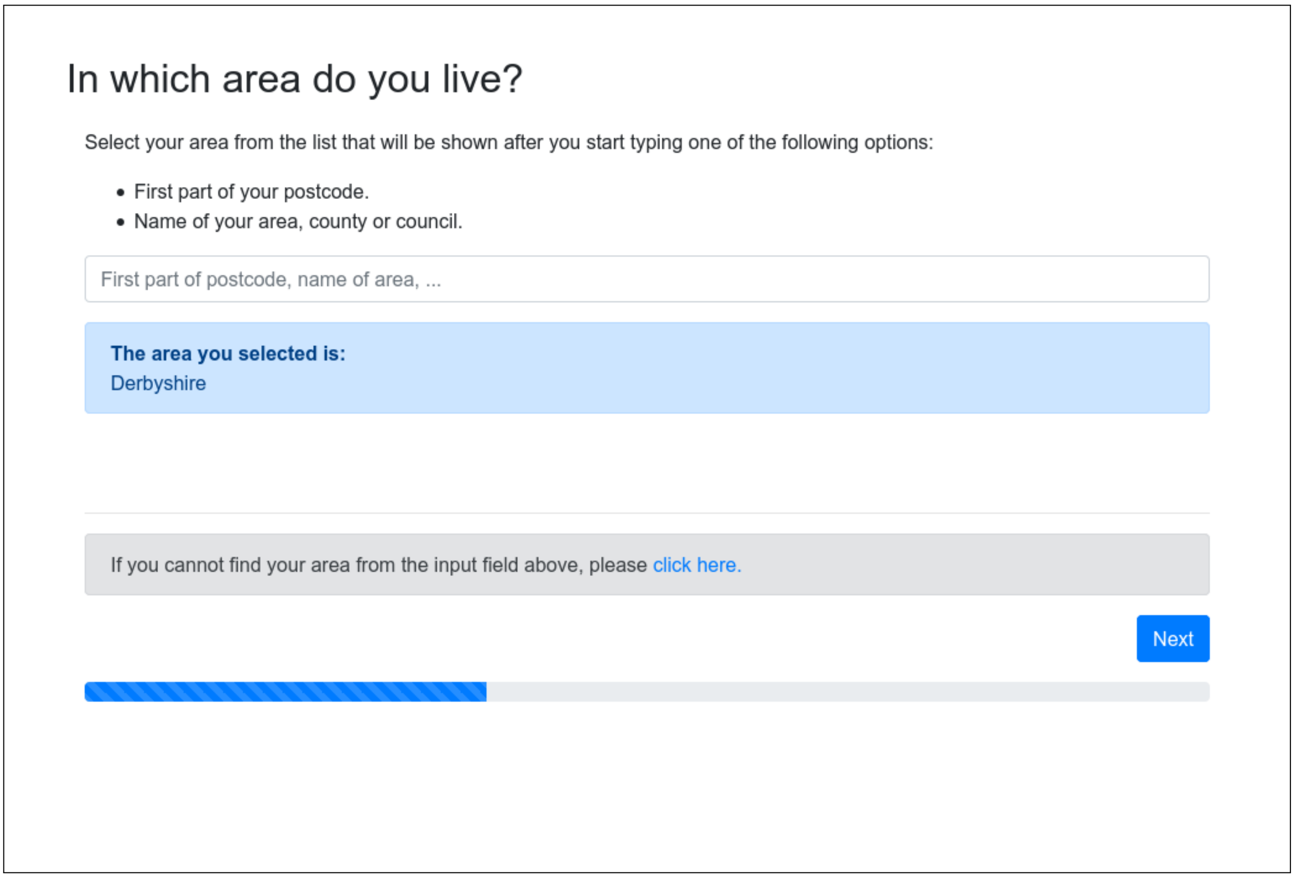

Figure G11: Experiences with COVID-19

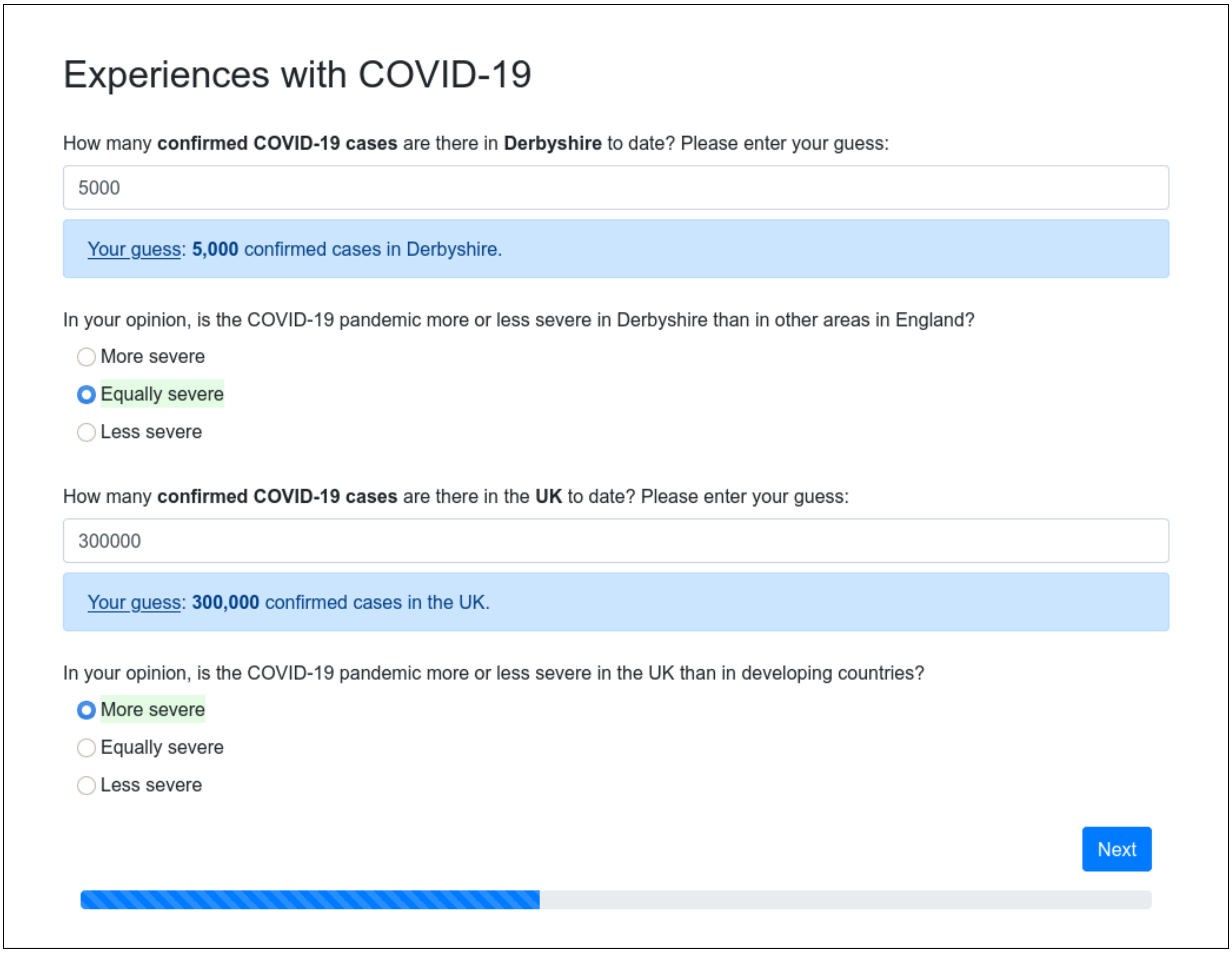

Experiences with COVID-19

How many **confirmed COVID-19 cases** are there in **Derbyshire** to date? Please enter your guess:

5000

Your guess: **5,000** confirmed cases in Derbyshire.

In your opinion, is the COVID-19 pandemic more or less severe in Derbyshire than in other areas in England?

- More severe
- Equally severe
- Less severe

How many **confirmed COVID-19 cases** are there in the **UK** to date? Please enter your guess:

300000

Your guess: **300,000** confirmed cases in the UK.

In your opinion, is the COVID-19 pandemic more or less severe in the UK than in developing countries?

- More severe
- Equally severe
- Less severe

Next

Figure G12: Health situation

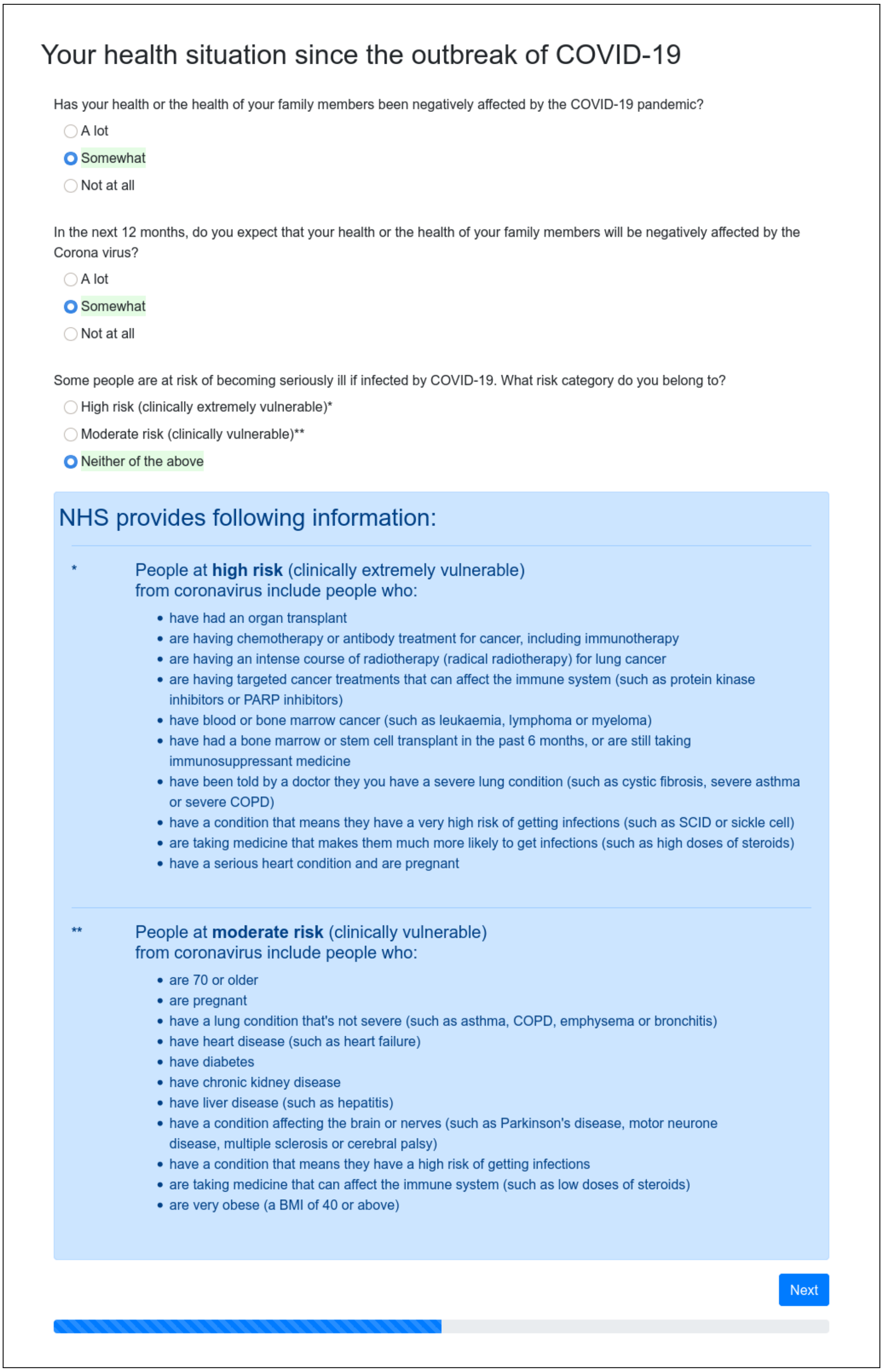

Your health situation since the outbreak of COVID-19

Has your health or the health of your family members been negatively affected by the COVID-19 pandemic?

- ( ) A lot
- (•) Somewhat
- ( ) Not at all

In the next 12 months, do you expect that your health or the health of your family members will be negatively affected by the Corona virus?

- ( ) A lot
- (•) Somewhat
- ( ) Not at all

Some people are at risk of becoming seriously ill if infected by COVID-19. What risk category do you belong to?

- ( ) High risk (clinically extremely vulnerable)*
- ( ) Moderate risk (clinically vulnerable)**
- (•) Neither of the above

NHS provides following information:

* People at **high risk** (clinically extremely vulnerable) from coronavirus include people who:

- have had an organ transplant
- are having chemotherapy or antibody treatment for cancer, including immunotherapy
- are having an intense course of radiotherapy (radical radiotherapy) for lung cancer
- are having targeted cancer treatments that can affect the immune system (such as protein kinase inhibitors or PARP inhibitors)
- have blood or bone marrow cancer (such as leukaemia, lymphoma or myeloma)
- have had a bone marrow or stem cell transplant in the past 6 months, or are still taking immunosuppressant medicine
- have been told by a doctor they you have a severe lung condition (such as cystic fibrosis, severe asthma or severe COPD)
- have a condition that means they have a very high risk of getting infections (such as SCID or sickle cell)
- are taking medicine that makes them much more likely to get infections (such as high doses of steroids)
- have a serious heart condition and are pregnant

** People at **moderate risk** (clinically vulnerable) from coronavirus include people who:

- are 70 or older
- are pregnant
- have a lung condition that's not severe (such as asthma, COPD, emphysema or bronchitis)
- have heart disease (such as heart failure)
- have diabetes
- have chronic kidney disease
- have liver disease (such as hepatitis)
- have a condition affecting the brain or nerves (such as Parkinson's disease, motor neurone disease, multiple sclerosis or cerebral palsy)
- have a condition that means they have a high risk of getting infections
- are taking medicine that can affect the immune system (such as low doses of steroids)
- are very obese (a BMI of 40 or above)

Next

Figure G13: Behavior since the outbreak of COVID-19

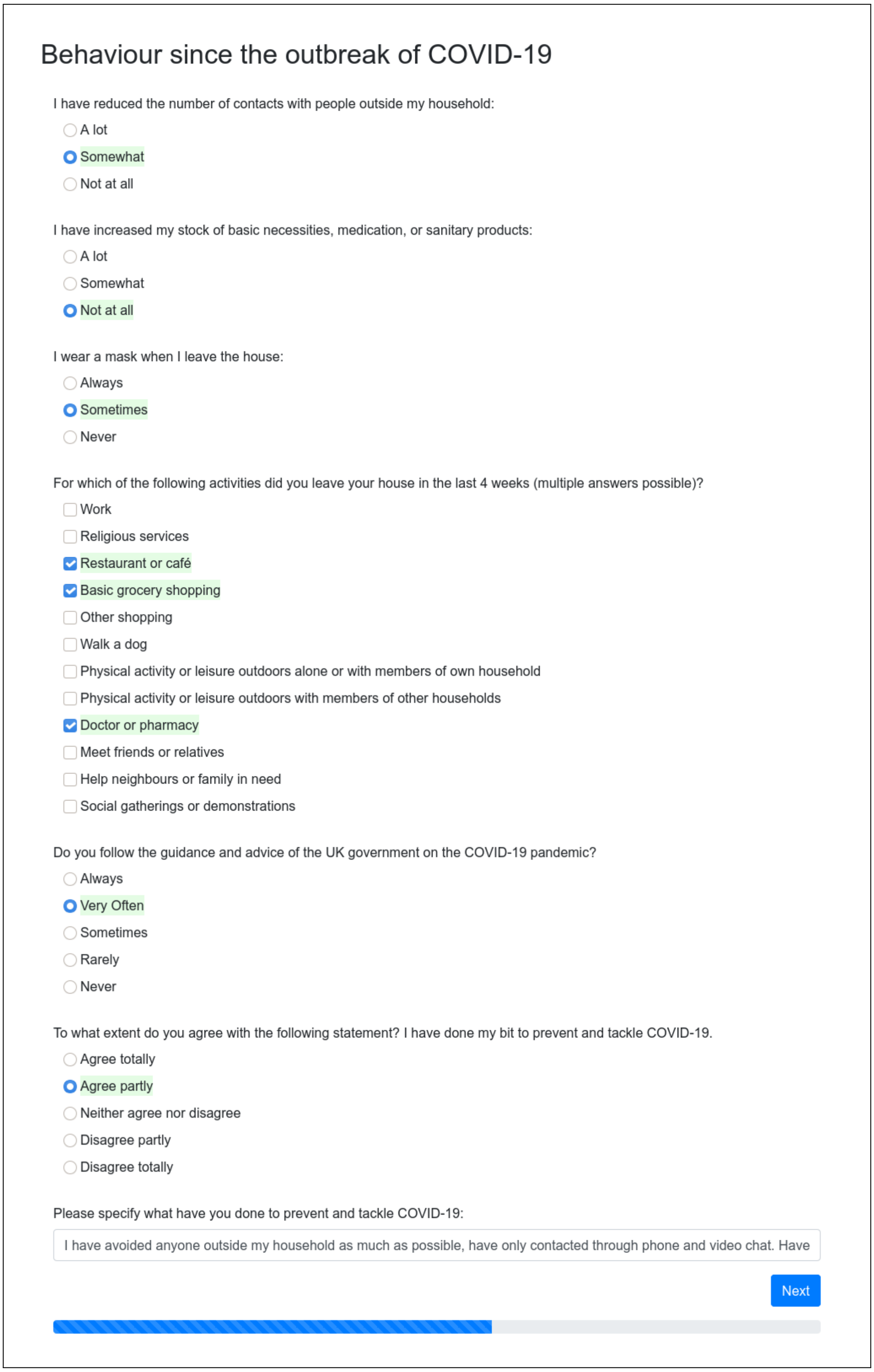

Behaviour since the outbreak of COVID-19

I have reduced the number of contacts with people outside my household:

- A lot
- Somewhat
- Not at all

I have increased my stock of basic necessities, medication, or sanitary products:

- A lot
- Somewhat
- Not at all

I wear a mask when I leave the house:

- Always
- Sometimes
- Never

For which of the following activities did you leave your house in the last 4 weeks (multiple answers possible)?

- Work
- Religious services
- Restaurant or café
- Basic grocery shopping
- Other shopping
- Walk a dog
- Physical activity or leisure outdoors alone or with members of own household
- Physical activity or leisure outdoors with members of other households
- Doctor or pharmacy
- Meet friends or relatives
- Help neighbours or family in need
- Social gatherings or demonstrations

Do you follow the guidance and advice of the UK government on the COVID-19 pandemic?

- Always
- Very Often
- Sometimes
- Rarely
- Never

To what extent do you agree with the following statement? I have done my bit to prevent and tackle COVID-19.

- Agree totally
- Agree partly
- Neither agree nor disagree
- Disagree partly
- Disagree totally

Please specify what have you done to prevent and tackle COVID-19:

I have avoided anyone outside my household as much as possible, have only contacted through phone and video chat. Have

Next

Figure G14: Work situation

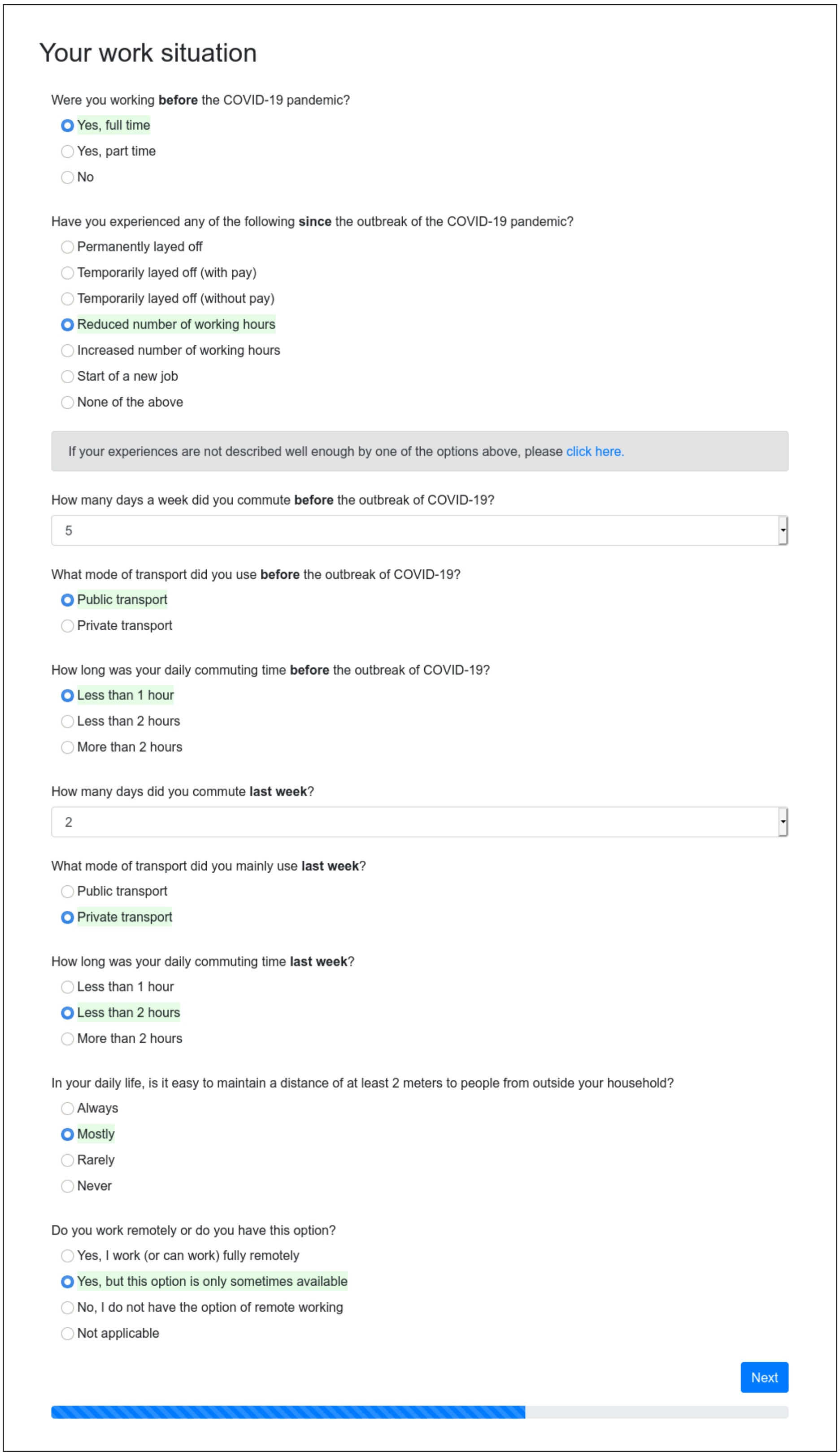

Your work situation

Were you working **before** the COVID-19 pandemic?

- (selected) Yes, full time
- Yes, part time
- No

Have you experienced any of the following **since** the outbreak of the COVID-19 pandemic?

- Permanently layed off
- Temporarily layed off (with pay)
- Temporarily layed off (without pay)
- (selected) Reduced number of working hours
- Increased number of working hours
- Start of a new job
- None of the above

If your experiences are not described well enough by one of the options above, please click here.

How many days a week did you commute **before** the outbreak of COVID-19?

5

What mode of transport did you use **before** the outbreak of COVID-19?

- (selected) Public transport
- Private transport

How long was your daily commuting time **before** the outbreak of COVID-19?

- (selected) Less than 1 hour
- Less than 2 hours
- More than 2 hours

How many days did you commute **last week**?

2

What mode of transport did you mainly use **last week**?

- Public transport
- (selected) Private transport

How long was your daily commuting time **last week**?

- Less than 1 hour
- (selected) Less than 2 hours
- More than 2 hours

In your daily life, is it easy to maintain a distance of at least 2 meters to people from outside your household?

- Always
- (selected) Mostly
- Rarely
- Never

Do you work remotely or do you have this option?

- Yes, I work (or can work) fully remotely
- (selected) Yes, but this option is only sometimes available
- No, I do not have the option of remote working
- Not applicable

Next

Figure G15: Financial situation

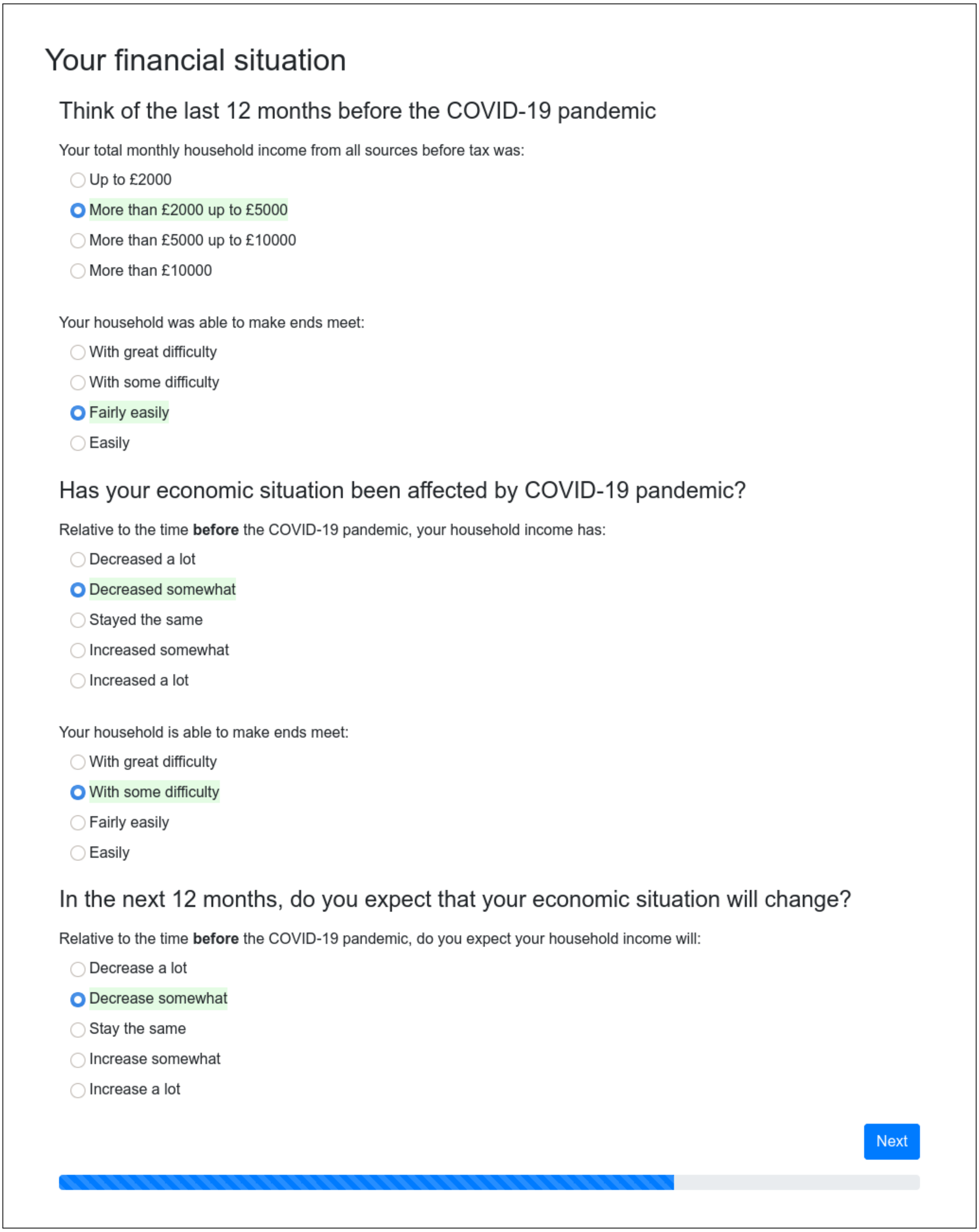

Your financial situation

Think of the last 12 months before the COVID-19 pandemic

Your total monthly household income from all sources before tax was:

- ○ Up to £2000
- ● More than £2000 up to £5000
- ○ More than £5000 up to £10000
- ○ More than £10000

Your household was able to make ends meet:

- ○ With great difficulty
- ○ With some difficulty
- ● Fairly easily
- ○ Easily

Has your economic situation been affected by COVID-19 pandemic?

Relative to the time **before** the COVID-19 pandemic, your household income has:

- ○ Decreased a lot
- ● Decreased somewhat
- ○ Stayed the same
- ○ Increased somewhat
- ○ Increased a lot

Your household is able to make ends meet:

- ○ With great difficulty
- ● With some difficulty
- ○ Fairly easily
- ○ Easily

In the next 12 months, do you expect that your economic situation will change?

Relative to the time **before** the COVID-19 pandemic, do you expect your household income will:

- ○ Decrease a lot
- ● Decrease somewhat
- ○ Stay the same
- ○ Increase somewhat
- ○ Increase a lot

Next

Figure G16: Economic situation

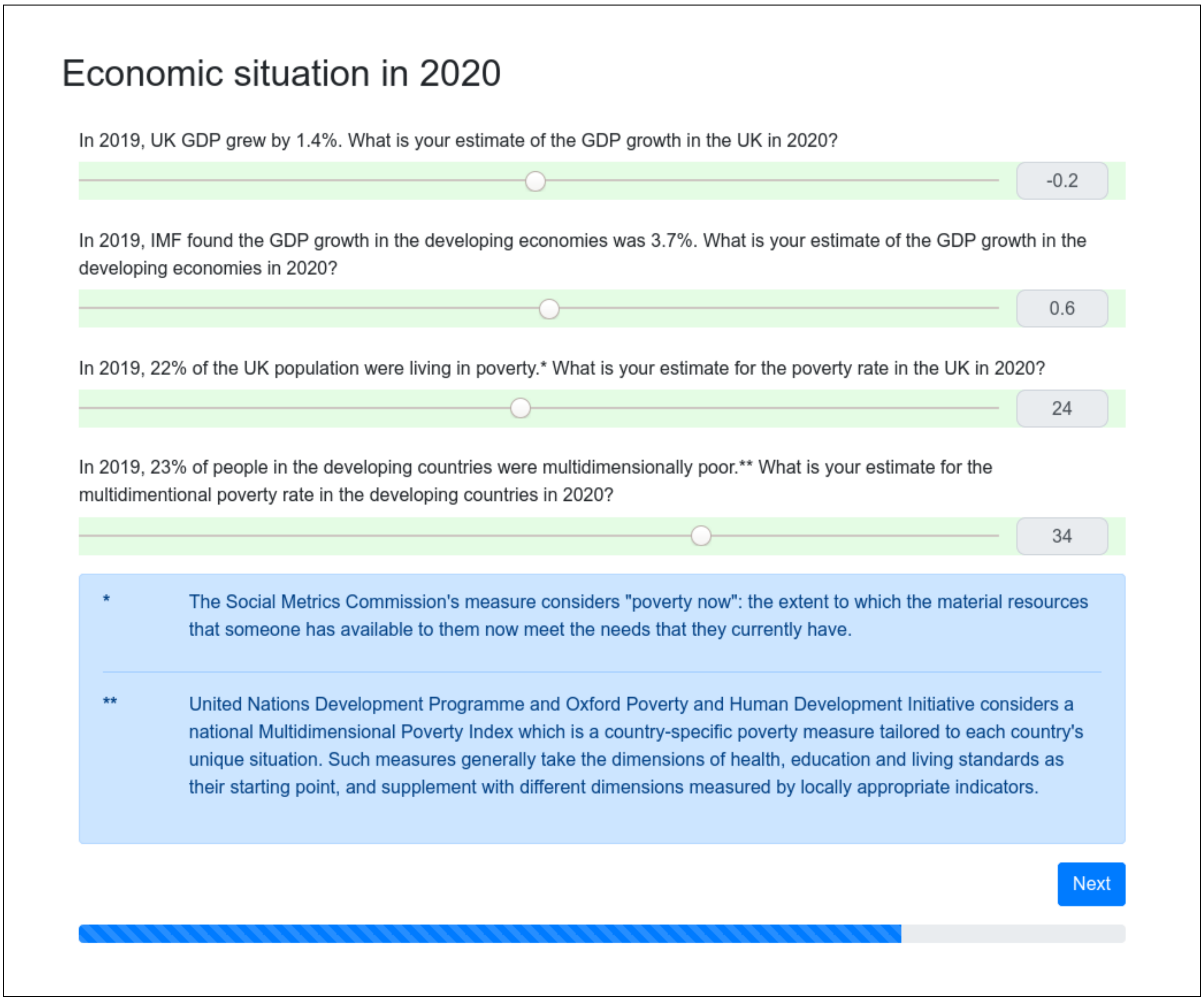

Economic situation in 2020

In 2019, UK GDP grew by 1.4%. What is your estimate of the GDP growth in the UK in 2020?

-0.2

In 2019, IMF found the GDP growth in the developing economies was 3.7%. What is your estimate of the GDP growth in the developing economies in 2020?

0.6

In 2019, 22% of the UK population were living in poverty.* What is your estimate for the poverty rate in the UK in 2020?

24

In 2019, 23% of people in the developing countries were multidimensionally poor.** What is your estimate for the multidimentional poverty rate in the developing countries in 2020?

34

* The Social Metrics Commission's measure considers "poverty now": the extent to which the material resources that someone has available to them now meet the needs that they currently have.

** United Nations Development Programme and Oxford Poverty and Human Development Initiative considers a national Multidimensional Poverty Index which is a country-specific poverty measure tailored to each country's unique situation. Such measures generally take the dimensions of health, education and living standards as their starting point, and supplement with different dimensions measured by locally appropriate indicators.

Next

Figure G17: Main news sources

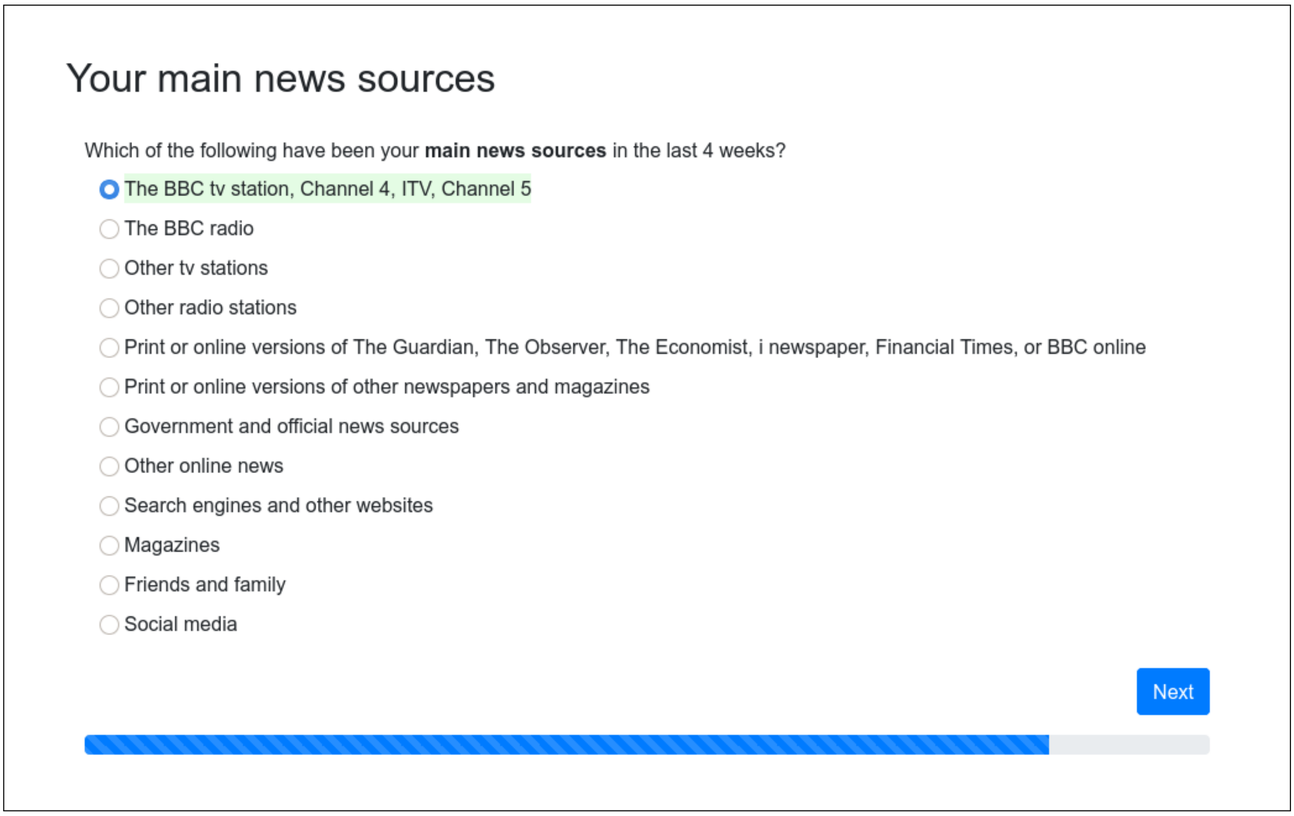

Figure G18: COVID-19 risks and policies

COVID-19 risks and policies to counteract the pandemic

What do you think about the risks from COVID-19?

- ◯ Most people have no symptoms or only few flu-like symptoms; otherwise COVID-19 isn't very dangerous.
- ◯ Only people with several pre-existing conditions die after getting COVID-19 and any of those conditions could have been the reason for their death.
- ◯ Older members of the population and risk groups can become seriously ill after infection with COVID-19.
- ◉ Anyone can become seriously ill after infection with COVID-19.
- ◯ Many people infected with COVID-19 become seriously ill.
- ◯ Most people infected with COVID-19 become seriously ill.

What do you think about the lockdown to contain the spread of COVID-19?

- ◯ There shouldn't have been any lockdown; it is not clear whether it helps at all.
- ◯ The lockdown is damaging to the economy and the health benefits of the lockdown do not outweigh the economic risks.
- ◯ The lockdown is harming the economy the health benefits of the lockdown might not outweigh the economic risks.
- ◉ The lockdown is necessary to contain the spread of COVID-19.
- ◯ No measure is too costly to contain the spread of COVID-19.

The policies against the spread of COVID-19 taken by the UK government are overall:

- ◯ Much too lax
- ◉ Somewhat too lax
- ◯ Just right
- ◯ Somewhat too harsh
- ◯ Much too harsh

With which statement do you agree more?

- ◯ The Coronavirus is man-made.
- ◉ The Coronavirus is natural in origin.

With which statement do you agree more?

- ◯ The Coronavirus was spread on purpose.
- ◉ The Coronavirus was spread unintentionally.

When do you think the COVID-19 pandemic will be overcome in the UK?

In [16] months

When do you think that a vaccination against COVID-19 will be widely available in UK?

In [12] months

When available, will you be willing to get vaccinated against COVID-19?

- ◯ Definitely not
- ◯ Probably not
- ◯ Not sure
- ◉ Rather yes
- ◯ Definitely yes

Next

Figure G19: Empathy

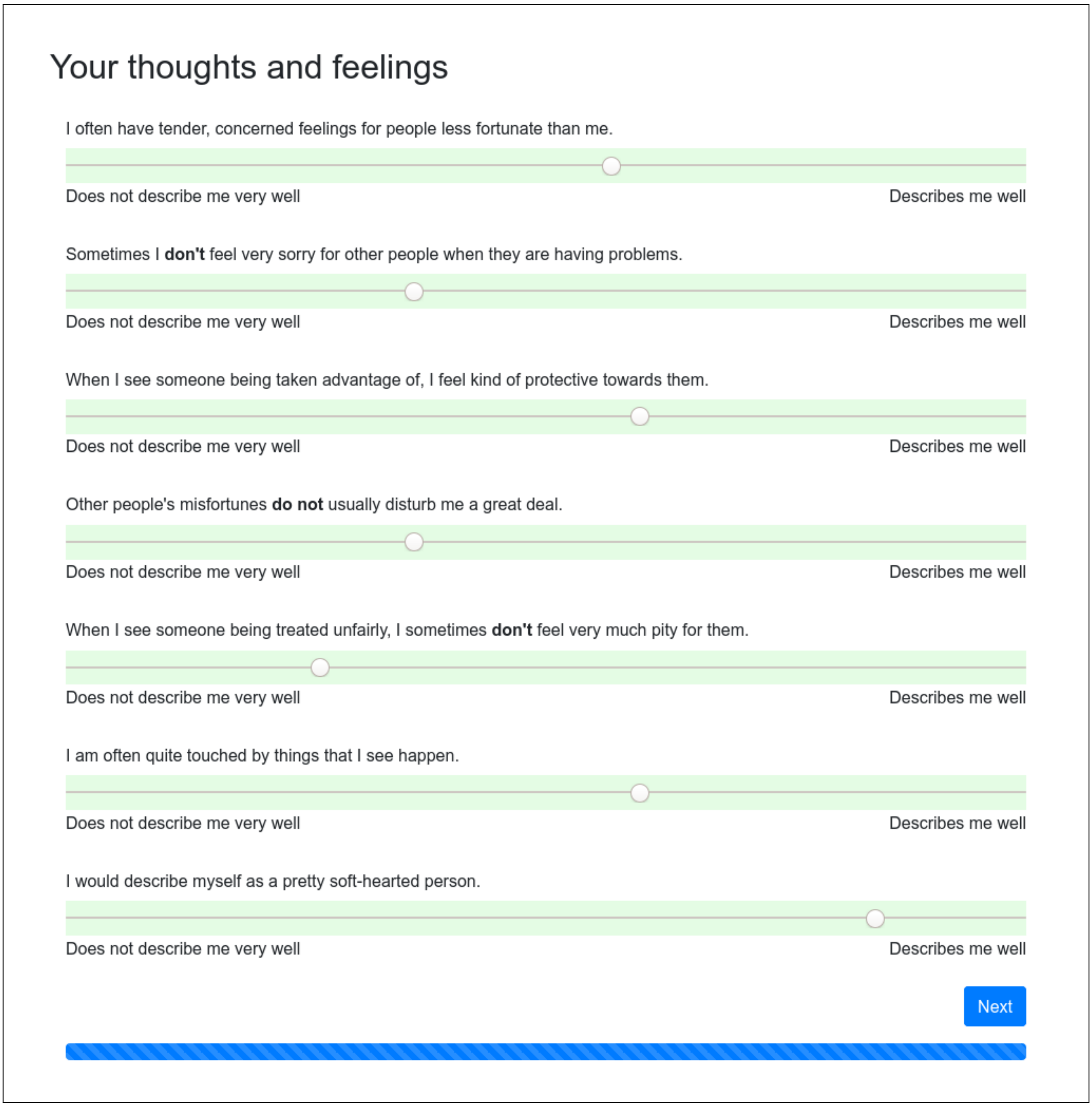

Your thoughts and feelings

I often have tender, concerned feelings for people less fortunate than me.

Does not describe me very well — Describes me well

Sometimes I **don't** feel very sorry for other people when they are having problems.

Does not describe me very well — Describes me well

When I see someone being taken advantage of, I feel kind of protective towards them.

Does not describe me very well — Describes me well

Other people's misfortunes **do not** usually disturb me a great deal.

Does not describe me very well — Describes me well

When I see someone being treated unfairly, I sometimes **don't** feel very much pity for them.

Does not describe me very well — Describes me well

I am often quite touched by things that I see happen.

Does not describe me very well — Describes me well

I would describe myself as a pretty soft-hearted person.

Does not describe me very well — Describes me well

Next

Figure G20: Payment info - Donors

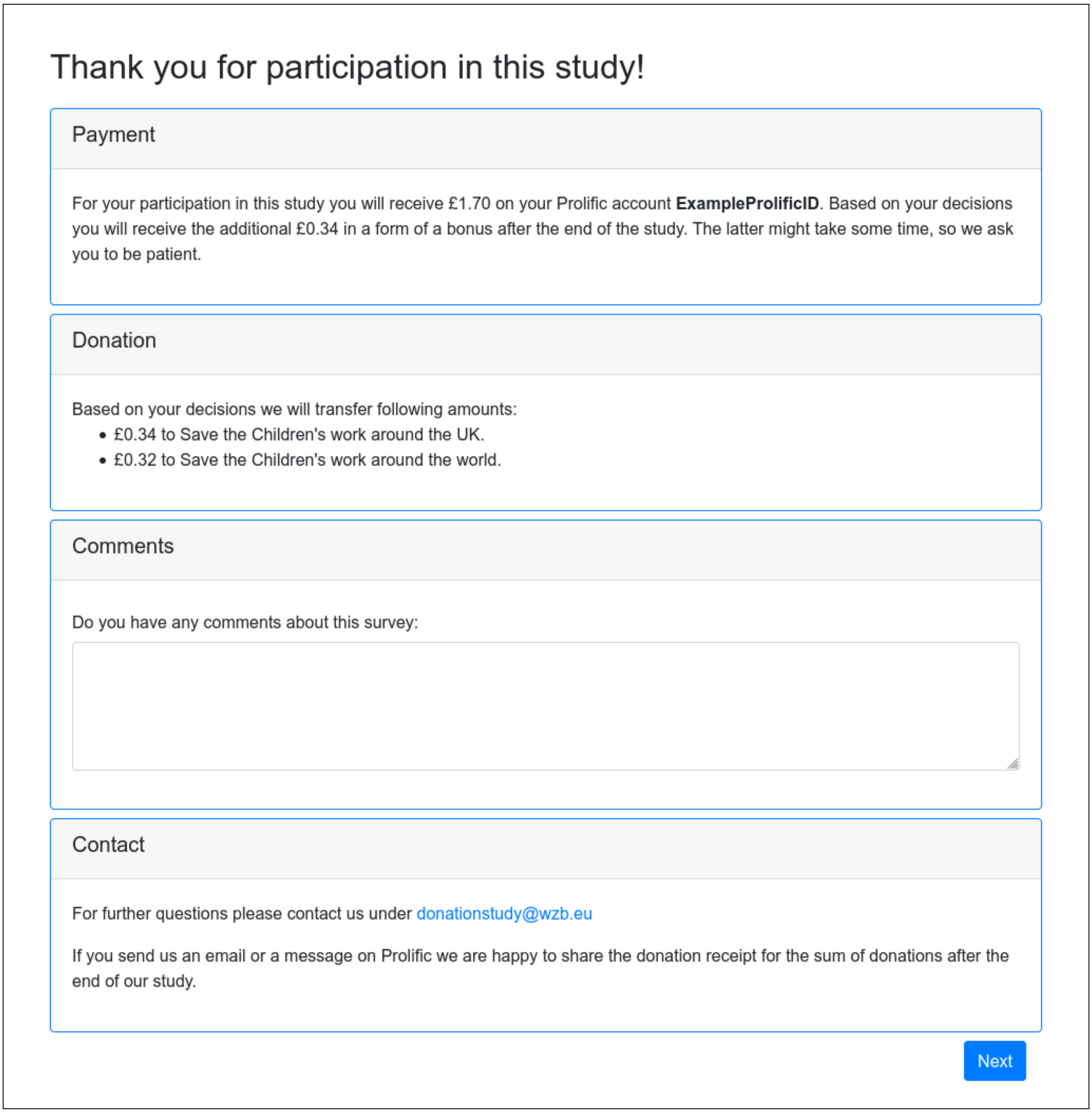

Thank you for participation in this study!

Payment

For your participation in this study you will receive £1.70 on your Prolific account **ExampleProlificID**. Based on your decisions you will receive the additional £0.34 in a form of a bonus after the end of the study. The latter might take some time, so we ask you to be patient.

Donation

Based on your decisions we will transfer following amounts:

- £0.34 to Save the Children's work around the UK.
- £0.32 to Save the Children's work around the world.

Comments

Do you have any comments about this survey:

Contact

For further questions please contact us under donationstudy@wzb.eu

If you send us an email or a message on Prolific we are happy to share the donation receipt for the sum of donations after the end of our study.

Next

Figure G21: Payment info - Non-donors

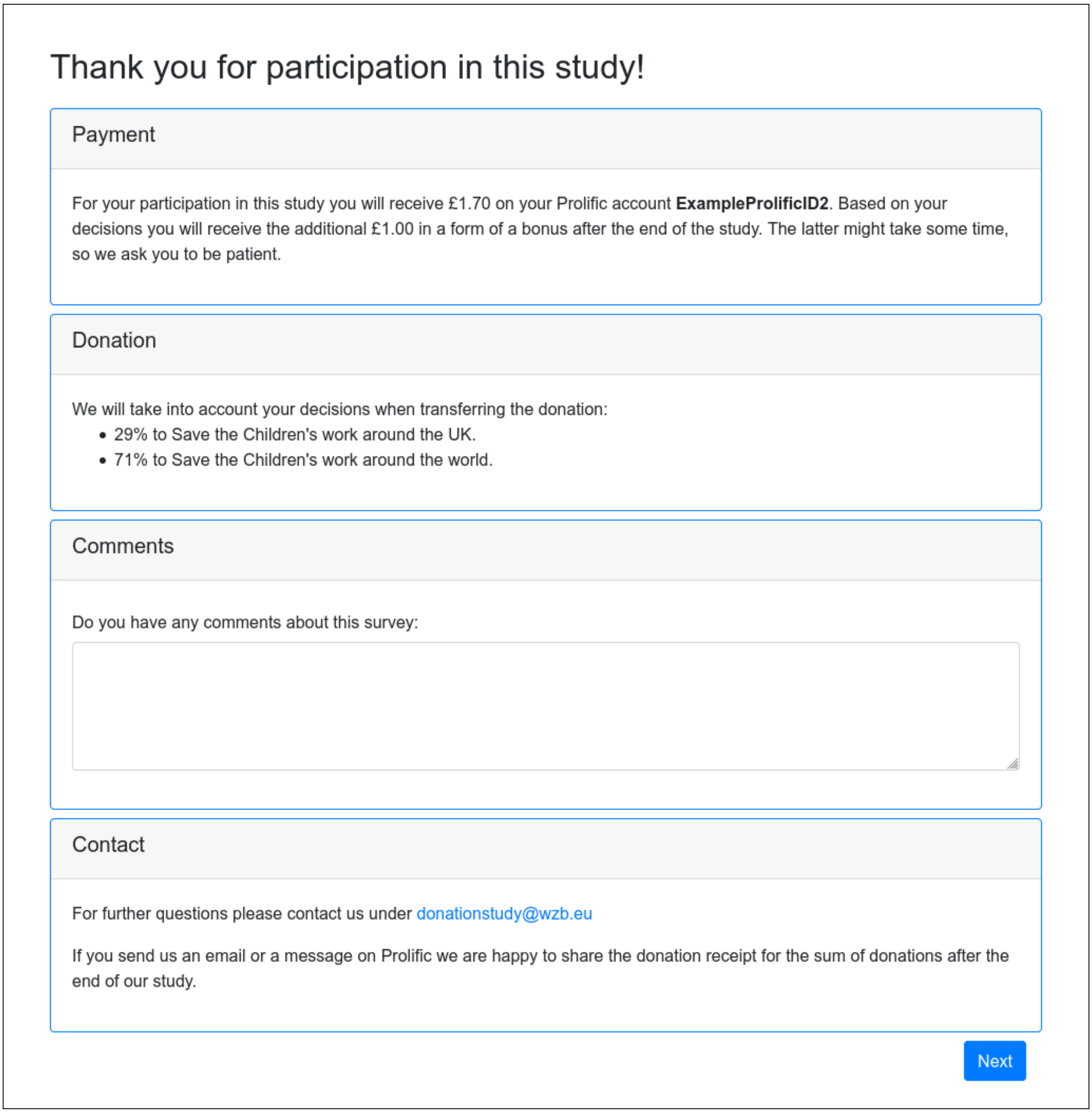

Thank you for participation in this study!

Payment

For your participation in this study you will receive £1.70 on your Prolific account **ExampleProlificID2**. Based on your decisions you will receive the additional £1.00 in a form of a bonus after the end of the study. The latter might take some time, so we ask you to be patient.

Donation

We will take into account your decisions when transferring the donation:

- 29% to Save the Children's work around the UK.
- 71% to Save the Children's work around the world.

Comments

Do you have any comments about this survey:

Contact

For further questions please contact us under donationstudy@wzb.eu

If you send us an email or a message on Prolific we are happy to share the donation receipt for the sum of donations after the end of our study.

Next

H Screenshots from the additional survey experiment

Figure H22: Introduction

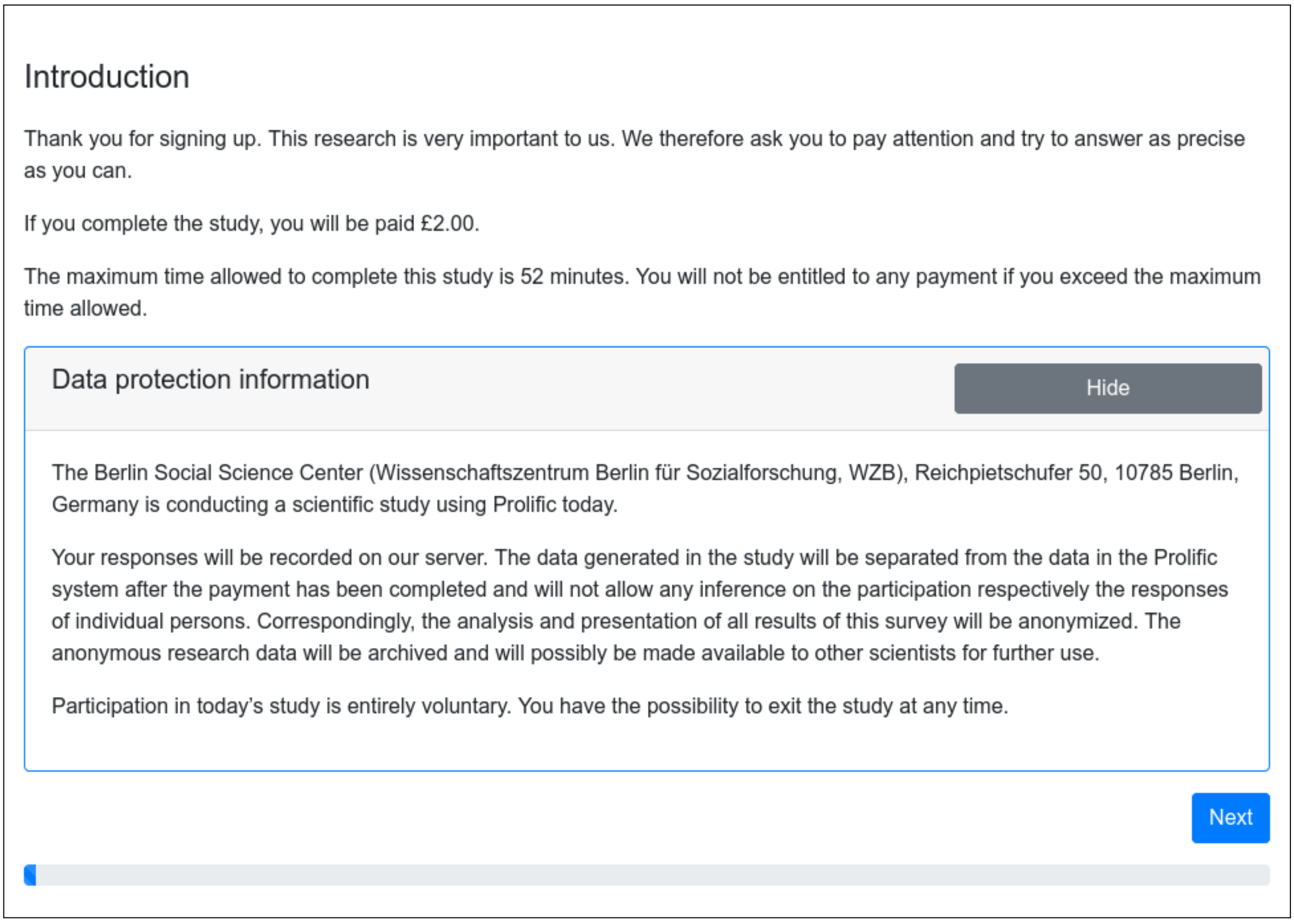

Introduction

Thank you for signing up. This research is very important to us. We therefore ask you to pay attention and try to answer as precise as you can.

If you complete the study, you will be paid £2.00.

The maximum time allowed to complete this study is 52 minutes. You will not be entitled to any payment if you exceed the maximum time allowed.

Data protection information

Hide

The Berlin Social Science Center (Wissenschaftszentrum Berlin für Sozialforschung, WZB), Reichpietschufer 50, 10785 Berlin, Germany is conducting a scientific study using Prolific today.

Your responses will be recorded on our server. The data generated in the study will be separated from the data in the Prolific system after the payment has been completed and will not allow any inference on the participation respectively the responses of individual persons. Correspondingly, the analysis and presentation of all results of this survey will be anonymized. The anonymous research data will be archived and will possibly be made available to other scientists for further use.

Participation in today's study is entirely voluntary. You have the possibility to exit the study at any time.

Next

Figure H23: Control condition without COVID-19 frame

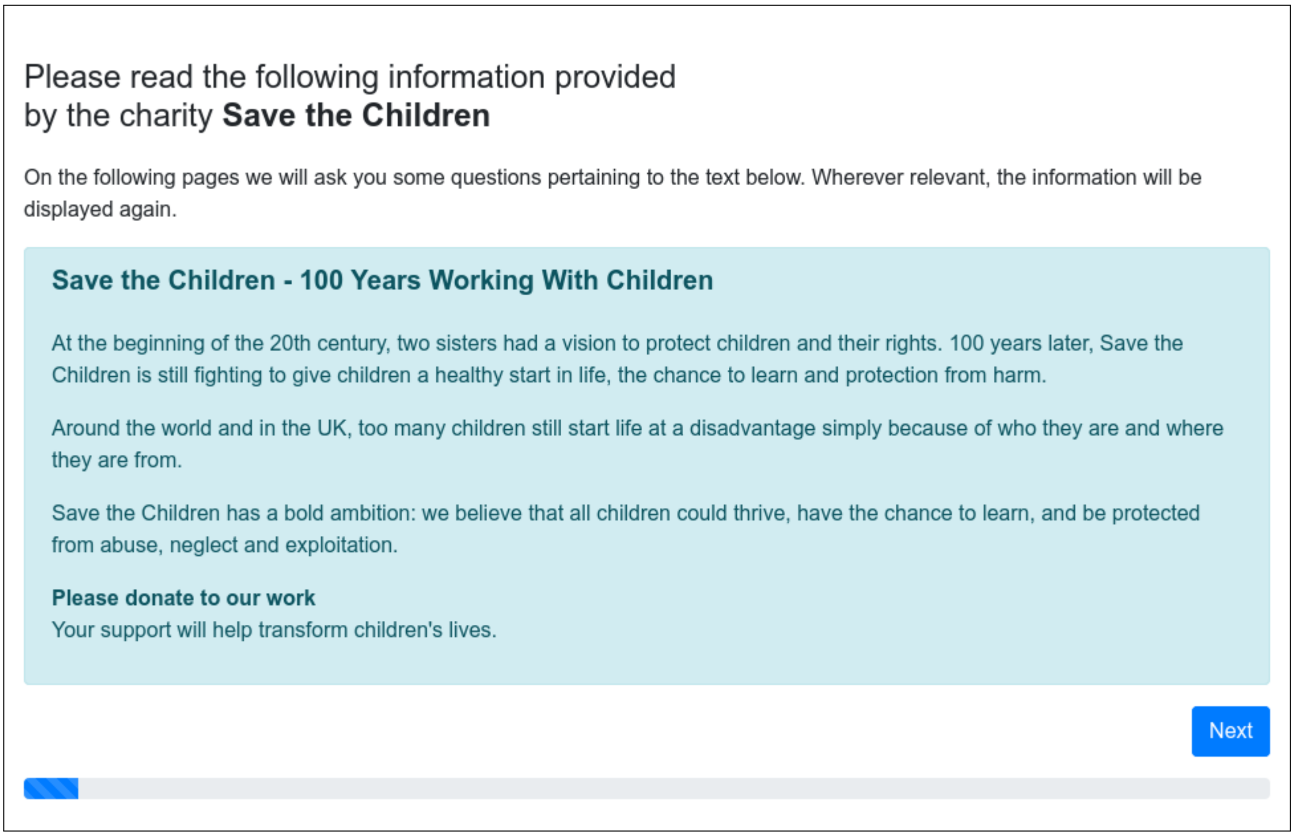

Figure H24: Treatment condition with COVID-19 frame

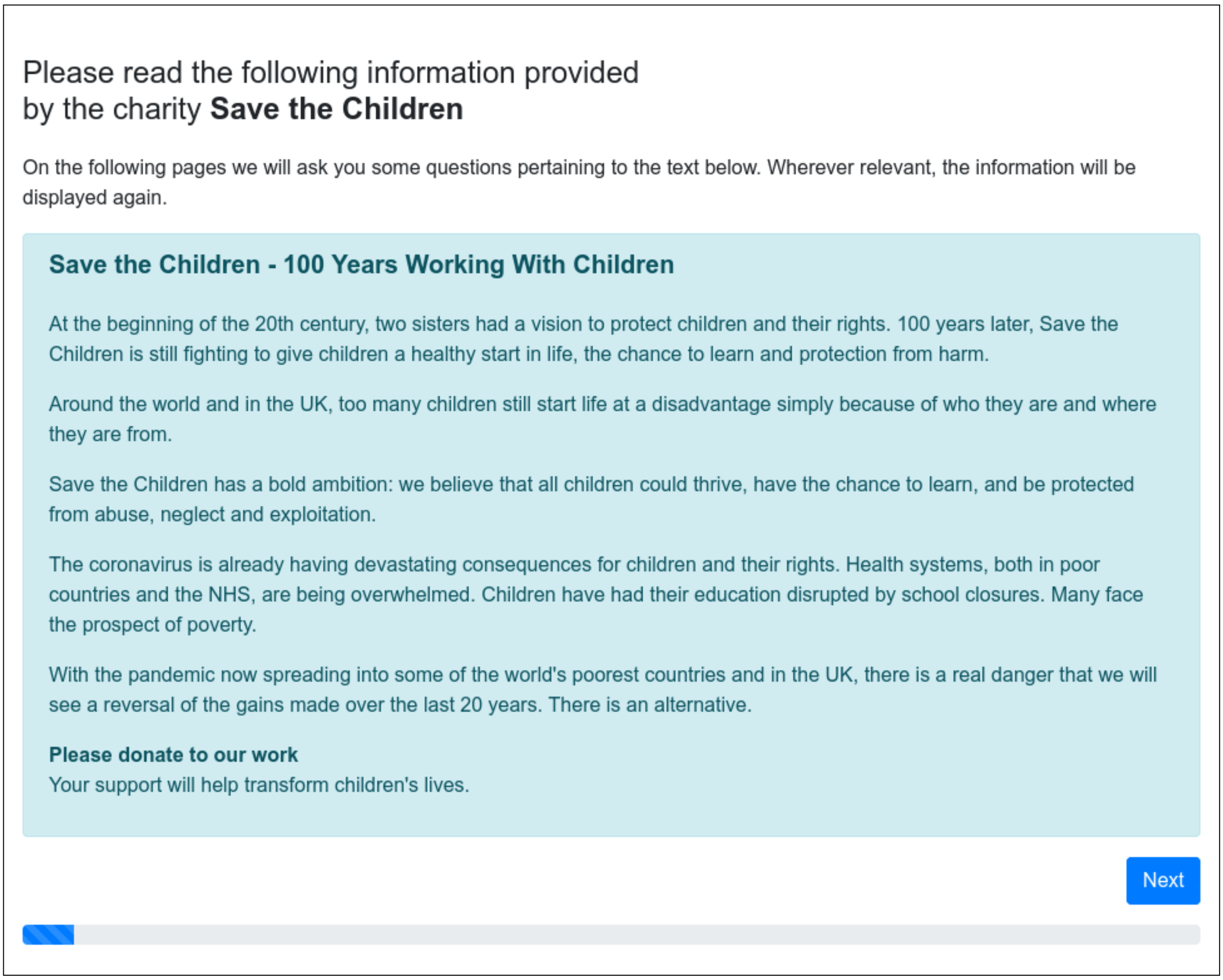
Please read the following information provided
by the charity **Save the Children**

On the following pages we will ask you some questions pertaining to the text below. Wherever relevant, the information will be displayed again.

Save the Children - 100 Years Working With Children

At the beginning of the 20th century, two sisters had a vision to protect children and their rights. 100 years later, Save the Children is still fighting to give children a healthy start in life, the chance to learn and protection from harm.

Around the world and in the UK, too many children still start life at a disadvantage simply because of who they are and where they are from.

Save the Children has a bold ambition: we believe that all children could thrive, have the chance to learn, and be protected from abuse, neglect and exploitation.

The coronavirus is already having devastating consequences for children and their rights. Health systems, both in poor countries and the NHS, are being overwhelmed. Children have had their education disrupted by school closures. Many face the prospect of poverty.

With the pandemic now spreading into some of the world's poorest countries and in the UK, there is a real danger that we will see a reversal of the gains made over the last 20 years. There is an alternative.

Please donate to our work
Your support will help transform children's lives.

Next

Figure H25: Emotion elicitation

Please think of an average Prolific participant from the UK who might receive this donation appeal.

Save the Children - 100 Years Working With Children

Toggle text

At the beginning of the 20th century, two sisters had a vision to protect children and their rights. 100 years later, Save the Children is still fighting to give children a healthy start in life, the chance to learn and protection from harm.

Around the world and in the UK, too many children still start life at a disadvantage simply because of who they are and where they are from.

Save the Children has a bold ambition: we believe that all children could thrive, have the chance to learn, and be protected from abuse, neglect and exploitation.

Please donate to our work
Your support will help transform children's lives.

How strongly would this appeal evoke the following emotions in such a person?

Compassion:

Not at all — A lot

Contentment:

Not at all — A lot

Sadness:

Not at all — A lot

Contempt:

Not at all — A lot

Guilt:

Not at all — A lot

Joy:

Not at all — A lot

Figure H26: Expected use of donations

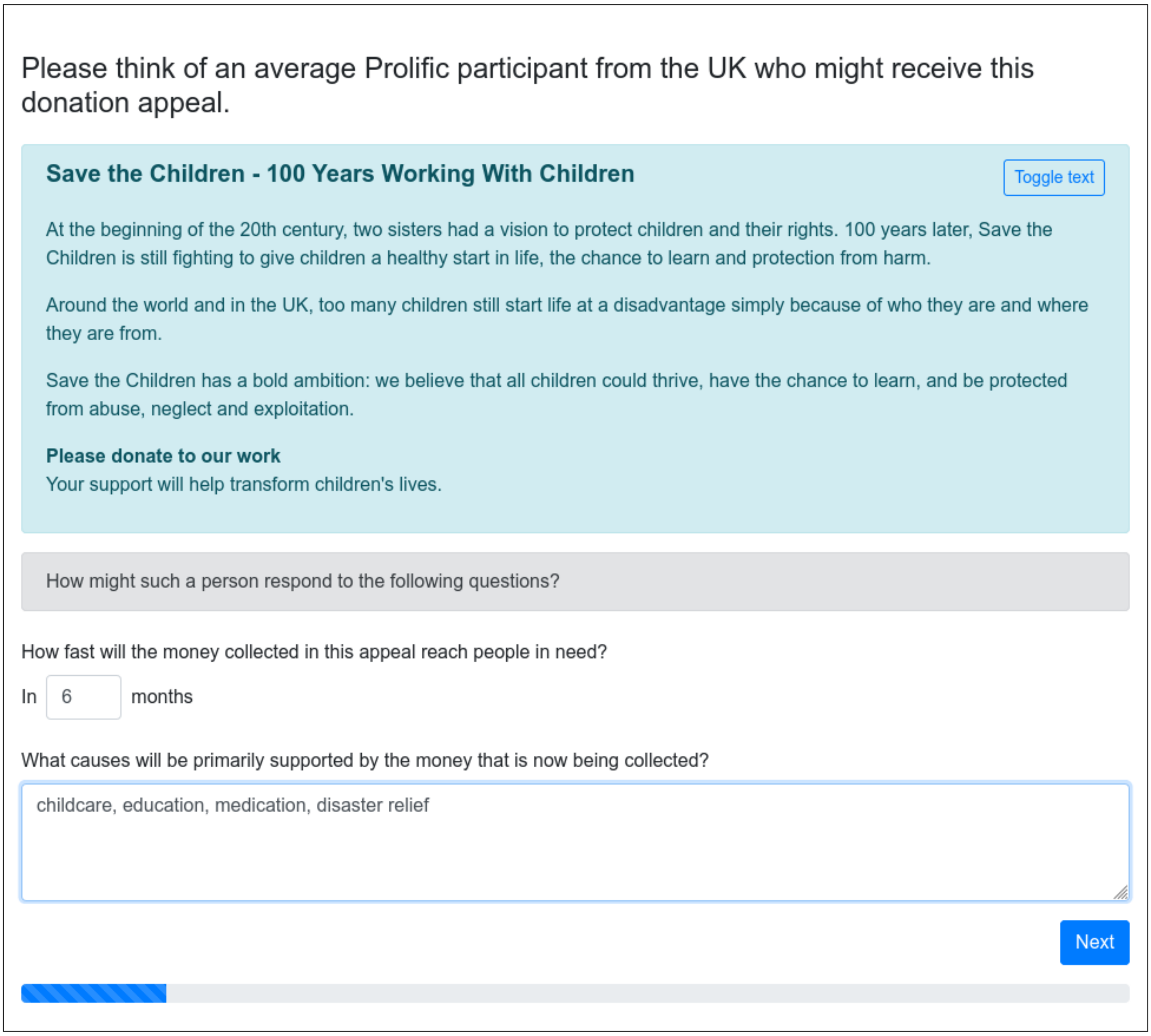

Please think of an average Prolific participant from the UK who might receive this donation appeal.

Save the Children - 100 Years Working With Children

Toggle text

At the beginning of the 20th century, two sisters had a vision to protect children and their rights. 100 years later, Save the Children is still fighting to give children a healthy start in life, the chance to learn and protection from harm.

Around the world and in the UK, too many children still start life at a disadvantage simply because of who they are and where they are from.

Save the Children has a bold ambition: we believe that all children could thrive, have the chance to learn, and be protected from abuse, neglect and exploitation.

Please donate to our work
Your support will help transform children's lives.

How might such a person respond to the following questions?

How fast will the money collected in this appeal reach people in need?

In 6 months

What causes will be primarily supported by the money that is now being collected?

childcare, education, medication, disaster relief

Next

Figure H27: Perceived pressure when receiving donation appeal via Royal Mail

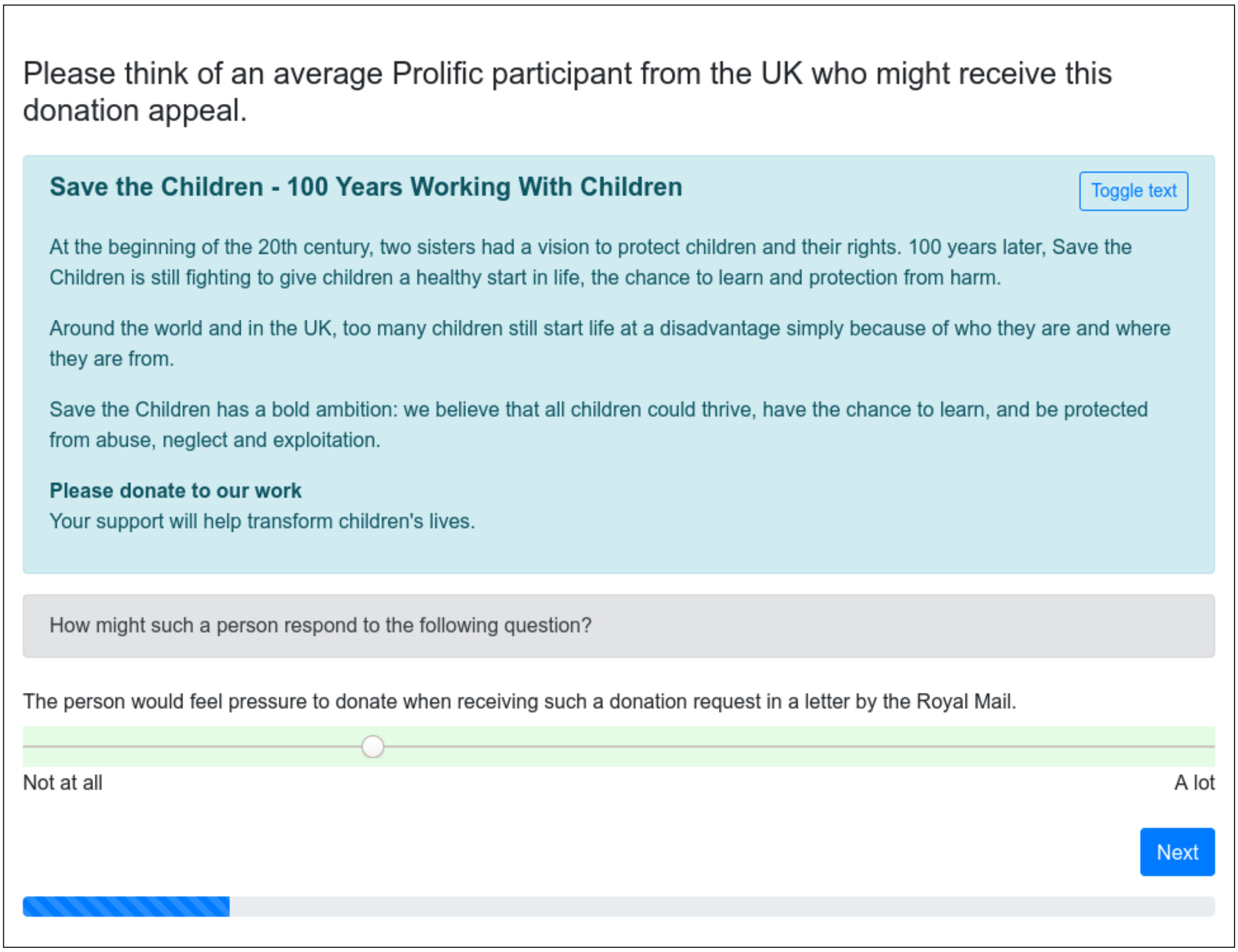

Figure H28: Perceived pressure when receiving donation appeal on Prolific

Please think of an average Prolific participant from the UK who might receive this donation appeal.

Save the Children - 100 Years Working With Children

Toggle text

At the beginning of the 20th century, two sisters had a vision to protect children and their rights. 100 years later, Save the Children is still fighting to give children a healthy start in life, the chance to learn and protection from harm.

Around the world and in the UK, too many children still start life at a disadvantage simply because of who they are and where they are from.

Save the Children has a bold ambition: we believe that all children could thrive, have the chance to learn, and be protected from abuse, neglect and exploitation.

The coronavirus is already having devastating consequences for children and their rights. Health systems, both in poor countries and the NHS, are being overwhelmed. Children have had their education disrupted by school closures. Many face the prospect of poverty.

With the pandemic now spreading into some of the world's poorest countries and in the UK, there is a real danger that we will see a reversal of the gains made over the last 20 years. There is an alternative.

Please donate to our work
Your support will help transform children's lives.

How might such a person respond to the following question?

The person would feel pressure to donate when receiving such a donation request in a study on Prolific.

Not at all — A lot

Next

Figure H29: Information about Transparency International

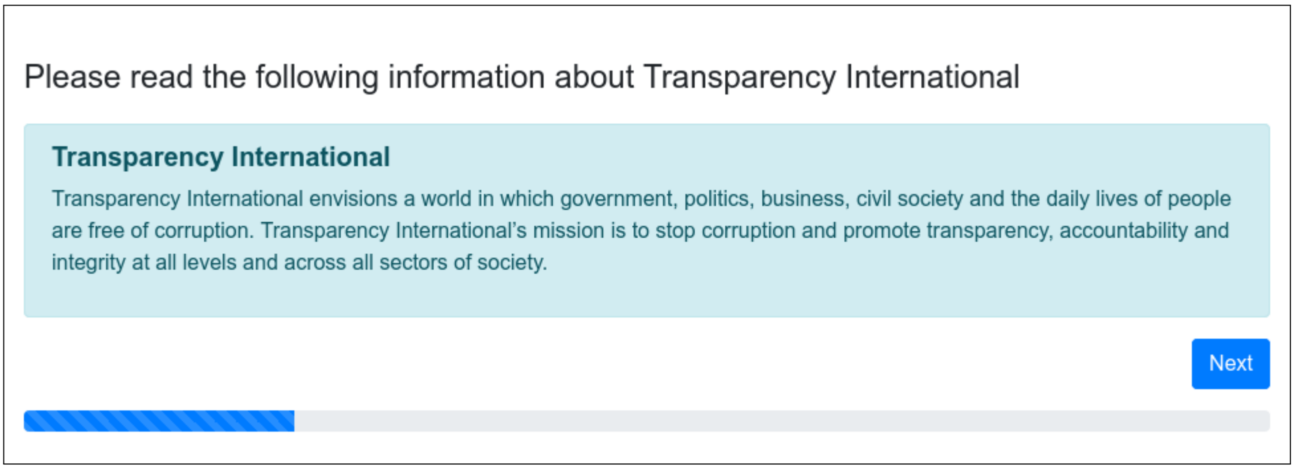

Figure H30: Comparison between charities: Transparency International

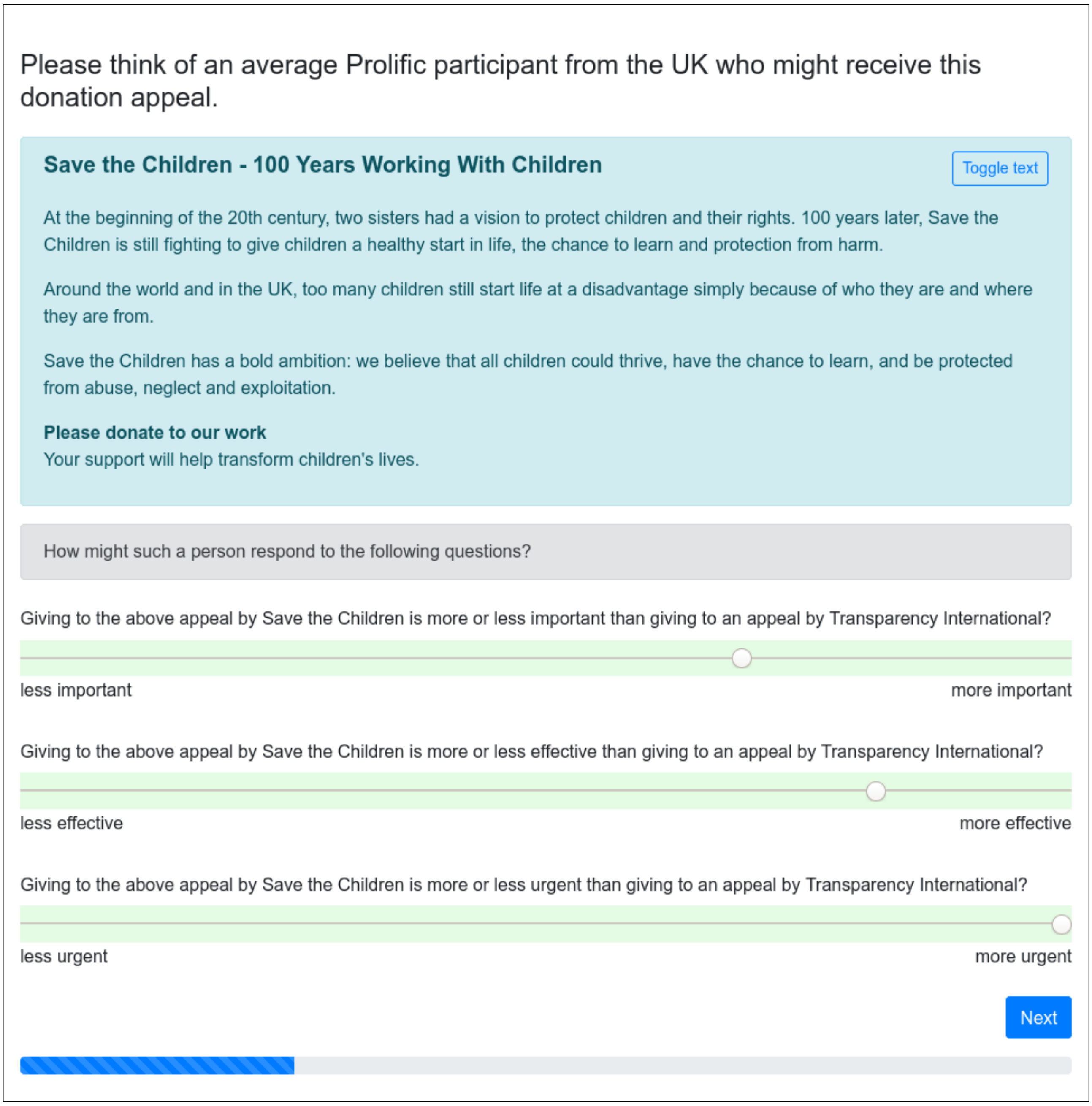
Please think of an average Prolific participant from the UK who might receive this donation appeal.

Save the Children - 100 Years Working With Children

Toggle text

At the beginning of the 20th century, two sisters had a vision to protect children and their rights. 100 years later, Save the Children is still fighting to give children a healthy start in life, the chance to learn and protection from harm.

Around the world and in the UK, too many children still start life at a disadvantage simply because of who they are and where they are from.

Save the Children has a bold ambition: we believe that all children could thrive, have the chance to learn, and be protected from abuse, neglect and exploitation.

Please donate to our work
Your support will help transform children's lives.

How might such a person respond to the following questions?

Giving to the above appeal by Save the Children is more or less important than giving to an appeal by Transparency International?

less important — more important

Giving to the above appeal by Save the Children is more or less effective than giving to an appeal by Transparency International?

less effective — more effective

Giving to the above appeal by Save the Children is more or less urgent than giving to an appeal by Transparency International?

less urgent — more urgent

Next

Figure H31: Information about Alzheimer's Society

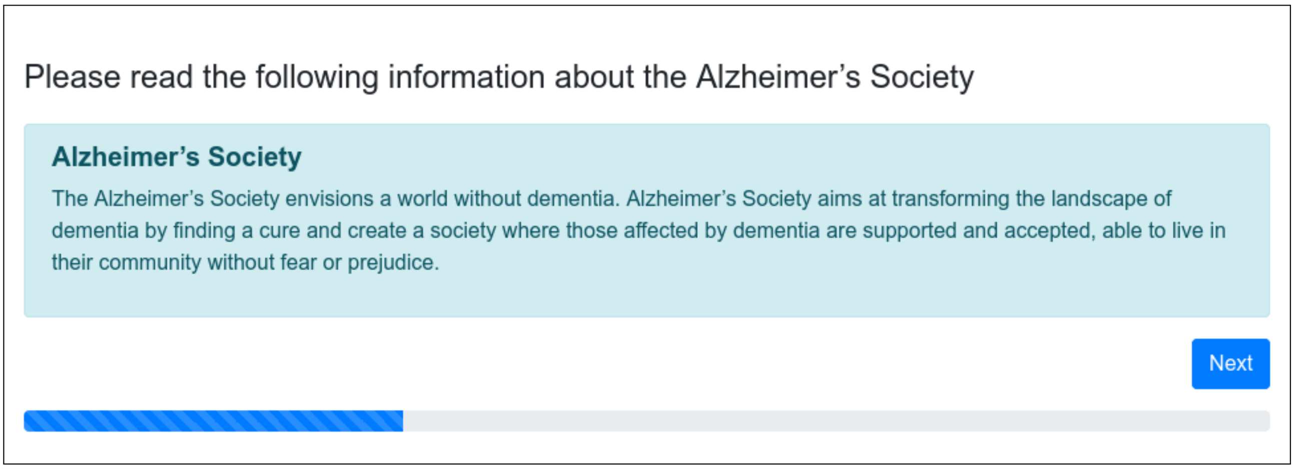

Figure H32: Comparison between charities: Alzheimer's Society

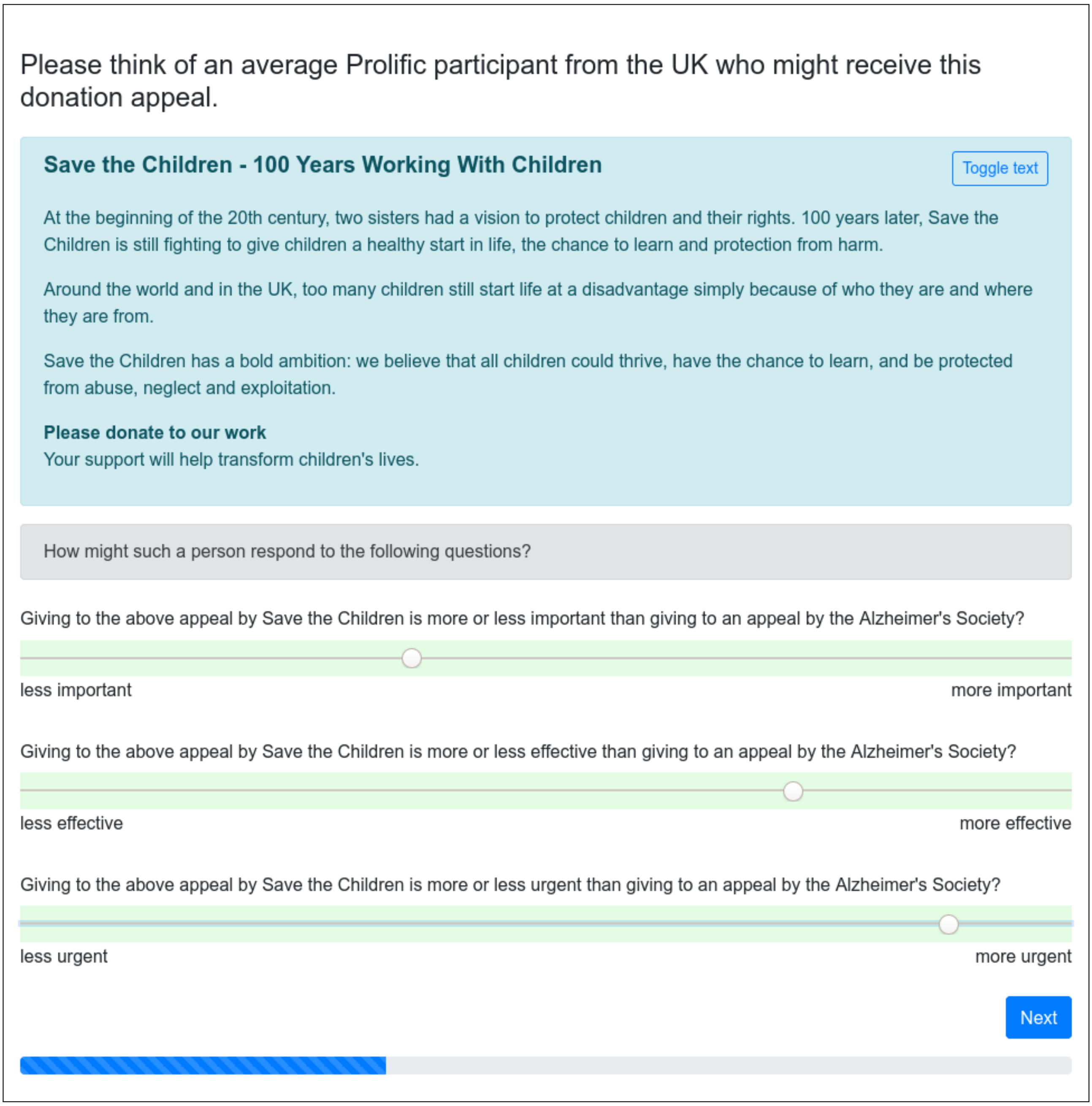

Please think of an average Prolific participant from the UK who might receive this donation appeal.

Save the Children - 100 Years Working With Children

Toggle text

At the beginning of the 20th century, two sisters had a vision to protect children and their rights. 100 years later, Save the Children is still fighting to give children a healthy start in life, the chance to learn and protection from harm.

Around the world and in the UK, too many children still start life at a disadvantage simply because of who they are and where they are from.

Save the Children has a bold ambition: we believe that all children could thrive, have the chance to learn, and be protected from abuse, neglect and exploitation.

Please donate to our work
Your support will help transform children's lives.

How might such a person respond to the following questions?

Giving to the above appeal by Save the Children is more or less important than giving to an appeal by the Alzheimer's Society?

less important | more important

Giving to the above appeal by Save the Children is more or less effective than giving to an appeal by the Alzheimer's Society?

less effective | more effective

Giving to the above appeal by Save the Children is more or less urgent than giving to an appeal by the Alzheimer's Society?

less urgent | more urgent

Next

Figure H33: Information about World Wildlife Fund

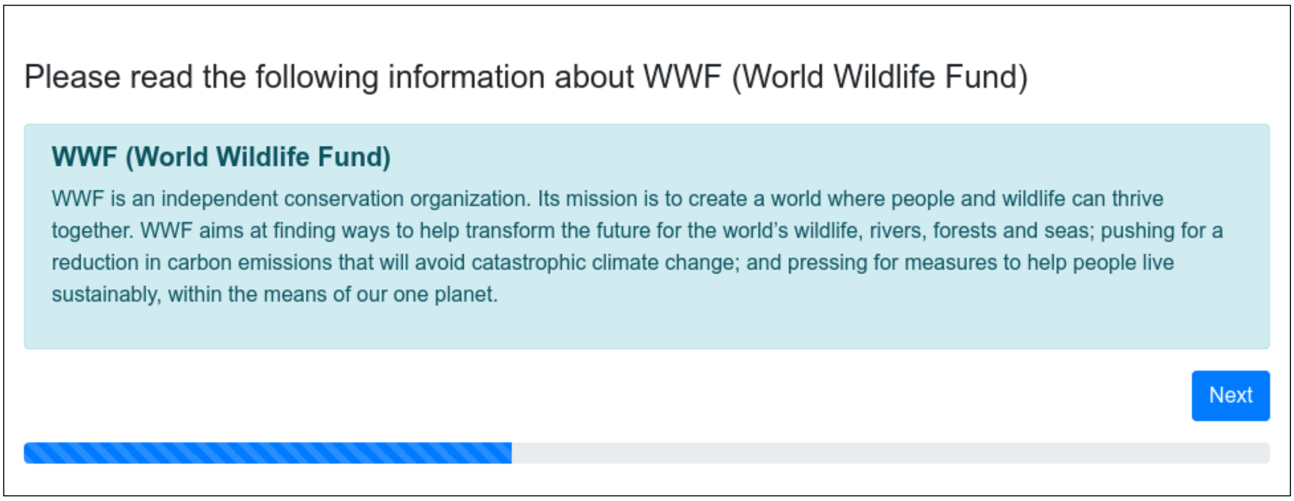

Figure H34: Comparison between charities: World Wildlife Fund

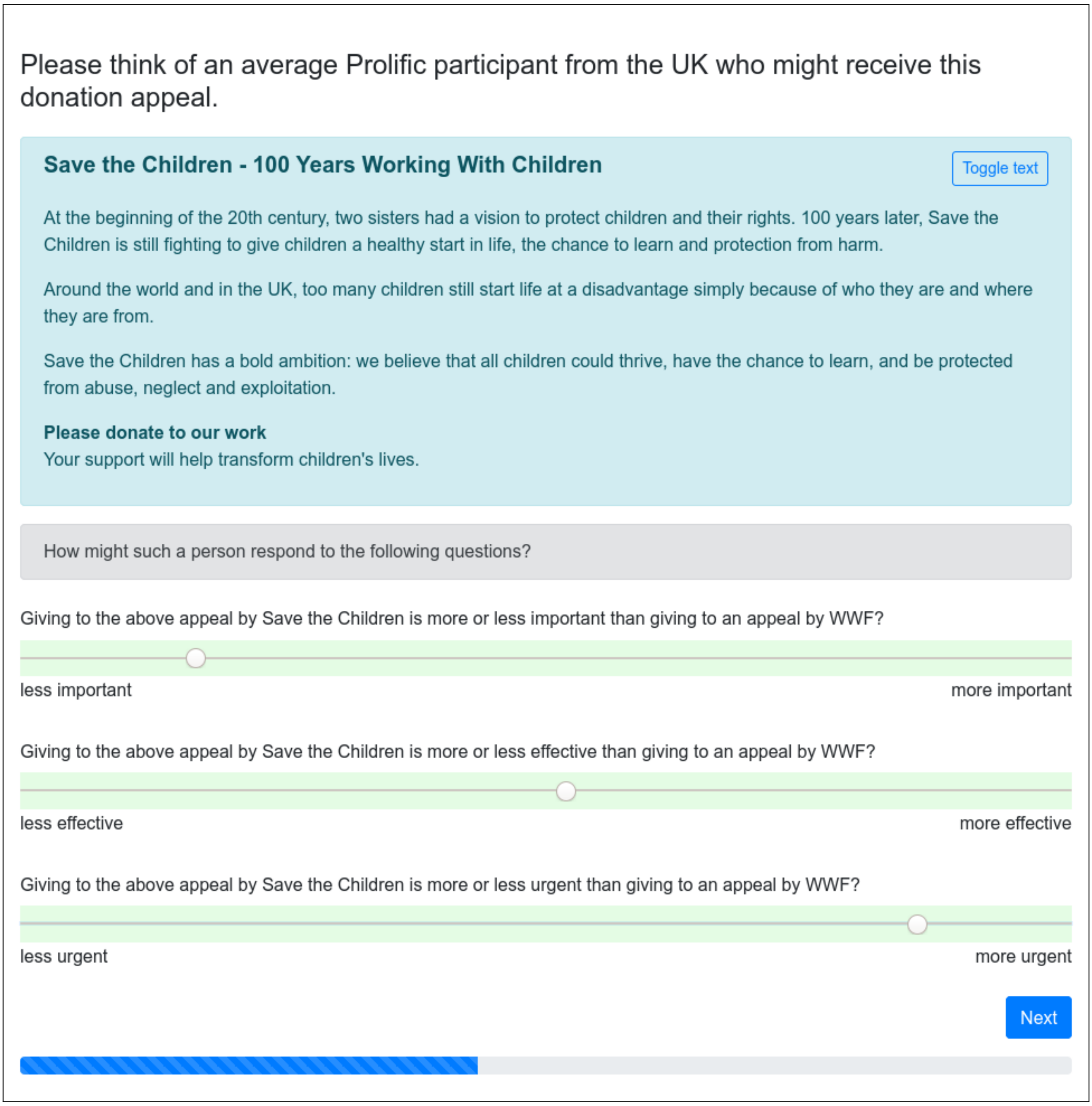

Please think of an average Prolific participant from the UK who might receive this donation appeal.

Save the Children - 100 Years Working With Children

Toggle text

At the beginning of the 20th century, two sisters had a vision to protect children and their rights. 100 years later, Save the Children is still fighting to give children a healthy start in life, the chance to learn and protection from harm.

Around the world and in the UK, too many children still start life at a disadvantage simply because of who they are and where they are from.

Save the Children has a bold ambition: we believe that all children could thrive, have the chance to learn, and be protected from abuse, neglect and exploitation.

Please donate to our work
Your support will help transform children's lives.

How might such a person respond to the following questions?

Giving to the above appeal by Save the Children is more or less important than giving to an appeal by WWF?

less important — more important

Giving to the above appeal by Save the Children is more or less effective than giving to an appeal by WWF?

less effective — more effective

Giving to the above appeal by Save the Children is more or less urgent than giving to an appeal by WWF?

less urgent — more urgent

Next

Figure H35: Introduction to the personal survey

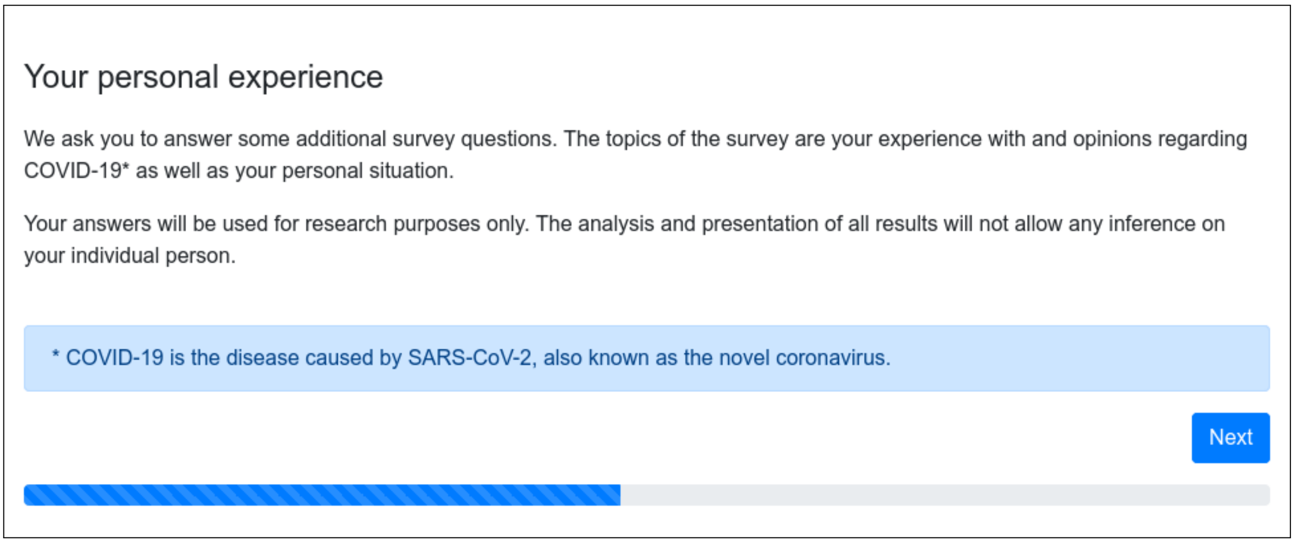

Figure H36: Demographics

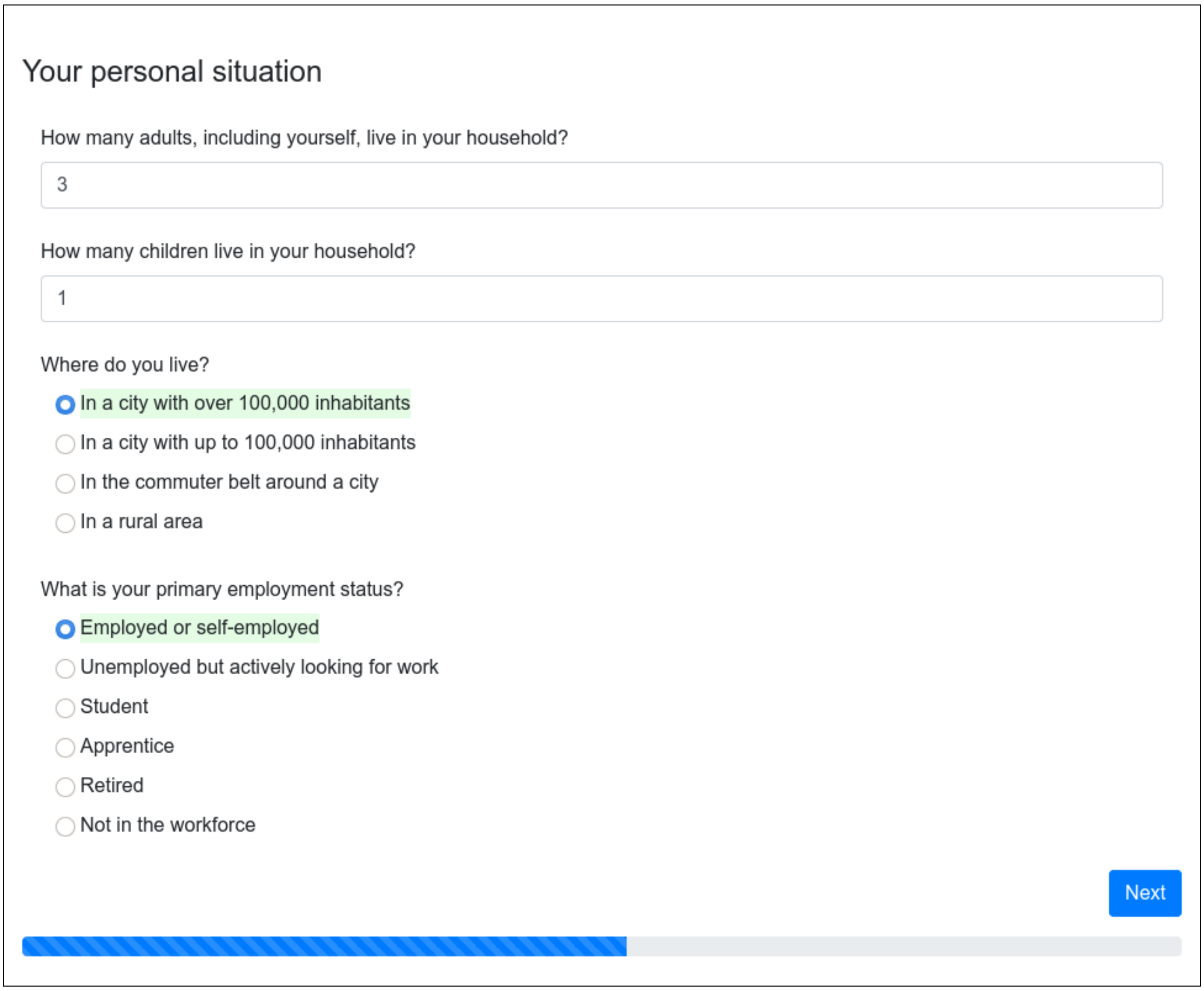
Your personal situation

How many adults, including yourself, live in your household?

3

How many children live in your household?

1

Where do you live?

- (●) In a city with over 100,000 inhabitants
- ( ) In a city with up to 100,000 inhabitants
- ( ) In the commuter belt around a city
- ( ) In a rural area

What is your primary employment status?

- (●) Employed or self-employed
- ( ) Unemployed but actively looking for work
- ( ) Student
- ( ) Apprentice
- ( ) Retired
- ( ) Not in the workforce

Next

Figure H37: Location confirmation

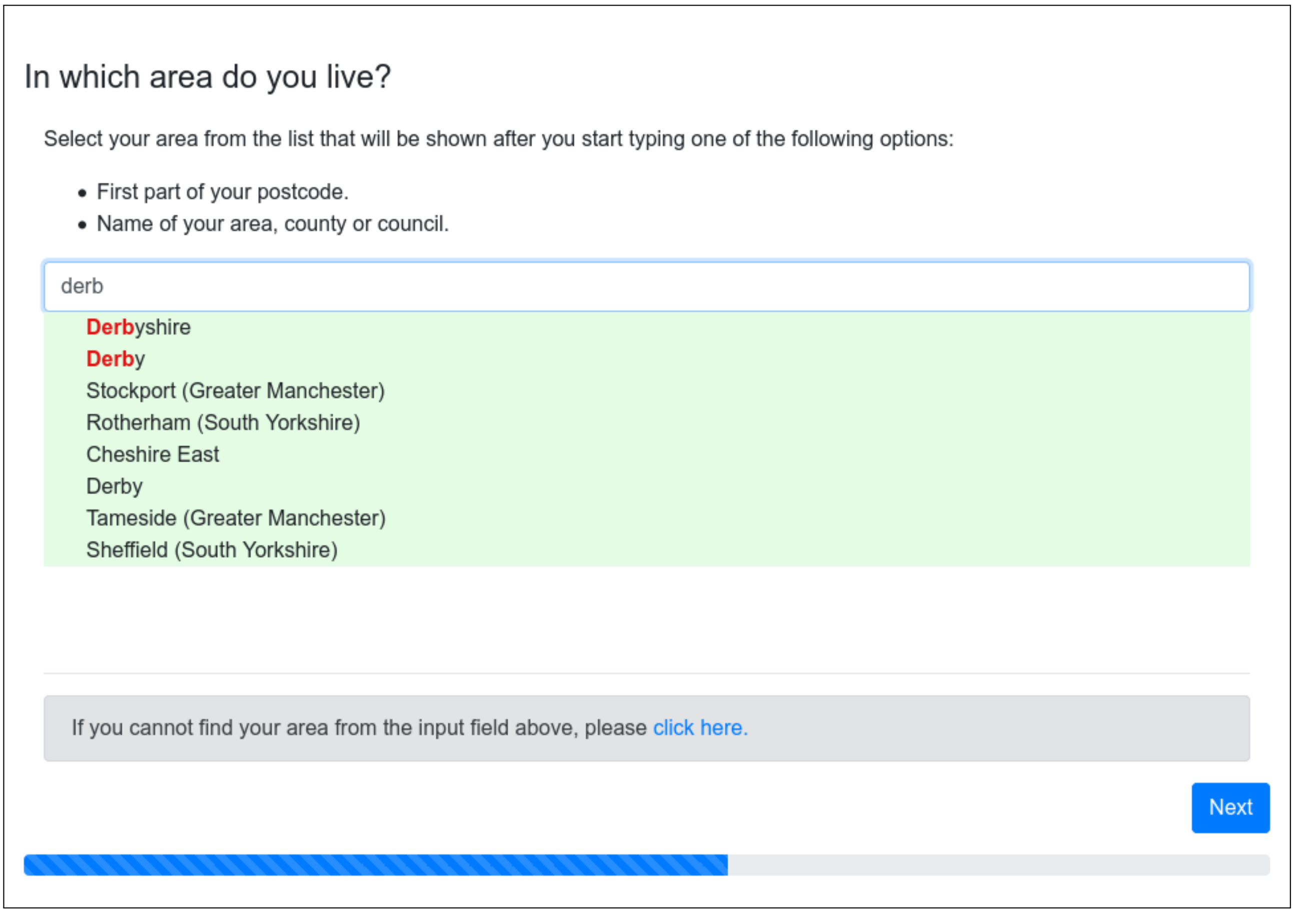

Figure H38: Experiences with COVID-19

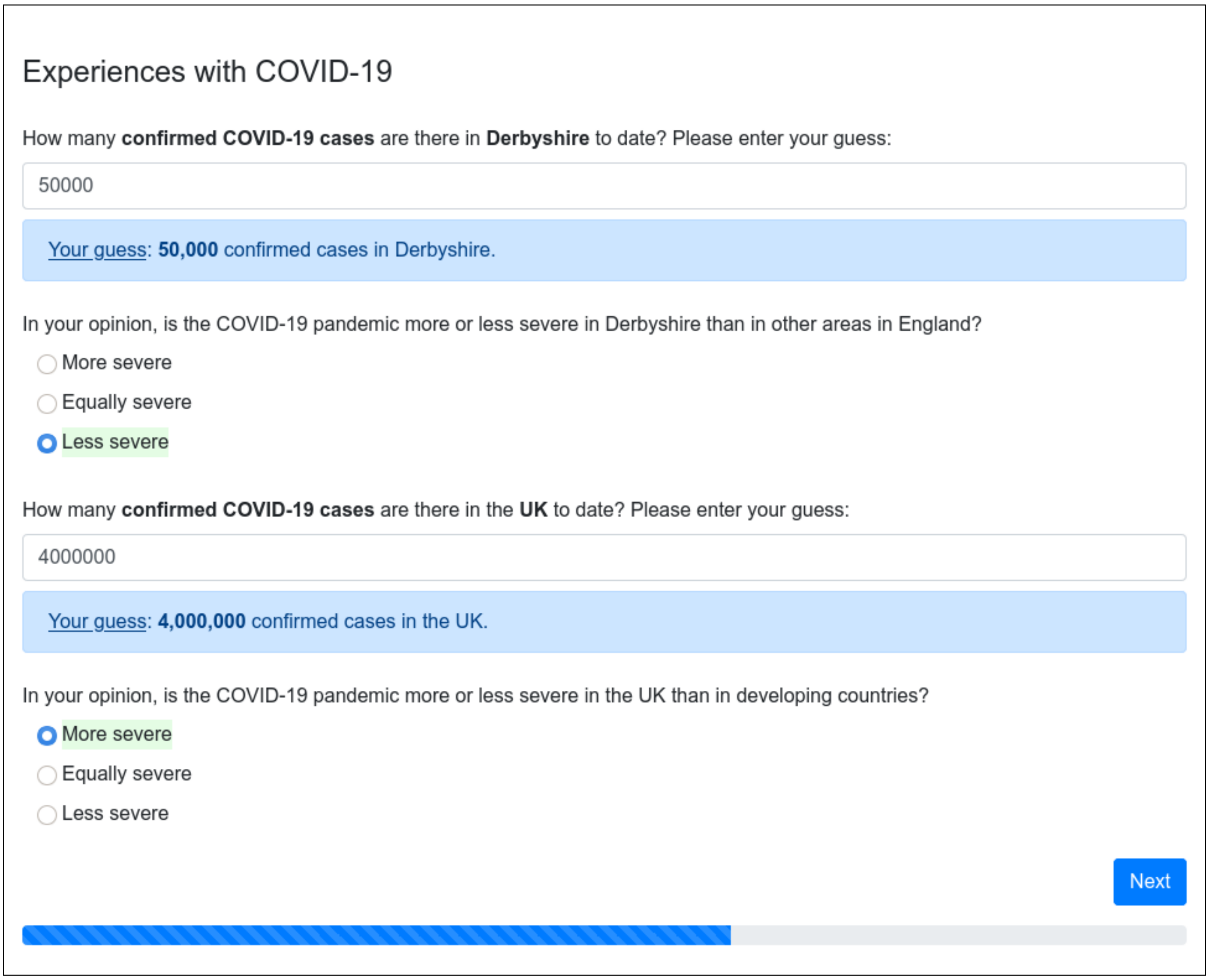

Experiences with COVID-19

How many **confirmed COVID-19 cases** are there in **Derbyshire** to date? Please enter your guess:

50000

Your guess: **50,000** confirmed cases in Derbyshire.

In your opinion, is the COVID-19 pandemic more or less severe in Derbyshire than in other areas in England?

- More severe
- Equally severe
- Less severe

How many **confirmed COVID-19 cases** are there in the **UK** to date? Please enter your guess:

4000000

Your guess: **4,000,000** confirmed cases in the UK.

In your opinion, is the COVID-19 pandemic more or less severe in the UK than in developing countries?

- More severe
- Equally severe
- Less severe

Next

Figure H39: Health situation

Your health situation since the outbreak of COVID-19

Has your health or the health of your family members been negatively affected by the COVID-19 pandemic?

- (●) A lot
- ( ) Somewhat
- ( ) Not at all

In the next 12 months, do you expect that your health or the health of your family members will be negatively affected by the Corona virus?

- ( ) A lot
- ( ) Somewhat
- (●) Not at all

Some people are at risk of becoming seriously ill if infected by COVID-19. What risk category do you belong to?

- ( ) High risk (clinically extremely vulnerable)*
- ( ) Moderate risk (clinically vulnerable)**
- (●) Neither of the above

NHS provides following information:

* People at **high risk** (clinically extremely vulnerable) from coronavirus include people who:

- have had an organ transplant
- are having chemotherapy or antibody treatment for cancer, including immunotherapy
- are having an intense course of radiotherapy (radical radiotherapy) for lung cancer
- are having targeted cancer treatments that can affect the immune system (such as protein kinase inhibitors or PARP inhibitors)
- have blood or bone marrow cancer (such as leukaemia, lymphoma or myeloma)
- have had a bone marrow or stem cell transplant in the past 6 months, or are still taking immunosuppressant medicine
- have been told by a doctor they you have a severe lung condition (such as cystic fibrosis, severe asthma or severe COPD)
- have a condition that means they have a very high risk of getting infections (such as SCID or sickle cell)
- are taking medicine that makes them much more likely to get infections (such as high doses of steroids)
- have a serious heart condition and are pregnant

** People at **moderate risk** (clinically vulnerable) from coronavirus include people who:

- are 70 or older
- are pregnant
- have a lung condition that's not severe (such as asthma, COPD, emphysema or bronchitis)
- have heart disease (such as heart failure)
- have diabetes
- have chronic kidney disease
- have liver disease (such as hepatitis)
- have a condition affecting the brain or nerves (such as Parkinson's disease, motor neurone disease, multiple sclerosis or cerebral palsy)
- have a condition that means they have a high risk of getting infections
- are taking medicine that can affect the immune system (such as low doses of steroids)
- are very obese (a BMI of 40 or above)

Next

Figure H40: Behavior since the outbreak of COVID-19

Behaviour since the outbreak of COVID-19

I have reduced the number of contacts with people outside my household:

- (x) A lot
- ( ) Somewhat
- ( ) Not at all

I have increased my stock of basic necessities, medication, or sanitary products:

- (x) A lot
- ( ) Somewhat
- ( ) Not at all

I wear a mask when I leave the house:

- (x) Always
- ( ) Sometimes
- ( ) Never

For which of the following activities did you leave your house in the last 4 weeks (multiple answers possible)?

- [x] Work
- [ ] Religious services
- [x] Restaurant or café
- [ ] Basic grocery shopping
- [x] Other shopping
- [ ] Walk a dog
- [x] Physical activity or leisure outdoors alone or with members of own household
- [ ] Physical activity or leisure outdoors with members of other households
- [x] Doctor or pharmacy
- [x] Meet friends or relatives
- [ ] Help neighbours or family in need
- [ ] Social gatherings or demonstrations

Do you follow the guidance and advice of the UK government on the COVID-19 pandemic?

- ( ) Always
- (x) Very Often
- ( ) Sometimes
- ( ) Rarely
- ( ) Never

To what extent do you agree with the following statement? I have done my bit to prevent and tackle COVID-19.

- ( ) Agree totally
- (x) Agree partly
- ( ) Neither agree nor disagree
- ( ) Disagree partly
- ( ) Disagree totally

Please specify what have you done to prevent and tackle COVID-19:

Reduce use of pubs / restaurants and stopped meeting friends

Next

Figure H41: Work situation

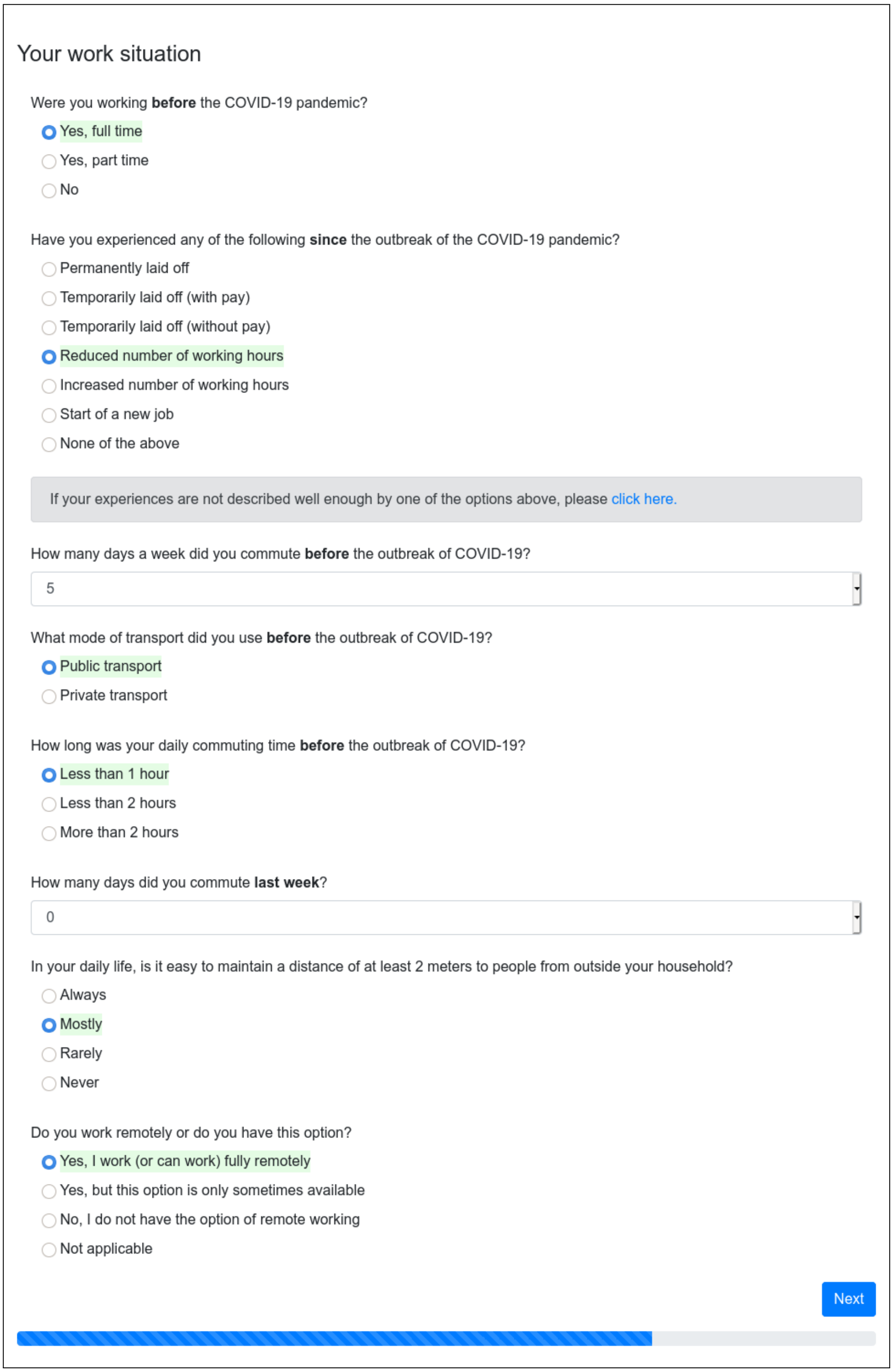

Your work situation

Were you working **before** the COVID-19 pandemic?

- (selected) Yes, full time
- Yes, part time
- No

Have you experienced any of the following **since** the outbreak of the COVID-19 pandemic?

- Permanently laid off
- Temporarily laid off (with pay)
- Temporarily laid off (without pay)
- (selected) Reduced number of working hours
- Increased number of working hours
- Start of a new job
- None of the above

If your experiences are not described well enough by one of the options above, please click here.

How many days a week did you commute **before** the outbreak of COVID-19?

5

What mode of transport did you use **before** the outbreak of COVID-19?

- (selected) Public transport
- Private transport

How long was your daily commuting time **before** the outbreak of COVID-19?

- (selected) Less than 1 hour
- Less than 2 hours
- More than 2 hours

How many days did you commute **last week**?

0

In your daily life, is it easy to maintain a distance of at least 2 meters to people from outside your household?

- Always
- (selected) Mostly
- Rarely
- Never

Do you work remotely or do you have this option?

- (selected) Yes, I work (or can work) fully remotely
- Yes, but this option is only sometimes available
- No, I do not have the option of remote working
- Not applicable

Next

Figure H42: Financial situation

Your financial situation

Think of the last 12 months before the COVID-19 pandemic

Your total monthly household income from all sources before tax was:

- ◯ Up to £2000
- ◯ More than £2000 up to £5000
- ◉ More than £5000 up to £10000
- ◯ More than £10000

Your household was able to make ends meet:

- ◯ With great difficulty
- ◯ With some difficulty
- ◉ Fairly easily
- ◯ Easily

Has your economic situation been affected by the COVID-19 pandemic?

Relative to the time **before** the COVID-19 pandemic, your household income has:

- ◯ Decreased a lot
- ◉ Decreased somewhat
- ◯ Stayed the same
- ◯ Increased somewhat
- ◯ Increased a lot

Your household is able to make ends meet:

- ◯ With great difficulty
- ◯ With some difficulty
- ◉ Fairly easily
- ◯ Easily

In the next 12 months, do you expect that your economic situation will change?

Relative to the time **before** the COVID-19 pandemic, do you expect your household income will:

- ◯ Decrease a lot
- ◯ Decrease somewhat
- ◯ Stay the same
- ◉ Increase somewhat
- ◯ Increase a lot

Next

Figure H43: Economic situation

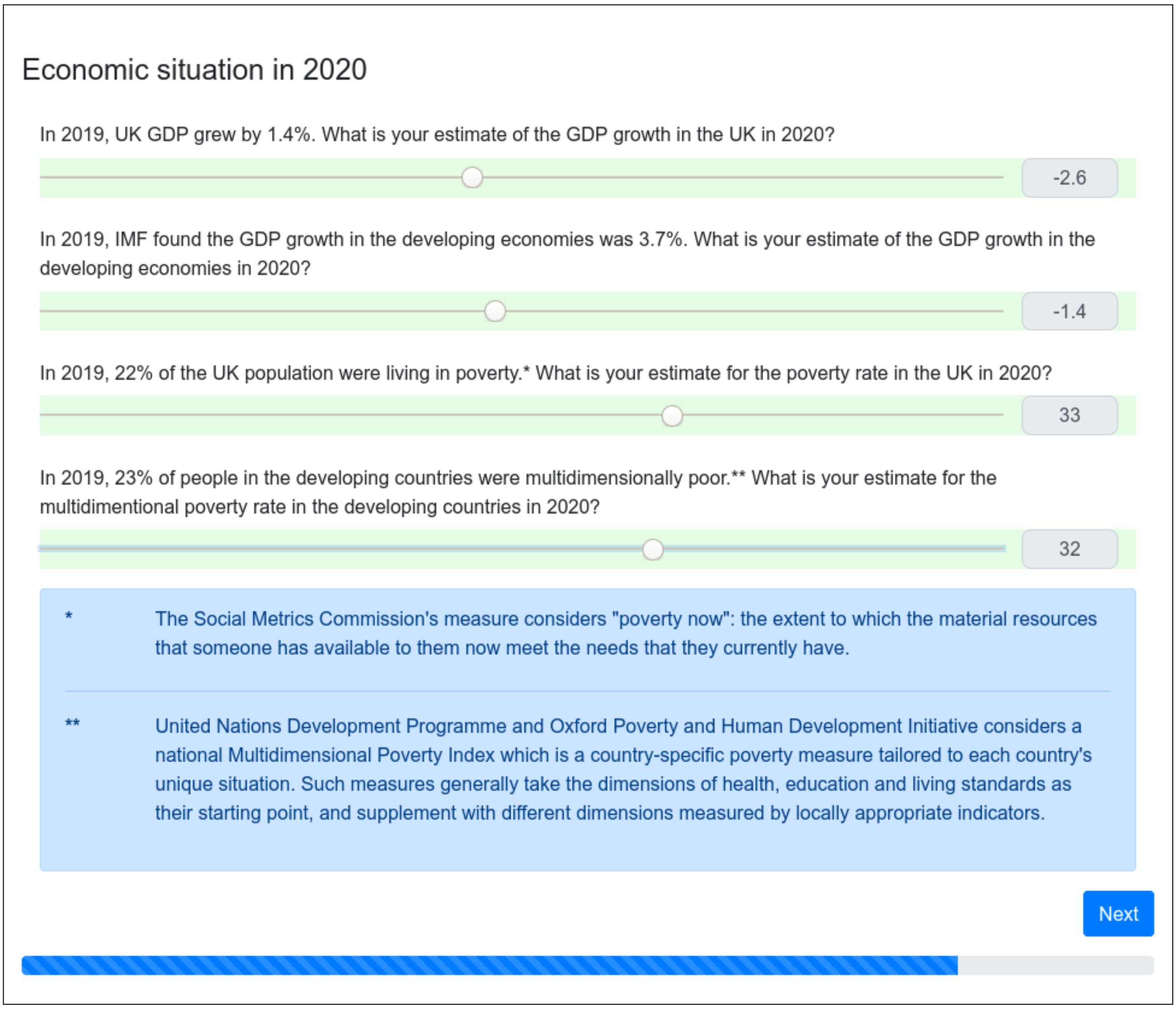

Economic situation in 2020

In 2019, UK GDP grew by 1.4%. What is your estimate of the GDP growth in the UK in 2020?

-2.6

In 2019, IMF found the GDP growth in the developing economies was 3.7%. What is your estimate of the GDP growth in the developing economies in 2020?

-1.4

In 2019, 22% of the UK population were living in poverty.* What is your estimate for the poverty rate in the UK in 2020?

33

In 2019, 23% of people in the developing countries were multidimensionally poor.** What is your estimate for the multidimentional poverty rate in the developing countries in 2020?

32

* The Social Metrics Commission's measure considers "poverty now": the extent to which the material resources that someone has available to them now meet the needs that they currently have.

** United Nations Development Programme and Oxford Poverty and Human Development Initiative considers a national Multidimensional Poverty Index which is a country-specific poverty measure tailored to each country's unique situation. Such measures generally take the dimensions of health, education and living standards as their starting point, and supplement with different dimensions measured by locally appropriate indicators.

Next

Figure H44: Main news sources

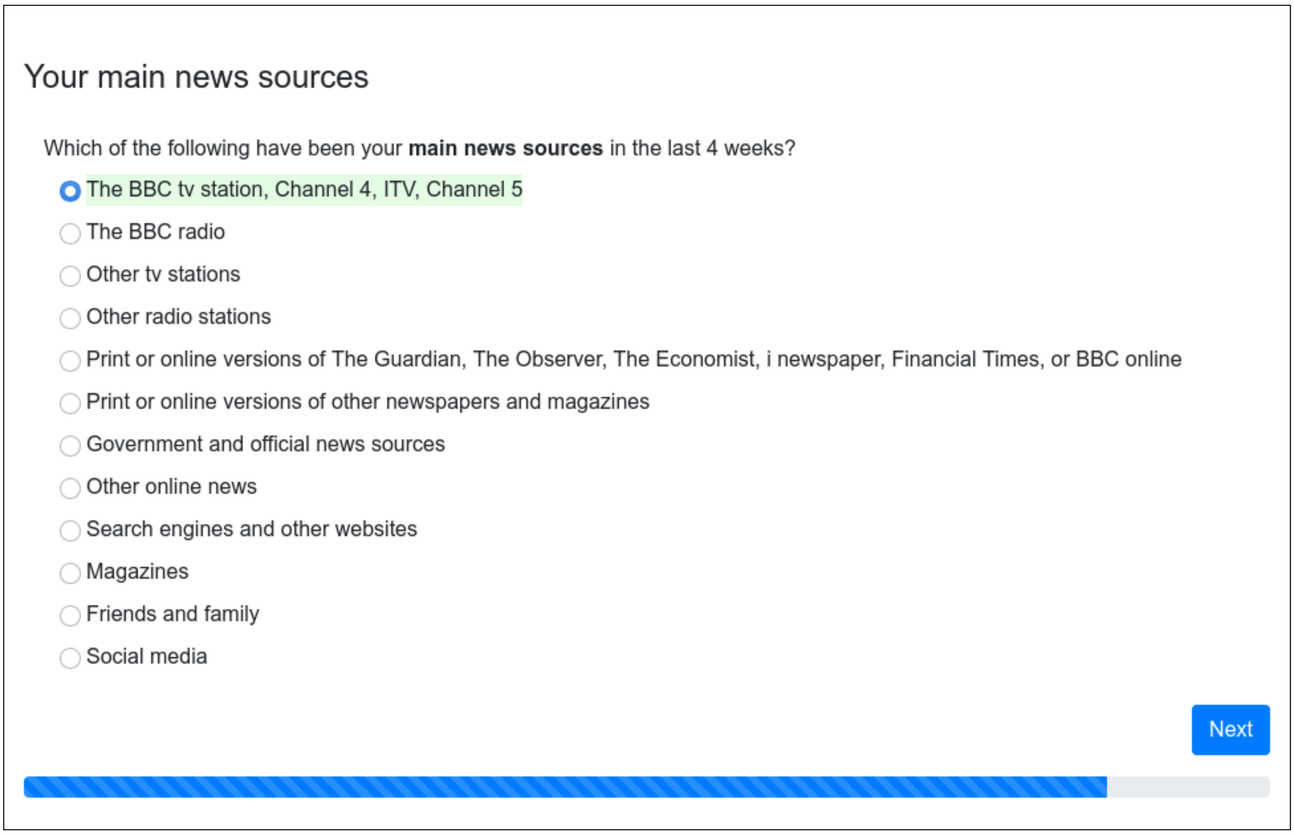

Your main news sources

Which of the following have been your **main news sources** in the last 4 weeks?

- ◉ The BBC tv station, Channel 4, ITV, Channel 5
- ○ The BBC radio
- ○ Other tv stations
- ○ Other radio stations
- ○ Print or online versions of The Guardian, The Observer, The Economist, i newspaper, Financial Times, or BBC online
- ○ Print or online versions of other newspapers and magazines
- ○ Government and official news sources
- ○ Other online news
- ○ Search engines and other websites
- ○ Magazines
- ○ Friends and family
- ○ Social media

Next

Figure H45: COVID-19 risks and policies

COVID-19 risks and policies to counteract the pandemic

What do you think about the risks from COVID-19?

- ◯ Most people have no symptoms or only few flu-like symptoms; otherwise COVID-19 isn't very dangerous.
- ◯ Only people with several pre-existing conditions die after getting COVID-19 and any of those conditions could have been the reason for their death.
- ◯ Older members of the population and risk groups can become seriously ill after infection with COVID-19.
- ◉ Anyone can become seriously ill after infection with COVID-19.
- ◯ Many people infected with COVID-19 become seriously ill.
- ◯ Most people infected with COVID-19 become seriously ill.

What do you think about the lockdown to contain the spread of COVID-19?

- ◯ There shouldn't have been any lockdown; it is not clear whether it helps at all.
- ◯ The lockdown is damaging to the economy and the health benefits of the lockdown do not outweigh the economic risks.
- ◯ The lockdown is harming the economy and the health benefits of the lockdown might not outweigh the economic risks.
- ◉ The lockdown is necessary to contain the spread of COVID-19.
- ◯ No measure is too costly to contain the spread of COVID-19.

The policies against the spread of COVID-19 taken by the UK government are overall:

- ◯ Much too lax
- ◉ Somewhat too lax
- ◯ Just right
- ◯ Somewhat too harsh
- ◯ Much too harsh

With which statement do you agree more?

- ◯ The Coronavirus is man-made.
- ◉ The Coronavirus is natural in origin.

With which statement do you agree more?

- ◯ The Coronavirus was spread on purpose.
- ◉ The Coronavirus was spread unintentionally.

When do you think the COVID-19 pandemic will be over in the UK?

In [3] months

Have you received a COVID-19 vaccination yet?

- ◯ No
- ◉ Yes, one shot
- ◯ Yes, two shots

Are you worried that new variants of COVID-19 will make it difficult to end the pandemic?

- ◯ Yes, very worried
- ◉ Yes, somewhat worried
- ◯ Not sure
- ◯ No, not very worried
- ◯ No, not worried at all

Next

Figure H46: Empathy

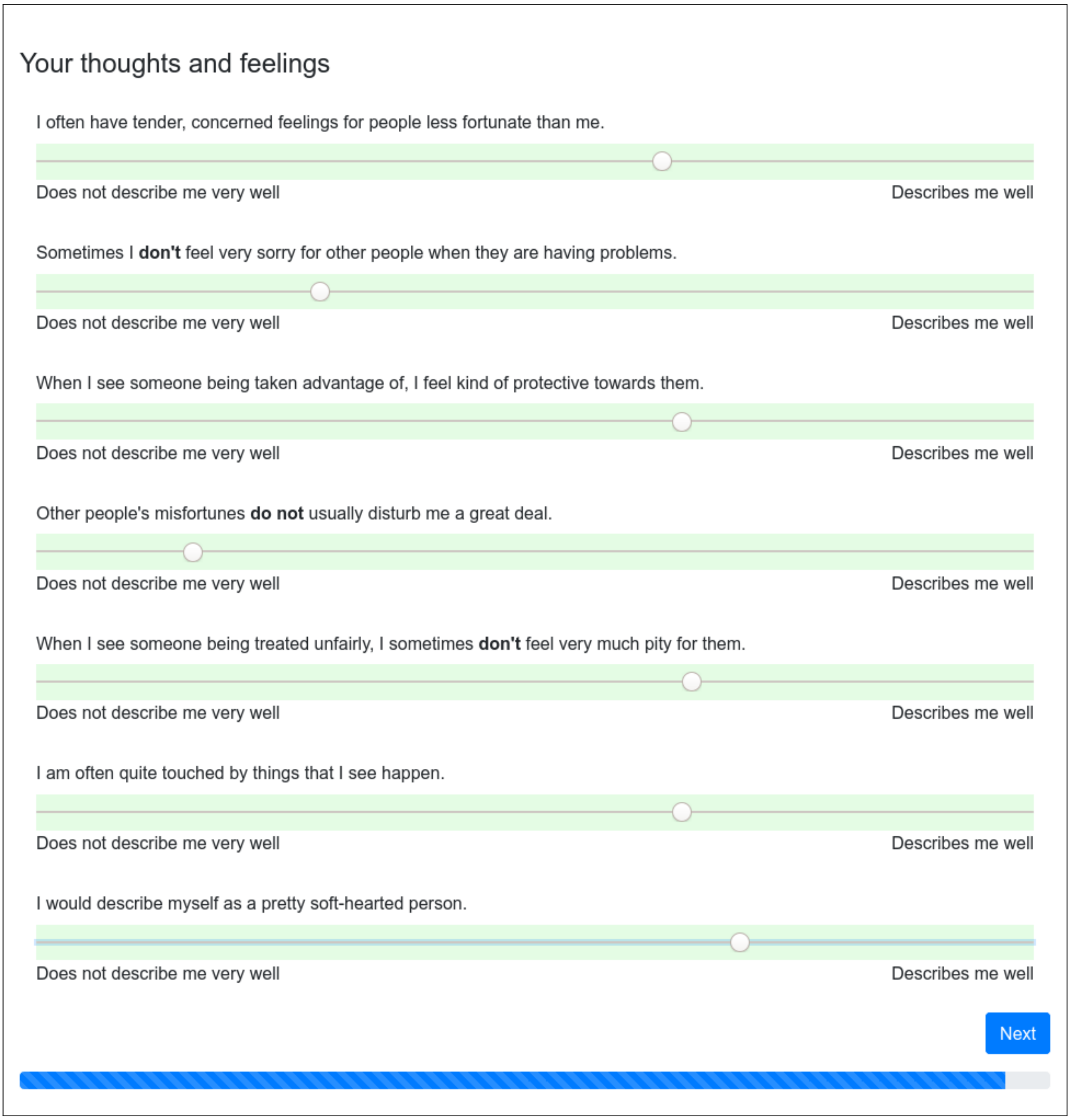

Your thoughts and feelings

I often have tender, concerned feelings for people less fortunate than me.

Does not describe me very well — Describes me well

Sometimes I **don't** feel very sorry for other people when they are having problems.

Does not describe me very well — Describes me well

When I see someone being taken advantage of, I feel kind of protective towards them.

Does not describe me very well — Describes me well

Other people's misfortunes **do not** usually disturb me a great deal.

Does not describe me very well — Describes me well

When I see someone being treated unfairly, I sometimes **don't** feel very much pity for them.

Does not describe me very well — Describes me well

I am often quite touched by things that I see happen.

Does not describe me very well — Describes me well

I would describe myself as a pretty soft-hearted person.

Does not describe me very well — Describes me well

Next

Figure H47: Payment info

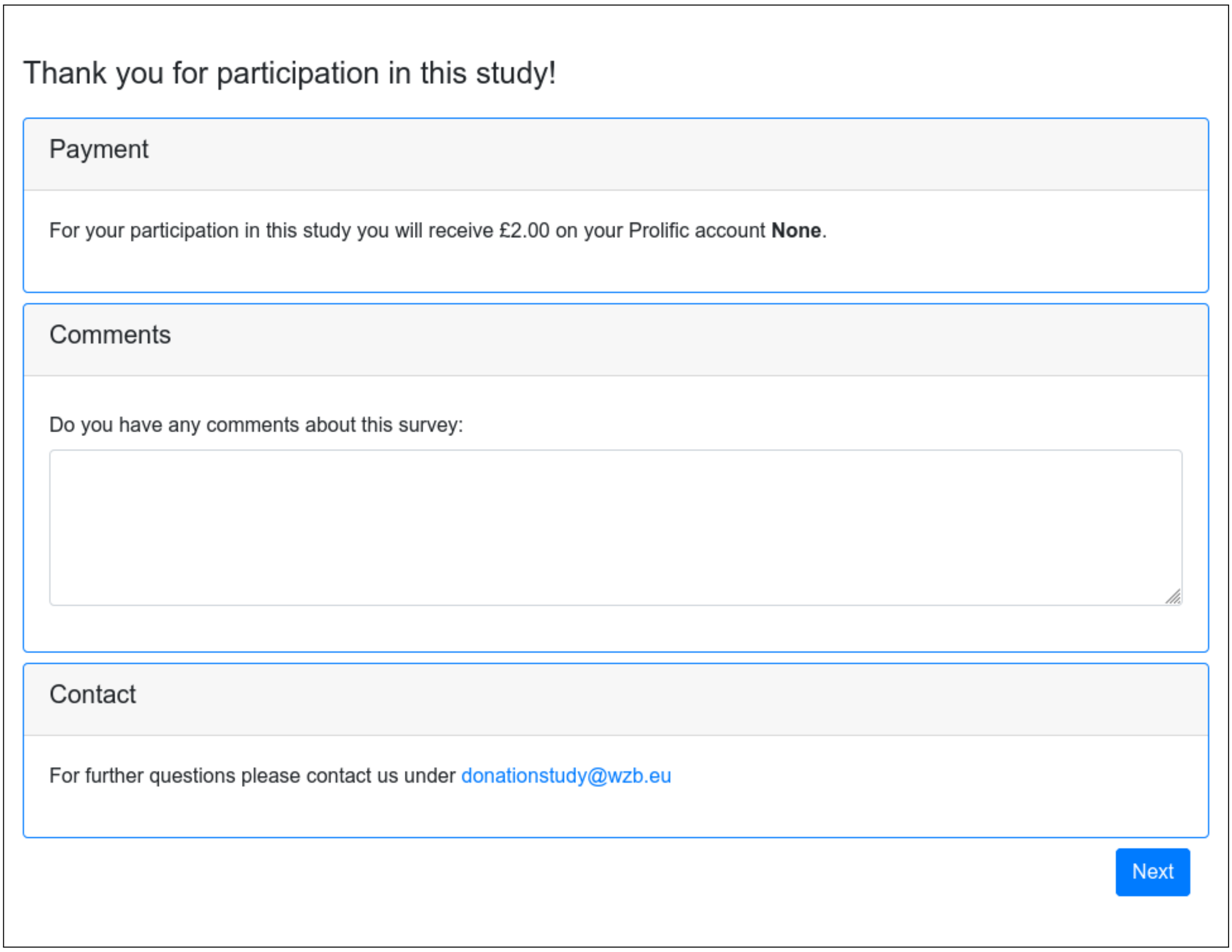

References

Andreoni, J., Rao, J. M., & Trachtman, H. (2017). Avoiding the ask: A field experiment on altruism, empathy, and charitable giving. *Journal of Political Economy*, *125*(3), 625–653. https://doi.org/10.1086/691703

DellaVigna, S., List, J. A., & Malmendier, U. (2012). Testing for altruism and social pressure in charitable giving. *The quarterly journal of economics*, *127*(1), 1–56. https://doi.org//10.1093/qje/qjr050

Scherer, K. R. (2005). What are emotions? And how can they be measured? *Social science information*, *44*(4), 695–729. https://doi.org/10.1177/0539018405058216

Scherer, K. R., Shuman, V., Fontaine, J., & Soriano Salinas, C. (2013). The GRID meets the Wheel: Assessing emotional feeling via self-report. *Components of emotional meaning: A sourcebook*. https://doi.org/10.1093/acprof:oso/9780199592746.003.0019
